# MRSL: a causal network pruning algorithm based on GWAS summary data

**DOI:** 10.1093/bib/bbae086

**Published:** 2024-03-14

**Authors:** Lei Hou, Zhi Geng, Zhongshang Yuan, Xu Shi, Chuan Wang, Feng Chen, Hongkai Li, Fuzhong Xue

**Affiliations:** Beijing International Center for Mathematical Research, Peking University, Beijing, People’s Republic of China, 100871; School of Mathematics and Statistics, Beijing Technology and Business University, Beijing, People’s Republic of China, 100048; Department of Epidemiology and Health Statistics, School of Public Health, Cheeloo College of Medicine, Shandong University, Jinan, People’s Republic of China, 250000; Institute for Medical Dataology, Cheeloo College of Medicine, Shandong University, Jinan, People’s Republic of China, 250000; Department of Biostatistics, University of Michigan, Ann Arbor, USA; Qilu Hospital, Cheeloo College of Medicine, Shandong University, Jinan, People's Republic of China, 250000; School of Public Health, Nanjing Medical University, Nanjing, China, 211166; Department of Epidemiology and Health Statistics, School of Public Health, Cheeloo College of Medicine, Shandong University, Jinan, People’s Republic of China, 250000; Institute for Medical Dataology, Cheeloo College of Medicine, Shandong University, Jinan, People’s Republic of China, 250000; Department of Epidemiology and Health Statistics, School of Public Health, Cheeloo College of Medicine, Shandong University, Jinan, People’s Republic of China, 250000; Institute for Medical Dataology, Cheeloo College of Medicine, Shandong University, Jinan, People’s Republic of China, 250000; Qilu Hospital, Cheeloo College of Medicine, Shandong University, Jinan, People's Republic of China, 250000

**Keywords:** network pruning, causal discovery, mendelian randomization, graph theory, serum metabolites, esophageal squamous cell carcinoma

## Abstract

Causal discovery is a powerful tool to disclose underlying structures by analyzing purely observational data. Genetic variants can provide useful complementary information for structure learning. Recently, Mendelian randomization (MR) studies have provided abundant marginal causal relationships of traits. Here, we propose a causal network pruning algorithm MRSL (MR-based structure learning algorithm) based on these marginal causal relationships. MRSL combines the graph theory with multivariable MR to learn the conditional causal structure using only genome-wide association analyses (GWAS) summary statistics. Specifically, MRSL utilizes topological sorting to improve the precision of structure learning. It proposes MR-separation instead of d-separation and three candidates of sufficient separating set for MR-separation. The results of simulations revealed that MRSL had up to 2-fold higher F1 score and 100 times faster computing time than other eight competitive methods. Furthermore, we applied MRSL to 26 biomarkers and 44 International Classification of Diseases 10 (ICD10)-defined diseases using GWAS summary data from UK Biobank. The results cover most of the expected causal links that have biological interpretations and several new links supported by clinical case reports or previous observational literatures.

## INTRODUCTION

Causal discovery aims to infer causal structure by analyzing purely observational data [1, 2]. It can be widely applied in the social and natural sciences, and it is a powerful tool for discovering biological networks [3, 4], disease diagnostics [5, 6], etc. In recent years, there has been a significant accumulation of large datasets in population-based genome-wide association analyses (GWAS), with extensive phenotypic and genotypic data from the same subjects [7–9]. The use of genetic variants provides a new insight into causal discovery [10–13]. Genetic variants are allocated at gamete formation during conception and thus cannot be affected by phenotypes [8, 9]. This prior information improves the accuracy of learning the Bayesian network (BN) [10]. On the other hand, genetic variants can also be considered instrumental variables (IVs) for inferring causal relationships between an exposure and outcome, a method known as Mendelian randomization (MR) [8, 9]. MR is used to control unmeasured confounding and avoid reverse causality. Single-sample MR involves estimating the genetic associations in the same dataset. In contrast, two-sample MR entails estimating the genetic associations of the exposure and outcome in different datasets [14], assuming that both populations are compatible. Estimation of the causal effect of a single exposure on one outcome is referred to as univariable MR (UVMR). In contrast, multivariable MR (MVMR) entails estimating the causal effects of multiple exposures on one outcome, which requires multiple GWAS samples. Numerous MR studies have been published to investigate causal relationships among traits in recent years. An intuitive question arises: can we fully leverage these findings to construct a causal network? Published MR studies continue to focus on the causal relationships between single exposure (UVMR) or multiple exposures (MVMR) [15] on an outcome, with assumed roles for variables such as exposure, outcome and covariates. However, in a complex network, the causal relationships of variables often remain unclear. Therefore, learning the conditional causal structure of variables based on MR using GWAS summary data remains a significant challenge.

Some methods have been proposed for learning causal structures incorporating genetic variants. Richard *et al*. [10] incorporated prior information about genetic variants into black and white lists to improve the performance of traditional BN network learning. Badsha *et al.* [11] introduced a machine learning algorithm named MRPC that incorporates the principle of MR (PMR) into the PC algorithm for learning causal graphs. Nevertheless, both algorithms require the causal sufficiency assumption, meaning there should be no unobserved confounders among all the variables. In the context of MR, David *et al.* [12] introduced a pipeline called Causal Graphical Analysis Using Genetics (cGAUGE), which uses IV filters: ImpIV and UniqueIV, to select valid IVs for UVMR. Subsequently, it constructs a marginal causal graph in which edges represent the total effects for each pair of variables. cGAUGE allows the unobserved confounders among all the variables but still requires individual genetic and phenotypic data. Another type of causal graph, conditional causal graph, is better suited for elucidating biological mechanisms in medicine. In a conditional causal graph, edges represent direct effects for each pair of variables, not through mediators and confounders in the sufficient separating set. Brown *et al.* [13] proposed a flexible two-stage procedure called bidirectional mediated MR (BIMMER), to infer sparse networks of direct causal effects (DCEs) from phenome-scale GWAS summary statistics. However, the examination of the causal relationship between two variables should condition on all the other variables regardless of their roles (e.g. mediator, confounder or collider).

This paper proposes MRSL, a causal network pruning algorithm that leverages graph theory and MVMR [15–17] for structural learning using summarized genetic data without requiring individual data. ‘Pruning’ means removing the spurious direct edges in the marginal causal graph, which can be obtained using bi-directional MR [18, 19] in pairs or summarizing the results from published MR studies. The edges in a marginal causal graph represent the total causal relationships for each pair of variables and contain lots of spurious direct edges. MRSL aims to remove these spurious direct edges and obtain a conditional causal graph. We conducted numerous simulations to evaluate the performance of MRSL and compared it to eight commonly used methods. Furthermore, we applied MRSL to 26 biomarkers and 44 International Classification of Diseases 10 (ICD10)-defined diseases in 337 198 Europeans in the UK Biobank.

## MATERIALS AND METHODS

### MRSL model

**Figure 1:**
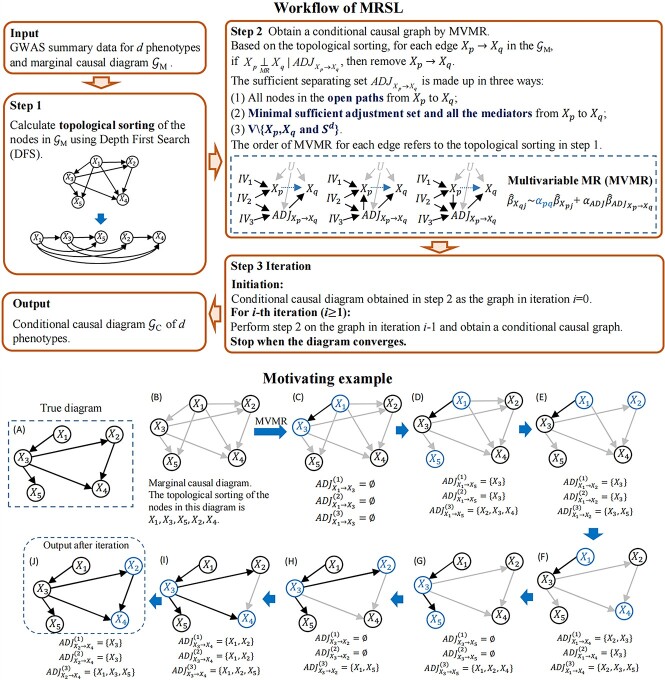
Workflow and the motivating example of MRSL algorithm. Confounders of *d* phenotypes are omitted. The input includes GWAS summary data for each phenotype and marginal causal graph. For step 1, the topological sorting of marginal causal graph should be found using Depth First Search (DFS). For step 2, MVMR is performed to remove extra edges in the marginal causal graph by adjusting for the genetic associations with phenotypes of three strategies of sufficient separating sets. Then a conditional causal graph is obtained. In step 3, iteration for step 2 is performed until the graph converges. Finally, MRSL outputs an estimated conditional causal graph. (**A**–**J**) Motivating example with five nodes. (A) The true causal graph. (B) Marginal causal graph. (C–J) Perform MVMR for each edge in graph (F) based on its topological sorting. MRSL outputs the graph (J). $AD{J}_{X_p\to{X}_q}$ denotes the sufficient separating set from ${X}_p$ to ${X}_q$. $AD{J}_{X_p\to{X}_q}^{(1)}$ includes all nodes on the open paths from ${X}_p$ to ${X}_q$; (2) $AD{J}_{X_p\to{X}_q}^{(2)}$ includes the elements in the minimal sufficient adjustment set and all the mediators from ${X}_p$ to ${X}_q$; (3) $AD{J}_{X_p\to{X}_q}^{(3)}$=V\{${X}_p$,${X}_q$ and${S}^d$}. ${S}^d$ refers to the colliders where ${X}_p$ and ${X}_q$ have direct edges on them. In the motivating example, we omit the unobserved confounders U and the instrumental variables for each phenotype used in MR.

The workflow of MRSL is displayed in Figure 1. Assume a DAG 𝒢$=<V,E>$ with unobserved confounders *U*, where *V* is a set of nodes and *E* is a set of paired nodes connected by edges. Assume we are interested in *d* phenotypes $\left\{{X}_1,{X}_2,...,{X}_d\right\}$. For convenience, the unobserved confounders among phenotypes in Figure 1 are omitted. The input comprises two components: GWAS summary data and the marginal causal graph 𝒢_M_ for *d* phenotypes. GWAS summary data for these *d* phenotypes are publicly accessible from the GWAS catalog, mr-base, etc. The marginal causal graph 𝒢_M_ can be obtained through pairwise bi-directional MR or by directly summarizing the results from published MR studies or other causal evidence.

First, we introduce the following three assumptions and two lemmas:

Assumption 1. (Causal Markov condition). Each variable is independent of its non-descendants given its parents in graph 𝒢.

Assumption 2. (Faithfulness assumption). All independencies embedded in the observed distribution ℙ are stable and invariant to changes in the parameterization. Thus, it implies (together with d-separation) that $\left({X}_p\perp{X}_q|{X}_a\right)$_ℙ_$\iff \left({X}_p\perp{X}_q|{X}_a\right)$_𝒢_.

Assumption 3. (Valid IVs in MVMR). For an exposure, a set of covariates, and an outcome, the valid IVs in MVMR must be strongly associated with at least one of the exposure or covariates (Relevance), be independent of unobserved confounders among the exposure, covariates and the outcome (Exchangeability) and affect the outcome only through the exposure or covariates (Exclusion restriction).

Lemma 1. For the true causal graph 𝒢 and the marginal causal graph 𝒢_M_, E_𝒢_⊆E_𝒢M_ and S_𝒢_⊆S_𝒢M_, where E_𝒢_ and E_𝒢M_ denote all the paired nodes for directed edges in 𝒢 and 𝒢_M_, respectively. S_𝒢_ and S_𝒢M_ denote the colliders in 𝒢 and 𝒢_M_, respectively.

Lemma 2. (Topological sorting invariance). The topological sorting of the true causal graph 𝒢 and the marginal causal graph 𝒢_M_ are the same *T*_𝒢_*= T*_𝒢M_.

Assumption 1 defines conditional independence in the graph. Assumption 2 ensures that the set of independence relations derived from the causal Markov condition is exactly the set that holds for the network. These two assumptions establish a connection between the statistical test and the graph, enabling us to employ statistical tools for structure learning. Lemma 1 states all the edges/colliders in 𝒢 must be included in 𝒢_M_, and Lemma 2 emphasizes the topological sorting consistency between 𝒢 and 𝒢_M_. These lemmas underscore the significance of the accuracy of 𝒢_M_ in MRSL because MRSL is a pruning algorithm that solely removes spurious edges from 𝒢_M_ without adding new edges.

The second step of MRSL is pivotal and focuses on eliminating E_𝒢M_\E_𝒢_ within 𝒢_M_ through conditional independence. We propose a novel criterion for conditional independence known as MR-separation based on MVMR. MVMR aims to explore the direct causal effects of multiple exposures on one outcome using multiple GWAS summary datasets. It divides the total effect of the main exposure (${X}_p$) on the outcome (${X}_q$) into direct effect (${X}_p\to{X}_q$) and indirect effect through other exposures (${X}_p\to AD{J}_{X_p\to{X}_q}\to{X}_q$ or ${X}_p\leftarrow AD{J}_{X_p\to{X}_q}\to{X}_q$) by regressing genetic associations with ${X}_q$ on ${X}_p$, adjusting for genetic associations with $AD{J}_{X_p\to{X}_q}$:


(1) \begin{equation*} {\hat{\beta}}_{X_{qj}}={a}_{p\to q}{\hat{\beta}}_{X_{pj}}+{a}_{AD J}{\hat{\beta}}_{X_{AD{J}_{X_p\to{X}_q}j}}+{\varepsilon}_{X_{qj}},{\varepsilon}_{X_{qj}}\sim N\left(0, se{\left({\hat{\beta}}_{X_{qj}}\right)}^2\right), \end{equation*}

where ${a}_{p\to q}$ is the direct causal effect from ${X}_p$ to ${X}_q$. If ${a}_{p\to q}=0$, then ${X}_p$ and ${X}_q$ are said to be blocked by $AD{J}_{X_p\to{X}_q}$. ${\hat{\beta}}_{X_{qj}},{\hat{\beta}}_{X_{pj}}$ and ${\hat{\beta}}_{X_{AD{J}_{X_p\to{X}_q}j}}$ are genetic associations with ${X}_q$, ${X}_p$ and $AD{J}_{X_p\to{X}_q}$ from multiple GWAS summary datasets. For a continuous variable, the beta coefficient and its standard error can be obtained from linear regression. For a binary variable, the log odds ratio (OR) coefficient and its standard error can be obtained from logistic regression. The ${a}_{p\to q}$ can be estimated by generalized least squares method. The IVs for the model (1) above must satisfy Assumption 3. Details of UVMR and MVMR are shown in the Supplemental Material and Methods S1. We then define MR-separation as follows:

Definition 1. (MR-separation) For two variables ${X}_p$ and ${X}_q$, under Assumption 3, if ${X}_p$ and ${X}_q$ are causally independent with each other given a sufficient separating set $AD{J}_{X_p\to{X}_q}$ based on model (1), that is, the direct causal relationship from ${X}_p$ to ${X}_q$ is zero (${a}_{p\to q}=0$), then ${X}_p$ and ${X}_q$ are MR-separated by the sufficient separating set $AD{J}_{X_p\to{X}_q}$, that is ${X}_p\underset{MR}{\perp }{X}_q\mid AD{J}_{X_p\to{X}_q}$.

Theorem 1. Under Assumptions 1–3, for each edge ${X}_p\to{X}_q$ in the marginal causal graph 𝒢_M_, if there is a sufficient separating set $AD{J}_{X_p\to{X}_q}$ such that ${X}_p$ and ${X}_q$ are MR-separated, i.e. ${X}_p\underset{MR}{\perp }{X}_q\mid AD{J}_{X_p\to{X}_q}$, then there is no direct edge from ${X}_p$ to ${X}_q$ in the true causal graph 𝒢.

It is worth noting that MR-separation examines the conditional independence between two variables using MVMR accounting for unmeasured confounding, whereas d-separation requires no unmeasured confounding assumption. Theorem 1 states that the edges E_𝒢M_\ E_𝒢_ in the graph 𝒢_M_ can be removed by MR-separation. Details of the proof are shown in the Supplemental Material and Methods S2–S4. In the second step, for the paired nodes of each edge in 𝒢_M_ (e.g. ${X}_p\to{X}_q$), we assessed whether they are MR-separated by a sufficient separating set $AD{J}_{X_p\to{X}_q}$. We provided three candidate sets of $AD{J}_{X_p\to{X}_q}$: (1) all nodes on the open paths from ${X}_p$ to ${X}_q$; (2) minimal sufficient adjustment set [1, 2] for confounders and all the mediators from ${X}_p$ to ${X}_q$; and (3) V\{${X}_p$,${X}_q$ and ${S}^d$}. ${S}^d$ refers to a set of colliders where the two interested nodes have direct edges on them. For instance, for two nodes ${X}_p$ and ${X}_q$, the collider ${S}_1$ in ${X}_p\to{S}_1\leftarrow{X}_q$ is included in ${S}^d$, but the collider ${S}_2$ in ${X}_p\to{S}_2\leftarrow C\to{X}_q$ is not included in ${S}^d$. ${S}^d$ in the graph 𝒢_M_ includes the colliders and the nodes not on the pathway from ${X}_p$ to ${X}_q$ but does not include any mediators, confounders or the nodes that are both mediators and confounders on the pathways from ${X}_p$ to ${X}_q$ in the true causal graph 𝒢.

The topological sorting *T*_𝒢M_, calculated using depth first search (DFS) [20, 21] (as described in the Supplemental Material and Methods S5), enhances the pruning speed of MRSL. An illustrated example is provided in Figure S47. Following the second step, we added an iteration process, repeating step 2 until the graph converges. This step aimed to mitigate random errors and statistical testing errors in MVMR, thus improving the precision of MRSL.

We provided a motivating example to illustrate the workflow of MRSL (Figure 1A–J). The true causal diagram is shown in Figure 1A; the inputs are GWAS summary datasets of five phenotypes and the marginal causal graph of five variables (Figure 1B); the topological sorting is $\left\{{X}_1,{X}_3,{X}_5,{X}_2,{X}_4\right\}$. Next, we conducted MVMR analyses across each edge to detect whether the edge is spurious (Figure 1B). During this stage, we controlled for the genetic associations with phenotypes in $AD{J}_{X_p\to{X}_q}$ for each MVMR. On the basis of topological sorting, we firstly focused on the edge ${X}_1\to{X}_3$. The $AD{J}_{X_1\to{X}_3}$ was an empty set; thus, this edge was retained. Then, we focus on the edge ${X}_1\to{X}_5$, $AD{J}_{X_1\to{X}_5}^{(1)}= AD{J}_{X_1\to{X}_5}^{(2)}=\left\{{X}_3\right\}$ and $AD{J}_{X_1\to{X}_5}^{(3)}=\left\{{X}_2,{X}_3,{X}_4\right\}$. MVMR was performed by adjusting for the genetic associations with phenotypes in $AD{J}_{X_p\to{X}_q}$, and the result revealed a null direct causal relationship between ${X}_1$ and ${X}_5$, indicating that ${X}_1$ and ${X}_5$ are MR-separated by $AD{J}_{X_p\to{X}_q}$. Hence, the edge ${X}_1\to{X}_5$ is removed. The remaining edges were tested in the same manners (Figure 1C–J). Figure 1J was obtained after testing all the edges in Figure 1B once. An iteration of step 2 was conducted using this graph, and it terminated when the causal graph reached convergence. Finally, MRSL outputs the target conditional causal diagram.

### Simulations

We conducted a series of simulation studies to evaluate the performance of MRSL. A crucial part of MRSL is step 2, which utilizes MVMR to eliminate spurious direct edges in the marginal causal graph. The efficacy of MRSL hinges on the performance of MVMR; thus, our initial focus was on conducting a simulation study to find the most optimal IVs selection strategy such that the performance of MVMR is best when adjusting for the collider, mediator and confounder. In the subsequent simulations, based on the above optimal IV selection strategy, we compared the performance of MRSL with eight published methods in structure learning of generated random and fixed graphs. As a sensitivity analysis, we conducted an evaluation to assess the robustness of MRSL in the presence of invalid IVs.

#### Simulation study 1 on IVs selection in MVMR

**Figure 2:**
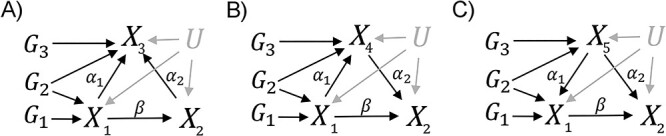
Diagrams for simulation study 1. ${X}_1$, exposure; ${X}_2$, outcome; ${X}_3$, collider; ${X}_4$, mediator; ${X}_5$, measured confounder; U, unobserved confounder. $\beta$ is the causal effect of ${X}_1$ on ${X}_2$. ${\alpha}_1$ is the causal effect of ${X}_1$ on ${X}_3$/${X}_4$ or ${X}_5$ on ${X}_1$. ${\alpha}_2$ is the causal effect of ${X}_5$/${X}_4$ on ${X}_2$ or ${X}_2$ on ${X}_3$. ${G}_1$ are SNPs only associated with ${X}_1$. ${G}_2$ are SNPs associated with ${X}_1$ and adjusting variable. ${G}_3$ are SNPs only associated with the adjusting variable.

The basis of MRSL is MVMR; thus, it is vital to select valid IVs. First, we conducted a simulation study to evaluate the performance of MVMR when estimating the causal effect of the interested exposure (${X}_1$) on an outcome (${X}_2$). We considered three roles of another exposure in MVMR: a collider (${X}_3$), mediator (${X}_4$) or measured confounder (${X}_5$) in the causal pathway from ${X}_1$ to ${X}_2$ (Figure 2A–C). Based on the three figures, two kinds of candidate IVs can be considered: (1) union: the SNPs associated with at least one of the multiple exposures (${G}_1+{G}_2+{G}_3$) and (2) intersection: the SNPs associated with all the exposures simultaneously (${G}_1+{G}_2$for collider and mediator, ${G}_2+{G}_3$for measured confounder). When another exposure is a confounder, ${G}_3$ is also associated with ${X}_1$, and it may be selected as IV because practitioners do not know its true role. Similarly, when another exposure is a mediator, ${G}_1$ is also associated with ${X}_4$. We generated 10 000 independent individuals for each variable and 1000 repeated datasets. To assess the performance of MVMR, we plotted a boxplot to evaluate the estimation of the causal effect of ${X}_1$ on ${X}_2$ and calculated the type I error rate for the null causal effect and statistical power to detect the non-zero causal effect. The nominal level was set to 0.05. Details of data generation are shown in Supplemental Material and Methods S6.

#### Simulation study 2 on MRSL with random graphs

To validate the utility of the MRSL method for learning structures, we performed a simulation study for continuous and binary variables, respectively. IVs were generated from a binomial distribution $B\left(2,0.3\right)\kern0.1em$. Let $X$ denote the N × *d* matrix of *d* variables and *G* denote a N × *g* matrix of *g* IVs. For continuous variables, *d* phenotypes were generated from the following model:


$$ {X}_i={P}_{X_i}\beta + G\alpha +U+\varepsilon, $$

where ${P}_{X_i}$ represents the parent nodes of ${X}_i$, $\beta$ are the effects of ${P}_{X_i}$ on ${X}_i$ and generated from a uniform distribution, $\alpha$ are the effects of SNPs on phenotypes, *U* represents the unmeasured confounding among *d* phenotypes and $\varepsilon$ is the residual term following a normal distribution $N\left(0,1\right)\kern0.1em$. For binary variables, *d* variables are generated from the following model:


$$ \log it\left[P\left({X}_i=1\right)\right]={P}_{X_i}\beta + G\alpha +U. $$

We generated 10 000 independent individuals for each variable and 100 repeated datasets. Then, we generated summary data based on the above individual data. For continuous variables, summary statistics can be obtained by linear regressions of each phenotype on IVs. For binary variables, summary statistics can be obtained by logistic regressions of each phenotype on IVs. We generated random graphs with 5, 10 and 15 nodes. Considering the different complexity of the networks, we set the probability of each edge to be present in a graph as 0.2, 0.5 and 0.8. In practice, the magnitude of the effects may vary between traits. Thus, we considered $\beta$ follows a uniform distribution with four parameter settings: U(0,0.25), U(0.25,0.5), U(0.5,0.75) and U(0.75,1) for continuous variables, ORs U(1,1.5), U(1.5,2), U(2,2.5) and U(2.5,3) for binary variables. The IVs were assumed to be uncorrelated and subdivided into two categories: (1) ${g}_1$ SNPs that only predict one phenotype and (2) ${g}_2$ SNPs that predict all the phenotypes simultaneously. We vary the number of SNPs ${g}_1$ and ${g}_2$ with values of 5, 10, 20, 30, 40 and 50, respectively.

We compared our method with eight published methods: BIMMER [13], cGAUGE based on IVW, MR Egger and MR PRESSO [12], the HC algorithm incorporating genetic anchors [10] (based on genetic risk scores or the most significant SNP) and the MRPC algorithm [11] (based on genetic risks score or the most significant SNP). Details are shown in the Supplemental Material and Methods S7. We used two metrics to assess the accuracy of the topological order calculated by DFS: relative Spearman’s footrule and Kendall’s tau [22]. To assess the performance of the algorithm, we computed the mean of F1 score, recall, precision and computing time across 100 data sets with 10 000 individuals for each method. Recall (i.e. power or sensitivity) measures how many edges a method can recover from the true causal graph, whereas precision (i.e. 1-FDR) measures how many correct edges are recovered in the inferred graph. The F1 score is a combined index of recall and precision. Details of the calculation formula are shown in Supplemental Material and Methods S9 and S10.

#### Simulation study 3 on MRSL with fixed graphs

**Figure 3:**
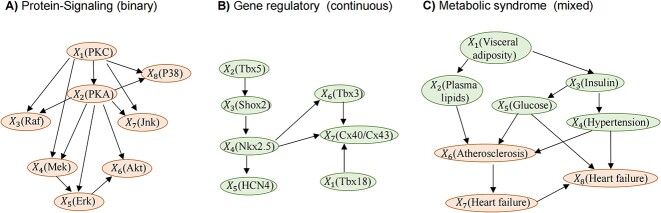
Diagrams for the practical examples in the simulation study 3.

To evaluate the performance of MRSL in practical application, we chose three representative examples (Figure 3A–C): (A) Protein-Signaling consists of eight proteins (binary variables: activate/inhibit) in a high-accuracy human primary T cell signaling causality map [23]. Eight proteins include PKC, PKA, Raf, Mek, Erk, Akt, Jnk and P38. (B) Gene regulatory consists of seven genes (continuous variables: gene expression) in SAN myocyte of the cardiac conduction system [24]. Seven genes include Tbx5, Shox2, Nkx2.5, HCN4, Tbx3, Cx40/Cx43 and Tbx18. (C) Metabolic syndrome (MetSyn) consists of eight MetSyn traits (mixed variables) in cardiovascular diseases [25], namely, visceral adiposity, plasma lipids, insulin, glucose, hypertension, atherosclerosis, myocardial infarction and heart failure. The data generation process, parameter settings and performance metrics of these three networks were similar to those in simulation study 2. Details of data generation are shown in Supplemental Material and Methods S8.

#### Simulation study 4 on MRSL with invalid IVs

We evaluated the performance of MRSL when there are invalid IVs, including correlated pleiotropic and weak IVs. We added several methods into our algorithm, namely, pleiotropy-robust MR methods (MR Egger [26], weighted median [27], mode-based method [28], MR mix [29] and MR raps [30]) and causal direction (CD) methods (CD Egger [31] and CD cML [32]) instead of IVW as bi-directional MR methods in MRSL. Among these methods, MR mix and CD cML were robust to correlated pleiotropy. For correlated pleiotropic IVs, we generate data by adding the direct effect from G to unmeasured confounders U, i.e.

$U=\sum \limits_i{\phi}_i{G}_i+{\varepsilon}_U$ , where ${\varepsilon}_U\sim N\left(0,1\right)$.

We set ${\phi}_i\sim U\left[-0.1,0.1\right]$ to represent correlated pleiotropy. For weak IVs, we set the variance of each phenotype explained by all the SNPs as 5%, 4%, 3% and 1%. We generated 20 and 50 IVs for each continuous and binary variable, respectively, and the proportion of invalid IVs were 0%, 10%, 30%, 50% and 80%. In the third step of MRSL, we chose the third sufficient separating set $AD{J}_{X_p\to{X}_q}$=V\{${X}_p$,${X}_q$ and ${S}^d$}. We also used the F1 score, recall and precision, as well as relative Spearman’s footrule and Kendall’s tau to evaluate the performance of MRSL.

### Applied example: causal network of 26 biomarkers and 44 ICD10-defined diseases in the UK Biobank

We applied MRSL to learn the network of 26 biomarkers and 44 ICD10-defined diseases using GWAS summary data in the UK Biobank. The UK Biobank is a prospective cohort study with deep genetic, physical and health data collected on more than 500 000 individuals (age range 40–69 years) across the UK from 2006 to 2010. The UK Biobank study was approved by the National Research Ethics Service Committee North West—Haydock, all participants provided informed written consent and all study procedures were performed in accordance with the World Medical Association Declaration of Helsinki ethical principles for medical research.

We use the GWAS summary statistics obtained specifically from GWAS with inverse rank normalized quantitative phenotypes. For MRSL, we first clumped the UK Biobank summary statistics to *P* < 5 × 10^−8^ for 26 biomarkers and 44 diseases, with *r*^2^ < 0.001 and distance 10 000 kilobases using the European reference panel in mr-base (https://www.mrbase.org/). To avoid selection bias, we chose IVs in the male population and used the summarized statistics in the female population. We conducted bi-directional MR to obtain a marginal causal graph. For pairwise MR analysis, we selected the SNPs associated with the exposure but not associated with other variables (except exposure and outcome) as IVs. For instance, when performing MR ${X}_3\sim{X}_1$ based on a network of four variables ${X}_1,{X}_2,{X}_3,{X}_4$, SNPs associated with ${X}_1$ but not associated with ${X}_2,{X}_4$ are selected as IVs. Next, we performed MVMR using three adjustment strategies to obtain the true graph. We selected SNPs associated with at least one phenotype of exposure and the variables in the sufficient separating set as IVs. For example, when performing the MVMR ${X}_3\sim{X}_1+{X}_4$, SNPs associated with at least one of ${X}_1$ and ${X}_4$ but not associated with ${X}_2$ are selected as IVs. For each MVMR, we also filtered out the SNPs in linkage disequilibrium (*r*^2^ < 0.001).

## RESULTS

### Simulations

#### Simulation study 1 on IVs selection in MVMR

**Figure 4:**
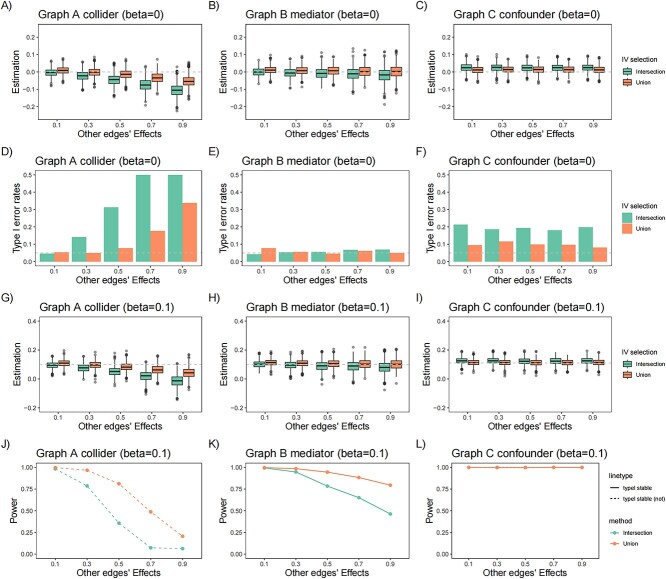
Simulation results of MVMR with different IVs in simulation study 1. (**A**–**C**) Causal effect estimation of ${X}_1$ on ${X}_2$ when causal effect $\beta =0$; (**D**–**F**) Type I error rates of causal effect estimation of ${X}_1$ on ${X}_2$ when causal effect $\beta =0$; (**G**–**I**) Causal effect estimation of ${X}_1$ on ${X}_2$ when causal effect $\beta =0.1$; (**J**–**L**) Statistical power of causal effect estimation of ${X}_1$ on ${X}_2$ when causal effect $\beta =0.1$. The *x*-axis represents the other edges’ effect (${\alpha}_1$ and ${\alpha}_2$ in Figure 2). For the results of power, dashed lines denote the power for methods that do not keep the type I error level. Solid lines denote the power for methods that keep the type I error level.

First, we conducted a simulation study to evaluate the performance of MVMR in estimating the direct causal effect of an exposure (${X}_1$) on an outcome (${X}_2$) when adjusting for a collider (${X}_3$), a mediator (${X}_4$) or a measured confounder (${X}_5$), respectively. Figure 4 shows the results of MVMR when there are 100 IVs. The first column (A, D, G and J) shows the results of causal effects estimation of MVMR adjusting for a collider. The two candidate IVs are biased, and the bias when using union IVs was smaller than intersection IVs. Our three kinds of sufficient separating sets exclude colliders due to their large biases. Therefore, we selected the optimal IVs based on the performance of MVMR adjusting for mediators and measured confounders. When adjusting for mediators, the causal effect estimation was unbiased when using union IVs, while it showed a slight downward bias when using intersection IVs. Their type I error rates were stable around 0.05, and the power of causal effect estimation when using union IVs was higher than intersection IVs. When adjusting for measured confounders, the causal effect estimation was unbiased when using union IVs, while it showed a slight upward bias when using intersection IVs. Their powers were high up to 1, and the type I error rates of causal estimation when using intersection IVs were more inflated than union IVs. The simulation results of using 6, 20 and 60 IVs are shown in Figures S1–S4. In practice, practitioners are not always certain about the roles of the adjusting variables. So, considering the above three graphs together, ${G}_1+{G}_2+{G}_3$ is the best choice of IVs when performing MVMR.

#### Simulation study 2 on MRSL with random graphs

**Figure 5:**
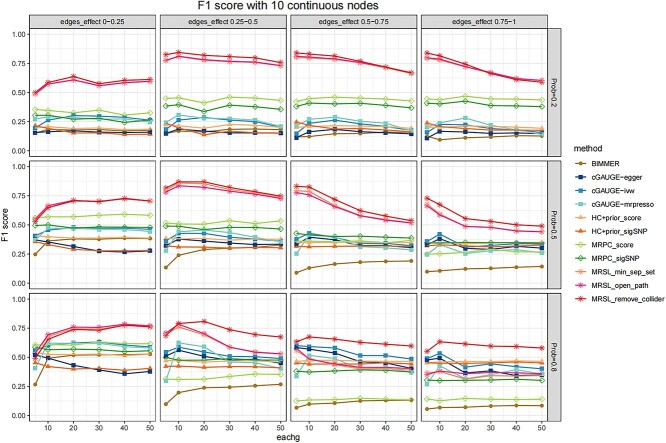
F1 score with 10 continuous nodes in simulation study 2. The *x*-axis represents the number of IVs. Considering the different complexities of the network, we set the probability of each edge to be present in a graph as 0.2, 0.5 and 0.8. The effects of any two traits $\beta$ followed a uniform distribution with four parameter settings: U(0,0.25), U(0.25,0.5), U(0.5,0.75) and U(0.75,1) for continuous nodes. MRSL_min_sep_set indicates the MRSL adjusting for minimal sufficient adjustment set and all the mediators; MRSL_open_path indicates the MRSL adjusting for all the nodes on the open paths; MRSL_remove_collider indicates the MRSL adjusting for V\{${X}_p$,${X}_q$ and${S}^d$}.

**Figure 6:**
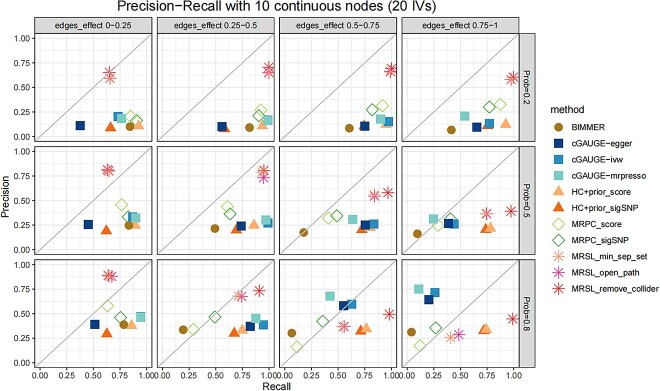
Precision and recall with 10 continuous nodes when there are 20 IVs in simulation study 2. Considering the different complexities of the network, we set the probability of each edge to be present in a graph as 0.2, 0.5 and 0.8. The effects of any two traits $\beta$ followed a uniform distribution with four parameter settings: U(0,0.25), U(0.25,0.5), U(0.5,0.75) and U(0.75,1) for continuous nodes.

**Figure 7:**
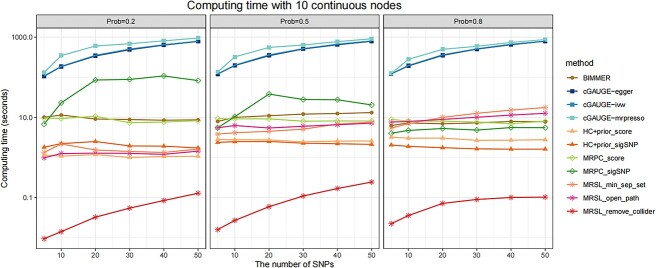
Computing time with network of 10 continuous nodes in simulation study 2 (seconds). Considering the different complexities of the network, we set the probability of each edge to be present in a graph as 0.2, 0.5 and 0.8. The effects of any two traits $\beta$ followed as uniform distribution U(0.25,0.5) for continuous nodes.

We conducted a simulation study for continuous and binary phenotypes to learn the structures of random graphs, and the results of 10 continuous nodes are shown in Figure 5–7. Figure 5 demonstrates the F1 score with different edges’ effects and network complexity. Figure 6 shows the mean of precision and recall when there are 20 IVs. Results of precision and recall when there are 5, 10, 30, 40 and 50 IVs are shown in Figure S5–S8. When the network is simple (prob = 0.2), the F1 score of MRSL was highest, and the performance of the three adjustment categories was similar. With greater network complexity, the F1 score of MRSL when adjusting for all nodes on the open paths and minimum separated set decrease, MRSL still had the highest F1 score when adjusting for V\{${X}_p$,${X}_q$,${S}^d$ and U}. The recall of the former was smaller than the latter as the edges’ effects and the complexity of the graph increased. When the edges’ effects were small, the F1 score of MRSL increased with the number of IVs increased. When the edges’ effects are large, the F1 score of MRSL decreased as the number of IVs increased owing to the reduced precision. This may be because, in simulation study 1, the increasing number of IVs was associated with greater inflation of the type I error rate of ${G}_1+{G}_2+{G}_3$, leading to increased false-negative rates. Besides, the power of causal estimation using MVMR decreased with increased effects of other edges. Additionally, the number of adjustment variables increases with network complexity, reducing the accuracy of causal estimation using MVMR. Figure 7 shows the computing time of MRSL and the other eight methods using 5, 20 and 50 IVs. MRSL had the fastest computing time among these methods. To ensure the fair and accurate of comparison, the computing time of all the methods includes the time of generating the marginal causal graph. The computing time of all the methods with 10, 30 and 40 IVs are listed in Table S1. The results of MRSL with 10 binary nodes are similar to those with continuous nodes (Figures S9–S13 and Table S2). The F1 score of MRSL reduced with increased nodes in the network, especially when the network is complex. MRSL had lower power to detect ORs for binary variables than the beta coefficient for continuous variables. This could be due to the collapsibility of logistic regression coefficients and the effect estimates from multivariable regression model. This phenomenon is shown in the Supplemental Materials and Methods (Figures S9 and S19). The results of 5 and 15 nodes are shown in Figures S14–S33 and Tables S3–S6.

We used two metrics to quantify the accuracy of topological sorting calculated by the DFS algorithm. The relative Spearman’s footrule was about 0.05, which means changing the first rank to the second rank requires 5% element-wise displacement from the identity permutation. A relative Spearman’s footrule of 0.05 indicates a small probability event, i.e. the element-wise displacement from the identity permutation is small. Kendall’s tau was far from 0 and was larger than 0.8, indicating the strong concordant pairs between the two lists. We believe DFS can be used to specify the topological correctly.

#### Simulation study 3 on MRSL with fixed graphs

**Figure 8:**
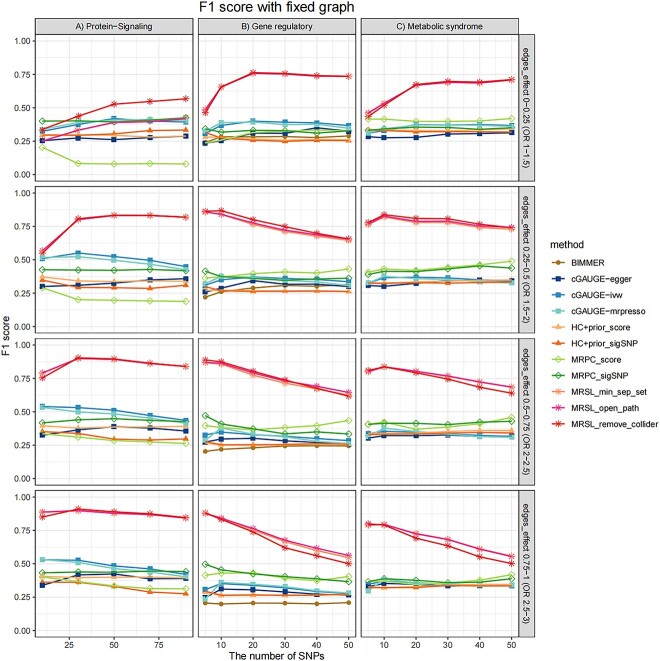
F1 score of MRSL when learning the structure of Gene regulatory, Protein-Signaling and Metabolic syndrome in simulation study 3. Considering the different network complexities, we set the probability of each edge to be present in a graph as 0.2, 0.5 and 0.8. The effects of any two traits $\beta$ followed a uniform distribution with four parameter settings: U(0,0.25), U(0.25,0.5), U(0.5,0.75) and U(0.75,1) for continuous nodes.

To evaluate the performance of MRSL in practical application, we chose three fixed networks, which are representative examples in practice, including Gene regulatory, Protein-Signaling and Metabolic syndrome, with continuous, binary and mixed nodes, respectively. Figure 8 shows the F1 scores of MRSL and eight methods when learning three networks. MRSL had the best performance, while Protein-Signaling (binary) and Gene regulatory (continuous) performed similarly to simulation study 2. For Metabolic syndrome (mixed), the F1 score was between that of Protein-Signaling and Gene regulatory. The F1 score of the continuous variable network (Gene regulatory) is higher than that of the binary variable network (Protein-Signaling). When the edges’ effects are small, MRSL has higher F1 scores than other methods as the number of SNPs increasing in the of Protein-Signaling network. Thus, in a binary network, when the causal effects between edges are small, MRSL needs more SNPs than in a continuous network to have enough power to outperform the other eight methods. When the edges’ effects are larger, MRSL had a slightly larger F1 score when adjusting for all nodes on the open paths and minimum separated set than when adjusting for V\{${X}_p$,${X}_q$,${S}^d$ and U} because of higher precision. The precision and recall are shown in Figures S34–S45.

#### Simulation study 4 on MRSL with invalid IVs

**Figure 9:**
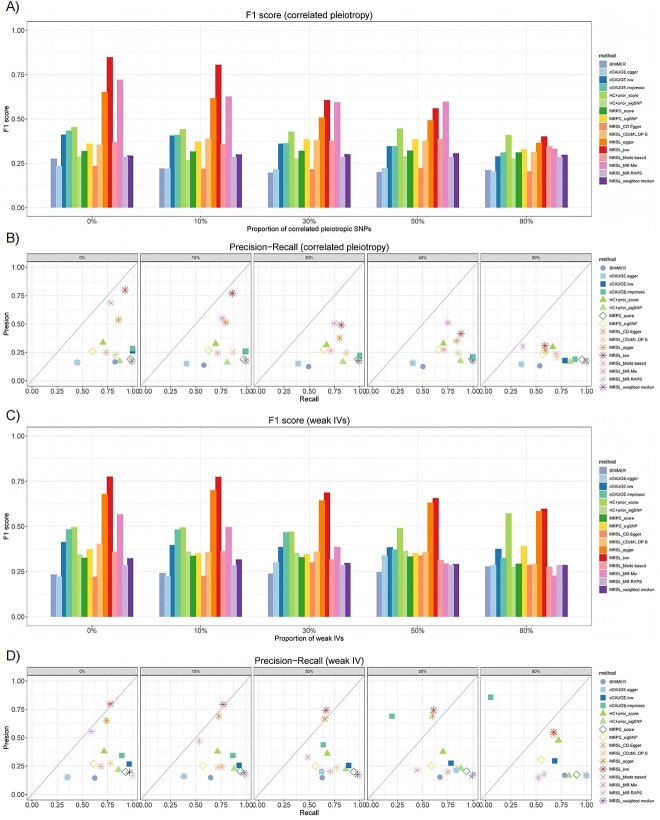
F1 score, precision and recall of MRSL when there are invalid IVs (continuous variables). (**A**) F1 score of MRSL and eight other methods when there are correlated pleiotropic IVs; (**B**) precision–recall of MRSL and eight other methods when there are correlated pleiotropic IVs; (**C**) F1 score of MRSL and eight other methods when there are weak IVs; and (**D**) precision–recall of MRSL and eight methods when there are weak IVs.

We conducted sensitivity analyses to evaluate the performance of MRSL when IVs are invalid, including correlated pleiotropic and weak IVs. In step 1 of MRSL, we used pleiotropy-robust and weak IV–robust MR methods instead of IVW to perform bi-directional MR. In step 3, we chose the third sufficient separating set $AD{J}_{X_p\to{X}_q}$=V\{${X}_p$,${X}_q$ and ${S}^d$}. We considered the different proportions of invalid IVs (Figure 9, Figure S46 and Tables S7 and S8). For correlated pleiotropic IVs, MRSL based on IVW performed best when there were less than 50% invalid IVs. Additionally, MRSL based on MR mix and MR Egger outperformed other methods. When there were 80% invalid IVs, the performance of MRSL was similar to that of HC adding prior genetic risk score. For weak IVs, MRSL based on IVW and MR Egger performed best when there were less than 50% invalid IVs. Broadly, correlated pleiotropic IVs had a larger influence on the F1 score of MRSL than weak IVs. Nevertheless, MRSL outperformed other methods. For algorithm convergence, as the proportion of correlated pleiotropic and weak IVs increases, the estimated causal graph deviates from the true causal graph. However, the magnitude of deviation was minimal across all methods as long as when there were less than 50% invalid IVs.

### Applied example: causal network of 26 biomarkers and 44 ICD10-defined diseases in UK Biobank

**Figure 10:**
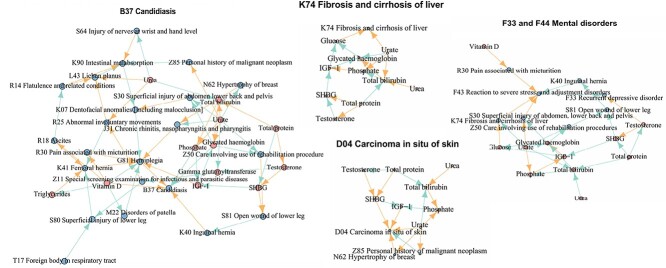
Mediation pathways in network of 26 biomarkers and 44 diseases from UK Biobank.

We applied MRSL to learn the network of 44 diseases with ICD-10 codes and 26 biomarkers using GWAS summary data in the UK Biobank. The list of these 70 traits is shown in Table S9. Figure S48A shows the marginal causal graph, resulting in 70 nodes and 388 edges. Figure S45Bshows the conditional causal graph obtained by MVMR adjusting for V\{${X}_p$,${X}_q$, ${S}^d$ and U}, resulting in 69 nodes and 192 edges. This result was obtained by removing 196 direct edges induced by mediation pathways after Bonferroni correction. All the edges are listed in Table S10. Figure 10 shows the causal mediation pathways from biomarkers for each disease. Vitamin D, total protein, urate and urea were the main causes for nearly all the mediation pathways of diseases [33–35].

Most of the causal links were expected and have a clear interpretation of biological pathways or have been confirmed by experiments. For instance, B37 Candidiasis, Vitamin D [36], K40 Inguinal hernia [37] and G81 Hemiplegia [38] are direct risk factors; Phosphate [39] and Glycated hemoglobin [40] are direct protective factors. For F33 Recurrent depressive disorder, testosterone has a positive effect on F33 [41]. Other biomarkers affect F33 through F43 reaction to severe stress and adjustment disorders. IGF-1 [42] directly influences the risk of D04 Carcinoma *in situ* of the skin. Vitamin D is directly protective against G81 Hemiplegia with a protective effect [43]. Glucose [44] and Urate [45] are risk factors for K74 Fibrosis and liver cirrhosis. Biomarkers have causal effects on K90 Intestinal malabsorption through R14 Flatulence [46] and related conditions and L43 Lichen planus [47].

Several novel causal links were found and supported by clinical case reports or observational studies. For example, Urate [48], T17 Foreign body in the respiratory tract [49, 50], F31 Bipolar affective disorder [51] and K41 Femoral hernia [52] have negative causal effects on C16 the Malignant neoplasm of the stomach. Urate [53] positively affects the risk of Carcinoma *in situ* of the skin. IGF-1 [54] and K07 Dentofacial anomalies, including malocclusion, directly increase the risk of G81 Hemiplegia. For H60 Otitis externa, Glycated hemoglobin [55] is protective, whereas J03 Acute tonsillitis is a risk factor. Moreover, IGF-1 [56], HDL cholesterol [57], total protein [58] and total bilirubin [59] positively affect J03 Acute tonsillitis. Urate [60] is a risk factor for K12 Stomatitis and related lesions. Urate negatively affects the risk of M81 Osteoporosis without pathological fracture [61], whereas R25 Abnormal involuntary movements is a risk factor [62].

## DISCUSSION

This work presented a causal network pruning algorithm called MRSL based on MVMR for structural learning. Our method is flexible as it requires only summarized genetic data. Besides, MRSL relaxes the causal sufficiency assumption, can be implemented with fast computing speed and outputs a conditional causal graph with directed causal effects. The application to 26 biomarkers and 44 ICD10-defined diseases in UK Biobank covers many expected causal links with biological interpretations and several new links supported by clinical case reports or previous observational literature.

The core of MRSL is MR analysis, and the performance of MRSL depends on that of MR. The first point we need to focus on is the selection of IVs. For the bi-directional MR, an option is the SNPs only associated with the exposure but not associated with other variables (except exposure and outcome) in the network. To some extent, this can block nearly all pleiotropic pathways. For MVMR, we first conducted a simulation study to choose the most valid IVs. We considered only valid IVs, which optimizes MVMR when adjusting for the collider, mediator and confounder simultaneously. The results of simulation 1 indicate ${G}_1+{G}_2+{G}_3$ is the best choice, congruent with previous literature [16, 17, 63]. Our simulation also reveals that when the number of IVs is above 20, there is enough power to detect a causal effect (0.1) using ${G}_1+{G}_2+{G}_3$. When adjusting for confounders in MVMR, statistical power using intersection IVs is higher than union IVs when there are few IVs (6 and 20 IVs in Figure S4) because the causal estimation using intersection IVs is biased toward >0.1 (Figure S3). Therefore, from the perspective of unbiased causal effect estimation and hypothesis testing, the union IV sets ${G}_1+{G}_2+{G}_3$ are optimal for MVMR in any scenario. It is crucial to have as many IVs as possible, meaning including genetic variables associated with at least one exposure. Removing instruments that are only strongly associated with one exposure will lead to a loss of precision in the estimation or other potential biases. We only focused on the effect of a particular exposure on the outcome using MVMR each time. Thus, we only forced a positive association concerning the exposure of interest [63]. This does not influence our results, but this changes the sign of the association based on the adjustment variables. We used univariable and multivariable IVW as the main methods. MRSL can be extended to use other UVMR methods, such as pleiotropy-robust methods (e.g. MR-Egger [26], the weighted median method [27], the mode-based estimate method [28], MR-RAPS [30] and contamination mixture method [29]) and MVMR methods (e.g. MVMR-Egger, MVMR-Robust, MVMR-Median, MVMR-Lasso [63]) instead of IVW. However, combining these methods in MRSL is time-consuming and may cause a loss of precision due to the low accuracy of these methods.

Our algorithm converges even when the IVs are invalid or weak, but may converge to an incorrect causal graph. Invalid or weak IVs may wrongly remove true edges and cannot remove spurious ones. The former has a greater influence on the algorithm convergence than the latter because the second step of our algorithm only removes spurious but cannot recover the edges. If IVs are not robustly associated with the exposure, estimates will be biased toward the null in a two-sample MR. Thus, weak IVs may not have enough power to discover the causal relationships in the marginal causal graph. Consequently, these relationships cannot be recovered in subsequent steps. Many related factors should be included in the network to reduce the influence of independent horizontal pleiotropy, and all the possible pleiotropy pathways can be blocked by MVMR. Correlated pleiotropy is difficult to test, but valid IVs can be selected by removing outliers by MR PRESSO, MR Radial, etc.

The marginal causal graph 𝒢_M_ can be obtained through published pairwise bi-directional MR [64], causal direction methods (MR Steiger, CD ratio, CD Egger [31] and CDcML [32]) or by directly summarizing the results from published MR studies or other causal evidence. In the application, we conducted bi-directional MR to obtain a marginal causal graph and selected valid IVs to ensure the accuracy of the graph. This step is crucial to ensure that a causal relationship between two nodes in a true causal graph mandates a corresponding connection in the marginal causal graph. Missing true edges in the margin causal graph cannot be recovered in the subsequent MRSL analysis. This phenomenon may also induce that MRSL converges to an incorrect causal graph. In the second step of MRSL, we presented three strategies for adjusting variables in MVMR with the complement of graph theory in causal inference. Because MR overcomes unobserved confounding, we excluded U in the three sets of adjustment variables.

Another aspect we pay attention to is whether these three sets of adjusting variables are the same in the marginal causal graph and the true causal graph. In other words, we had two questions: does adjusting these variables in the marginal causal graph unlock the blocked pathways in the true causal graph or incompletely block the mediation pathways in the true causal graph? We propose Lemmas 1 and 2 and Theorem 1 for these two questions. The first way is adjusting for all nodes on the open paths in the marginal causal graph, which blocks all open paths between two variables, including mediation and confounding pathways. This adjustment set doesn’t include the spurious colliders in the marginal causal graph. For the second way, a minimal separating set may include spurious colliders at the cost of including other confounders or mediators to ensure the separation of two variables. This blocks the pathways in the true causal graph, as well as those in the marginal causal graph, including spurious pathways. The third adjustment set is the most conservative and, as is, adjusts for all the variables, excluding colliders. These particular colliders must have direct edges on the two variables of interest. In summary, the second step of MRSL removes extra edges in the marginal causal graph and obtains a conditional causal graph.

Combining graph theory and MVMR is a unique property of our algorithm, and we utilize this novel property in causal discovery to improve precision and recall. Our method can be easily implemented using GWAS summary data, which are publicly available for most phenotypes with the emergence of multiple GWAS studies with large sample sizes. Published MR-based algorithms, such as cGAUGE, require individual-level data that are not as easily available and are time-consuming. BIMMER is implemented based on the complex inverse sparse regression and obtains an approximate estimation of the DCE matrix; this requires time roughly 𝒪(*κ d*^4^) for *d* phenotypes. For MRSL, MVMR is performed to obtain a conditional causal graph, and this requires at most (*κ d*^2^) for *d* phenotypes. In simulation studies 2 and 3, we found that the computing time of MRSL is only around 1/100 of BIMMER and 1/1000 of cGAUGE, respectively. MRSL has a 2-fold higher F1 score than the other eight methods when the network is simple. Additionally, MRSL outputs the unbiased direct effect of each pair of variables. Moreover, MRSL can be applied to the structure with feedback loops between any two variables, because our main MR IVW method can robustly deal with bi-directional causal relationships between two variables [23]. Similar to MR analysis, GWAS summary data of *d* phenotypes should come from a homogenous population. Another limitation in our application is that we only conducted the basic linear or logistic regression models using PLINK2, which is not the best tool to run a GWAS for imputed data, especially for the complex statistical model. We also need to address other issues in the future, such as measurement error, selection bias and missing data.

In conclusion, we proposed a novel algorithm that combines graph theory and MR into causal discovery to learn the conditional causal graph. We look forward to offering constructive suggestions for disease diagnosis and applying our method beyond the scope considered here.

**Key Points**

- Recently, Mendelian randomization (MR) studies have provided abundant marginal causal relationships of traits. We propose a causal network pruning algorithm, MRSL, which combines the graph theory with multivariable MR to learn the conditional causal structure using only genome-wide association analyses (GWAS) summary statistics.
- MRSL utilizes topological sorting to improve the precision of structure learning. It proposes MR-separation instead of d-separation and three candidates of sufficient separating set for MR-separation.
- The results of simulations revealed that MRSL had up to 2-fold higher F1 score and 100 times faster computing time than other eight competitive methods.
- We applied MRSL to 26 biomarkers and 44 ICD10-defined diseases using GWAS summary data from UK Biobank.

## Supplementary Material

Supplemental_Material_and_Methods-revised-v1_bbae086

## Data Availability

The GWAS summary data in UK Biobank are publicly available at http://www.nealelab.is/uk-biobank. All the analyses in our article were implemented by R software. MRSL can be implemented by https://github.com/hhoulei/MRSL. All the codes for simulation and toy example are uploaded in https://github.com/hhoulei/MRSL_Simul. BIMMER was implemented using R packages *bimmer*. MRPC was implemented using R packages *MRPC*. The HC algorithm was implemented using R packages *bnlearn*. cGAUGE was implemented using functions in https://github.com/david-dd-amar/cGAUGE and R packages *MendelianRandomization*, *MRPRESSO*. All the networks were plotted using R packages *igraph*.

## Author Biographies

**Lei Hou** received her PhD degree from Shandong University, China, in 2023. She is working as a post-doctor in Peking University, China. His currently research interests include causal network analysis, multi-omics analysis and unmeasured confounding controlling in causal inference.

**Zhi Geng** is a professor at School of Mathematics and Statistics at Beijing Technology and Business University, China. His main research interest is causal inference.

**Zhongshang Yuan** is a professor at School of Public Health at Shandong University, China. His main research interest is the integration and statistical theoretical methods of omics data analysis.

**Xu Shi** is an assistant professor at School of Public Health at University of Michigan. She is interested in developing novel statistical methods that provide insights from high volume and high variability administrative healthcare data such as electronic health records (EHR) and claims data.

**Chuan Wang** is an attending physician at Endocrinology Department of Qilu Hospital of Shandong University. His clinical expertise in the diagnosis and treatment of various common endocrine disorders, including diabetes, hypertension, hyperlipidemia, and thyroid diseases.

**Feng Chen** is a professor at School of Public Health at Nanjing Medical University. His research interests include non-independent data, high-dimensional biomedical data, and statistical theory and methods in clinical research.

**Hongkai Li** is a professor at School of Public Health at Shandong University, China. His main research interests include methods for controlling unknown confounding, data integration under causal inference frameworks, mediation analysis, methods for controlling measurement error and selection bias, and causal inference problems within a time series framework.

**Fuzhong Xue** is a professor at School of Public Health at Shandong University, China. His main research interests include the theoretical and methodological study of health and medical big data analysis, as well as the theoretical methods for causal inference in big data.
