# Supplementary material for: MRSL: a causal network pruning algorithm based on GWAS summary data
Source: Brief Bioinform. 2024 Mar 14;25(2):bbae086. doi: 10.1093/bib/bbae086 (PMC10940843; doi:10.1093/bib/bbae086)
Supplement: Supplemental_Material_and_Methods-revised-v1_bbae086 [file supplemental_material_and_methods-revised-v1_bbae086.docx]

Supplemental Material and Methods

[Supplemental Material and Methods 1](#_Toc156989110)

[S1 Univariable MR (UVMR) and Multivariable MR (MVMR) 4](#_Toc156989111)

[S2 Proof of Lemma 1 5](#_Toc156989112)

[S3 Proof of Lemma 2 5](#_Toc156989113)

[S4 Proof of Theorem 1 5](#_Toc156989114)

[S5 Topological sorting and DFS algorithm 6](#_Toc156989115)

[S6 Data generation of Simulation Study 1 7](#_Toc156989116)

[S7 Details of the 8 competing methods were applied in the simulation 10](#_Toc156989117)

[S8 Data generation of Simulation Study 3 11](#_Toc156989118)

[S9 Calculation formula of F1score, recall and precision 13](#_Toc156989119)

[S10 Calculation formula of Relative spearman's footrule and Kendall’s tau 13](#_Toc156989120)

[Figure S1. Estimation of causal effect using MVMR in simulation study 1 (causal effect=0) 14](#_Toc156989121)

[Figure S2. Type I error rate of causal effect using MVMR in simulation study 1 (causal effect=0) 15](#_Toc156989122)

[Figure S3. Estimation of causal effect using MVMR in simulation study 1 (causal effect=0.1) 16](#_Toc156989123)

[Figure S4. Statistical power of causal effect using MVMR in simulation study 1 (causal effect=0.1) 17](#_Toc156989124)

[Figure S5. Precision−Recall with 10 continuous nodes when edges' effect between 0−0.25 in simulation study 2 18](#_Toc156989125)

[Figure S6. Precision−Recall with 10 continuous nodes when edges' effect between 0.25−0.5 in simulation study 2 19](#_Toc156989126)

[Figure S7. Precision−Recall with 10 continuous nodes when edges' effect between 0.5−0.75 in simulation study 2 20](#_Toc156989127)

[Figure S8. Precision−Recall with 10 continuous nodes when edges' effect between 0.75−1 in simulation study 2 21](#_Toc156989128)

[Figure S9. F1 score with 10 binary nodes 22](#_Toc156989129)

[Figure S10. Precision−Recall with 10 binary nodes when OR between 1−1.5 in simulation study 2 23](#_Toc156989130)

[Figure S11. Precision−Recall with 10 binary nodes when OR between 1.5−2 in simulation study 2 24](#_Toc156989131)

[Figure S12. Precision−Recall with 10 binary nodes when OR between 2−2.5 in simulation study 2 25](#_Toc156989132)

[Figure S13. Precision−Recall with 10 binary nodes when OR between 2.5−3 in simulation study 2 26](#_Toc156989133)

[Figure S14. F1 score with 5 continuous nodes in simulation study 2 27](#_Toc156989134)

[Figure S15. Precision−Recall with 5 continuous nodes when edges' effect between 0−0.25 in simulation study 2 28](#_Toc156989135)

[Figure S16. Precision−Recall with 5 continuous nodes when edges' effect between 0.25−0.5 in simulation study 2 29](#_Toc156989136)

[Figure S17. Precision−Recall with 5 continuous nodes when edges' effect between 0.5−0.75 in simulation study 2 30](#_Toc156989137)

[Figure S18. Precision−Recall with 5 continuous nodes when edges' effect between 0.75−1 in simulation study 2 31](#_Toc156989138)

[Figure S19. F1 score with 5 binary nodes in simulation study 2 32](#_Toc156989139)

[Figure S20. Precision−Recall with 5 binary nodes when OR between 1−1.5 in simulation study 2 33](#_Toc156989140)

[Figure S21. Precision−Recall with 5 binary nodes when OR between 1.5−2 in simulation study 2 34](#_Toc156989141)

[Figure S22. Precision−Recall with 5 binary nodes when OR between 2−2.5 in simulation study 2 35](#_Toc156989142)

[Figure S23. Precision−Recall with 5 binary nodes when OR between 2.5−3 in simulation study 2 36](#_Toc156989143)

[Figure S24. F1 score with 15 continuous nodes in simulation study 2 37](#_Toc156989144)

[Figure S25. Precision−Recall with 15 continuous nodes when edges' effect between 0−0.25 in simulation study 2 38](#_Toc156989145)

[Figure S26. Precision−Recall with 15 continuous nodes when edges' effect between 0.25−0.5 in simulation study 2 39](#_Toc156989146)

[Figure S27. Precision−Recall with 15 continuous nodes when edges' effect between 0.5−0.75 in simulation study 2 40](#_Toc156989147)

[Figure S28. Precision−Recall with 15 continuous nodes when edges' effect between 0.75−1 in simulation study 2 41](#_Toc156989148)

[Figure S29. F1 score with 15 binary nodes in simulation study 2 42](#_Toc156989149)

[Figure S30. Precision−Recall with 15 binary nodes when OR between 1−1.5 in simulation study 2 43](#_Toc156989150)

[Figure S31. Precision−Recall with 15 binary nodes when OR between 1.5−2 in simulation study 2 44](#_Toc156989151)

[Figure S32. Precision−Recall with 15 binary nodes when OR between 2−2.5 in simulation study 2 45](#_Toc156989152)

[Figure S33. Precision−Recall with 15 binary nodes when OR between 2.5−3 in simulation study 2 46](#_Toc156989153)

[Figure S34. Precision−Recall with Gene regulatory graph when edges' effect between 0−0.25 in simulation study 3 47](#_Toc156989154)

[Figure S35. Precision−Recall with Gene regulatory graph when edges' effect between 0.25−0.5 in simulation study 3 48](#_Toc156989155)

[Figure S36. Precision−Recall with Gene regulatory graph when edges' effect between 0.5−0.75 in simulation study 3 49](#_Toc156989156)

[Figure S37. Precision−Recall with Gene regulatory graph when edges' effect between 0.75−1 in simulation study 3 50](#_Toc156989157)

[Figure S38. Precision−Recall with Protein-Signaling graph when OR between 1−1.5 in simulation study 3 51](#_Toc156989158)

[Figure S39. Precision−Recall with Protein-Signaling graph when OR between 1.5−2 in simulation study 3 52](#_Toc156989159)

[Figure S40. Precision−Recall with Protein-Signaling graph when OR between 2−2.5 in simulation study 3 53](#_Toc156989160)

[Figure S41. Precision−Recall with Protein-Signaling graph when OR between 2.5−3 in simulation study 3 54](#_Toc156989161)

[Figure S42. Precision−Recall with Metabolic syndrome graph when edges' effect between 0−0.25 55](#_Toc156989162)

[Figure S43. Precision−Recall with Metabolic syndrome graph when edges' effect between 0.25−0.5 56](#_Toc156989163)

[Figure S44. Precision−Recall with Metabolic syndrome graph when edges' effect between 0.5−0.75 57](#_Toc156989164)

[Figure S45. Precision−Recall with Metabolic syndrome graph when edges' effect between 0.75−1 58](#_Toc156989165)

[Figure S46. F1score, precision and recall in simulation study 4 (binary variable) 59](#_Toc156989166)

[Figure S47. Example for MRSL with and without topological sorting 60](#_Toc156989167)

[Figure S48. Marginal and Conditional causal graph in Applied example 2 61](#_Toc156989168)

[Table S1. Computing time with network of 10 continuous nodes in simulation study 2 (seconds). 62](#_Toc156989169)

[Table S2. Computing time with network of 10 binary nodes in simulation study 2 (seconds). 65](#_Toc156989170)

[Table S3. Computing time with network of 5 continuous nodes in simulation study 2 (seconds). 69](#_Toc156989171)

[Table S4. Computing time with network of 5 binary nodes in simulation study 2 (seconds). 72](#_Toc156989172)

[Table S5. Computing time with network of 15 continuous nodes in simulation study 2 (seconds). 75](#_Toc156989173)

[Table S6. Computing time with network of 15 binary nodes in simulation study 2 (seconds). 78](#_Toc156989174)

[Table S7. Relative spearman's footrule and Kendall’s tau for estimated topological sorting by DFS algorithm when IVs are invalid (continuous variables). 81](#_Toc156989175)

[Table S8. Relative spearman's footrule and Kendall’s tau for estimated topological sorting by DFS algorithm when IVs are invalid (binary variables). 82](#_Toc156989176)

[Table S9. 44 diseases and 26 biomarkers in the applied example 2. 83](#_Toc156989177)

[Table S10. Relationships in the Figure S48 86](#_Toc156989178)

# S1 Univariable MR (UVMR) and Multivariable MR (MVMR)


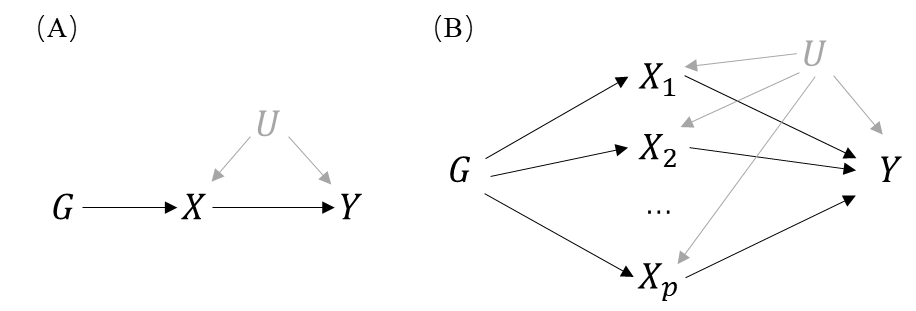


Figure S1. (A) Univariable MR (UVMR); (B) Multivariable MR (MVMR)

Univariable MR (UVMR) is the traditional MR analysis, is a form of instrumental variable (IV) analysis that uses genetic variants as instruments to obtain estimates of the causal effect of an exposure (X) on an outcome (Y) (Figure S1 (A)). It requires three assumptions:①Relevance: IVs are strongly associated with the exposure; ②Exchangeability: IVs are independent with confounders among the exposure and outcome; ③Exclusion restriction: IVs affect the outcome only through the exposure. The causal effect ($\beta_{XY}$) of *X* on *Y* can be estimated by IVW, that is, weighted linear regression:

$$\hat{\beta}_{Y_{j}}=\beta_{XY}\hat{\beta}_{X_{j}}+\varepsilon_{Y_{j}},\varepsilon_{Y_{j}}\sim N(0,\sigma_{Y_{j}}^{2})$$

This has recently been extended to estimate the independent direct effects of multiple, potentially related exposures through multivariable MR (MVMR) (Figure S2 (B)). A key feature of MVMR is that it estimates the effect of each exposure included in the estimation on the outcome conditional on the other exposures included in the model, that is, the direct effect of each exposure. MVMR also requires three assumptions: ①Relevance: IVs are strongly associated with at least one of multiple exposures; ②Exchangeability: IVs are independent with confounders among the exposures and outcome; ③Exclusion restriction: IVs affect the outcome only through the exposures. The causal effect ($\beta_{X_{i}Y}$) of each exposure $X_{i}$ on *Y* can be estimated by MV-IVW, that is, multivariate weighted linear regression:

$$\hat{\beta}_{Y_{j}}=\beta_{X_{1}Y}\hat{\beta}_{X_{1j}}+\beta_{X_{2}Y}\hat{\beta}_{X_{2j}}+...+\beta_{X_{p}Y}\hat{\beta}_{X_{pj}}+\varepsilon_{Y_{j}},\varepsilon_{Y_{j}}\sim N(0,\sigma_{Y_{j}}^{2})$$

$\beta_{X_{1}Y}$ can be estimated by the likelihood method or generalized least squares method, etc. And it can be tested by likelihood-ratio test or t-test and Wald test, etc. In this manuscript, we use the generalized least squares method and t-test.

# S2 Proof of Lemma 1

**Lemma 1.** For the true causal graph 𝒢 and the marginal causal graph 𝒢_M_, E_𝒢_⊆E_𝒢M_ and S_𝒢_⊆S_𝒢M_.

**Proof**: If $X_{p}$ has a causal relationship with $X_{q}$ in the true causal graph 𝒢, whether a direct or an indirect causal relationship, there exists an edge $X_{p}\to X_{q}$ in the marginal causal graph 𝒢_M_. There is no edge linking $X_{p}$ and $X_{q}$ in 𝒢_M_ if they are not causally related in 𝒢. Thus the collider in 𝒢 is also a collider in 𝒢_M_ but the extra edge may induce spurious collider in 𝒢_M_. Taking the edge $X_{1}\to X_{2}\to X_{3}$ as an example, the marginal causal graph 𝒢_M_ will add an extra edge $X_{1}\to X_{3}$. This extra edge induces a spurious collider $X_{3}$ in 𝒢_M_, that is, $X_{3}$ is not a collider in the graph 𝒢 but is a collider in 𝒢_M_ ($X_{1}\to X_{3}\leftarrow X_{2}$). ∎

# S3 Proof of Lemma 2

**Lemma 2 (Topological sorting invariance).** The topological sorting of the true causal graph 𝒢 and the marginal causal graph 𝒢_M_ are the same *T*_𝒢_*=T*_𝒢M_.

**Proof**: Based on Lemma 1, comparing with the true causal graph 𝒢, the marginal causal graph 𝒢_M_ may add several extra edges and induce spurious colliders but this cannot change the topological sorting. For a pair of nodes $X_{p}$ and $X_{q}$, if $X_{p}$ is ordered before $X_{q}$ in the graph 𝒢, $X_{p}$ is a parent node of $X_{q}$, that is, there is a directed path from $X_{p}$ to $X_{q}$. However, if the topological sorting changes in the graph 𝒢_M_, $X_{q}$ is ordered before $X_{p}$, that is, $X_{q}$ is a parent node of $X_{p}$ and a cycle appears. ∎

# S4 Proof of Theorem 1

**Theorem 1.** Under Assumptions 1-3, for each edge $X_{p}\to X_{q}$ in the marginal causal graph 𝒢_M_, if there is a sufficient separating set $ADJ_{X_{p}\to X_{q}}$ such that $X_{p}$ and $X_{q}$ are MR-separated, i.e. $X_{p}\perp_{MR} X_{q}|ADJ_{X_{p}\to X_{q}}$, then there is no direct edge from $X_{p}$ to $X_{q}$ in the true causal graph 𝒢.

**Proof**: Firstly, given a number of valid IVs satisfying Assumption 3, MVMR overcomes the influence of unobserved confounders on the causal estimation, and estimates the direct effect of $X_{p}$ on $X_{q}$ after adjusting for the genetic associations with $ADJ_{X_{p}\to X_{q}}$. Taking two continuous phenotypes as an example, the linear model of $X_{p}$, $X_{q}$ and $ADJ_{X_{p}\to X_{q}}$ is

$X_{q}=a_{p\to q}X_{p}+a_{ADJ}X_{ADJ_{X_{p}\to X_{q}}}+U_{pq}+\varepsilon_{X_{q}}$,

where $a_{p\to q}$ is the direct causal effect of $X_{p}$ on $X_{q}$ after adjusting for $ADJ_{X_{p}\to X_{q}}$ and U. Then we have

$\mathrm{cov}(X_{q},G_{j})=a_{p\to q}\mathrm{cov}(X_{p},G_{j})+a_{ADJ}\mathrm{cov}(X_{ADJ_{X_{p}\to X_{q}}},G_{j})+\mathrm{cov}(U_{pq}+\varepsilon_{X_{q}},G_{j})$.

If $G_{j}$ is a valid IV satisfying Assumption 4, $\mathrm{cov}(U_{pq}+\varepsilon_{X_{q}},G_{j})=0$. Divide both sides of this equation by $\mathrm{var}(G_{j})$,

$\frac{\mathrm{cov}(X_{q},G_{j})}{\mathrm{var}(G_{j})}=a_{p\to q}\frac{\mathrm{cov}(X_{p},G_{j})}{\mathrm{var}(G_{j})}+a_{ADJ}\frac{\mathrm{cov}(X_{ADJ_{X_{p}\to X_{q}}},G_{j})}{\mathrm{var}(G_{j})}$,

which can be simplified to

$\beta_{X_{qj}}=a_{p\to q}\beta_{X_{pj}}+a_{ADJ}\beta_{X_{ADJ_{X_{p}\to X_{q}}j}}$.

Thus the direct effect of $X_{p}$ on $X_{q}$ can be estimated by adjusting for the genetic associations with $ADJ_{X_{p}\to X_{q}}$ using MVMR (model (3)). For two nodes $X_{p}$ and $X_{q}$, there is a sufficient separating set $ADJ_{X_{p}\to X_{q}}$ satisfying $X_{p}\perp_{MR} X_{q}|ADJ_{X_{p}\to X_{q}}$, then $X_{p}$ and $X_{q}$ are not directly linked in graph 𝒢_M_. If directed edge $X_{p}\to X_{q}$ exists in the true causal graph 𝒢, E_𝒢_ is no more a subset of E_𝒢M_, which is contrary to Lemma 1. ∎

# S5 Topological sorting and DFS algorithm

Topological sorting for a DAG is a linear ordering of vertices such that for every directed edge $X_{p}\to X_{q}$, vertex $X_{p}$ comes before $X_{q}$ in the ordering. This ensures that parent nodes will be ordered before their child nodes, and honors the forward direction of edges in the ordering. The DFS algorithm loops through each node of the graph, in an arbitrary order. DFS terminates when it hits any node that has already been visited since the beginning of the topological sort or the node has no outgoing edges. Each node *X* gets prepended to the output list only after considering all other nodes which depend on *X* (all descendants of *X* in the graph).

# S6 Data generation of Simulation Study 1

| Figures | Data generation | Parameters’ setting |
| --- | --- | --- |
| Figure 2 A) | $\begin{matrix} G_{1i},G_{2i},G_{3i}\sim B(2,0.3) \\ U\sim N\text{（}0,1\text{）} \\ X_{1i}=\sum_{j=1}^{g_{1}} \gamma_{1j}G_{1ji}+\sum_{j=1}^{g_{2}} \gamma_{21j}G_{2ji}+U_{i}+\varepsilon_{1_{i}} \\ X_{2i}=\beta X_{1i}+U_{i}+\varepsilon_{2_{i}} \\ X_{3i}=\sum_{j=1}^{g_{3}} \gamma_{3j}G_{3ji}+\sum_{j=1}^{g_{2}} \gamma_{22j}G_{2ji}+\alpha_{1}X_{1i}+\alpha_{2}X_{2i}+U_{i}+\varepsilon_{3_{i}} \\ \varepsilon_{1_{i}},\varepsilon_{2_{i}},\varepsilon_{3_{i}}\sim N\text{（}0,1\text{）} \end{matrix}$ | *i*: *i*-th individual $\begin{matrix} \beta=0,0.1 \\ \alpha_{1}=\alpha_{2}=0.1,0.3,0.5,0.7,0.9 \\ \gamma_{1j},\gamma_{21j},\gamma_{22j},\gamma_{3j}\sim U(0.05,0.2) \\ G_{1}+G_{3}:g_{2}=0,g_{1}=g_{3}=50 \\ G_{1}+G_{2}:g_{3}=0,g_{1}=g_{2}=50 \\ G_{1}+G_{2}+G_{3}:g_{1}=g_{2}=33,g_{3}=34 \end{matrix}$ |
| Figure 2 B) | $\begin{matrix} G_{1i},G_{2i},G_{3i}\sim B(2,0.3) \\ U\sim N\text{（}0,1\text{）} \\ X_{1i}=\sum_{j=1}^{g_{1}} \gamma_{1j}G_{1ji}+\sum_{j=1}^{g_{2}} \gamma_{21j}G_{2ji}+U_{i}+\varepsilon_{1_{i}} \\ X_{4i}=\sum_{j=1}^{g_{3}} \gamma_{3j}G_{3ji}+\sum_{j=1}^{g_{2}} \gamma_{22j}G_{2ji}+\alpha_{1}X_{1i}+U_{i}+\varepsilon_{4_{i}} \\ X_{2i}=\beta X_{1i}+\alpha_{2}X_{4i}+U_{i}+\varepsilon_{2_{i}} \\ \varepsilon_{1_{i}},\varepsilon_{2_{i}},\varepsilon_{4_{i}}\sim N\text{（}0,1\text{）} \end{matrix}$ |
| Figure 2 C) | $\begin{matrix} G_{1i},G_{2i},G_{3i}\sim B(2,0.3) \\ U\sim N\text{（}0,1\text{）} \\ X_{5i}=\sum_{j=1}^{g_{3}} \gamma_{3j}G_{3ji}+\sum_{j=1}^{g_{2}} \gamma_{22j}G_{2ji}+U_{i}+\varepsilon_{5_{i}} \\ X_{1i}=\sum_{j=1}^{g_{1}} \gamma_{1j}G_{1ji}+\sum_{j=1}^{g_{2}} \gamma_{21j}G_{2ji}+\alpha_{1}X_{5i}+U_{i}+\varepsilon_{1_{i}} \\ X_{2i}=\beta X_{1i}+\alpha_{2}X_{5i}+U_{i}+\varepsilon_{2_{i}} \\ \varepsilon_{1_{i}},\varepsilon_{2_{i}},\varepsilon_{5_{i}}\sim N\text{（}0,1\text{）} \end{matrix}$ |

We conducted a simulation study to evaluate the performance of MVMR when estimating the causal effect of the interested exposure ($X_{1}$) on an outcome ($X_{2}$). We considered three roles of another exposure in MVMR: a collider ($X_{3}$), mediator ($X_{4}$), or measured confounder ($X_{5}$) in the causal pathway from $X_{1}$ to $X_{2}$ (Figure 2 A-C).

We first generate three types of IVs: $G_{1}$: SNPs only associated with the interested exposure; $G_{2}$: SNPs associated with the interested exposure and the other exposure; $G_{3}$: SNPs only associated with the other exposure. The reason we choosethree IVs is that, in MVMR, the releavnce assumption is the IVs in must be strongly associated with the exposure or covariates. Available IVs include above three types. In practical applications, researchers cannot distinguish $G_{1}$, $G_{2}$ and $G_{3}$, thus we consider two kinds of candidate IVs sets in MVMR: (1) Union: the SNPs associated with at least one of the multiple exposures ($G_{1}+G_{2}+G_{3}$), that is, researchers select IVs sets of multiple exposures separately, and then union them to obtain the final IVs set; (2) Intersection: the SNPs associated with all the exposures simultaneously ($G_{1}+G_{2}$ for collider and mediator, $G_{2}+G_{3}$ for measured confounder), that is, researchers select IVs sets of multiple exposures separately, and then take the intersection of them.

We generated *N*=10,000 independent individuals for each variable and 1,000 repeated datasets. For IVs, we generate them from Binomial distribution *B*(2,0.3). The total number of IVs are 100, for union IVs, we generate an approximate equal number $g_{1}=g_{2}=33,g_{3}=34$ of $G_{1}$, $G_{2}$ and $G_{3}$, respectively; for intersection IVs, we generate 50 IVs for two types of IVs ($G_{1}\mathrm{and}G_{2}$ for collider and mediator, $G_{2}\mathrm{and} G_{3}$ for measured confounder). The effects of IVs on the interested exposure or the other exposure ($\gamma_{1j},\gamma_{21j},\gamma_{22j},\gamma_{3j}$) are generated from uniform distribution *U*(0.05, 0.2). The unmeasured confounder U is generated from normal distribution *N*(0, 1). We consider the null causal effect $\beta=0$ and positive causal effect $\beta=0.1$, and we varying the effect between the other exposure ($X_{3}$, $X_{4}$ or $X_{5}$) and the interested exposure ($X_{1}$) or the outcome ($X_{2}$): $\alpha_{1}$ and $\alpha_{2}$, from 0.1 to 0.9. Then we generate Figure 2 A) as following (collider):

$$\begin{matrix} X_{1i}=\sum_{j=1}^{g_{1}} \gamma_{1j}G_{1ji}+\sum_{j=1}^{g_{2}} \gamma_{21j}G_{2ji}+U_{i}+\varepsilon_{1_{i}} \\ X_{2i}=\beta X_{1i}+U_{i}+\varepsilon_{2_{i}} \\ X_{3i}=\sum_{j=1}^{g_{3}} \gamma_{3j}G_{3ji}+\sum_{j=1}^{g_{2}} \gamma_{22j}G_{2ji}+\alpha_{1}X_{1i}+\alpha_{2}X_{2i}+U_{i}+\varepsilon_{3_{i}} \end{matrix}$$

We generate Figure 2 B) as following (mediator):

$$\begin{matrix} X_{1i}=\sum_{j=1}^{g_{1}} \gamma_{1j}G_{1ji}+\sum_{j=1}^{g_{2}} \gamma_{21j}G_{2ji}+U_{i}+\varepsilon_{1_{i}} \\ X_{4i}=\sum_{j=1}^{g_{3}} \gamma_{3j}G_{3ji}+\sum_{j=1}^{g_{2}} \gamma_{22j}G_{2ji}+\alpha_{1}X_{1i}+U_{i}+\varepsilon_{4_{i}} \\ X_{2i}=\beta X_{1i}+\alpha_{2}X_{4i}+U_{i}+\varepsilon_{2_{i}} \end{matrix}$$

We generate Figure 2 C) as following (confounder):

$$\begin{matrix} X_{5i}=\sum_{j=1}^{g_{3}} \gamma_{3j}G_{3ji}+\sum_{j=1}^{g_{2}} \gamma_{22j}G_{2ji}+U_{i}+\varepsilon_{5_{i}} \\ X_{1i}=\sum_{j=1}^{g_{1}} \gamma_{1j}G_{1ji}+\sum_{j=1}^{g_{2}} \gamma_{21j}G_{2ji}+\alpha_{1}X_{5i}+U_{i}+\varepsilon_{1_{i}} \\ X_{2i}=\beta X_{1i}+\alpha_{2}X_{5i}+U_{i}+\varepsilon_{2_{i}} \end{matrix}$$

where error terms are all generated from normal distribution N(0,1).

To assess the performance of MVMR, we plotted a boxplot to evaluate the estimation of the causal effect of $X_{1}$ on $X_{2}$ and calculated the type I error rate for the null causal effect and statistical power to detect the non-zero causal effect. The nominal level was set to 0.05.

# S7 Details of the 8 competing methods were applied in the simulation

We compared our method with eight published methods: BIMMER, cGAUGE based on IVW, MR Egger and MR PRESSO, HC algorithm incorporating genetic anchors (based on genetic risk score or the most significant SNP) and MRPC algorithm (based on genetic risk score or the most significant SNP).

MRSL and BIMMER were implemented using GWAS summary data. After generating the individual data (data generation process was described in the section 2.2 in the main text), we obtained the GWAS summary data by using linear regression (logistic regression) of continuous (binary) variables on each SNP. MRSL and BIMMER were implemented by R package *MRSL* (https://github.com/hhoulei/MRSL) and *bimmer* (https://github.com/brielin/bimmer).

cGAUGE based on IVW, MR Egger and MR PRESSO were implemented using individual genetic and phenotypic data. cGAUGE was implemented by R package cGAUGE (https://github.com/david-dd-amar/cGAUGE/).

HC algorithm incorporating genetic anchors and MRPC algorithm were also implemented using individual genetic and phenotypic data. When using all the SNPs, HC and MRPC algorithm have huge computational complexity and cannot output the result. Thus we consider two scenarios, the first is select the SNP which has the most significant relationship (that is, p value is the smallest) with the variable of interested; the second is for each variable, we construct a genetic risk score by combining the information of all the SNPs. So each variable has only one instrumental variable (genetic risk score or the most significant SNP), and HC and MRPC algorithm were implemented using individual phenotypic data and individual data of genetic risk score or the most significant SNP. For HC algorithm, we added the blacklist that the variables cannot affect SNPs to improve the accuracy. HC and MRPC algorithm were implemented using R packages *bnlearn* (https://cran.r-project.org/web/packages/bnlearn/index.html) and *MRPC* (https://cran.r-project.org/web/packages/MRPC/index.html).

# S8 Data generation of Simulation Study 3

We Genetic IVs are generated from binomial distribution $B(2,0.3)$. Let $X_{i}$ denote the phenotype, $G_{i}$ denote the IVs for $X_{i}$, U denote the unmeasured confounding and the $\varepsilon_{i}$ denote the error term.

For Protein-Signaling (Figure 3(A)), 8 binary phenotypes are generated from the following model:

$$\begin{matrix} P(X_{1}=1)=expit(G_{1}\alpha_{1}+U),X_{1}\sim B(1,P(X_{1}=1)) \\ P(X_{2}=1)=expit(X_{1}\beta_{2}+G_{2}\alpha_{2}+U),X_{2}\sim B(1,P(X_{2}=1)) \\ P(X_{3}=1)=expit(X_{1}\beta_{13}+X_{2}\beta_{23}+G_{3}\alpha_{3}+U),X_{3}\sim B(1,P(X_{3}=1)) \\ P(X_{4}=1)=expit(X_{1}\beta_{14}+X_{2}\beta_{24}+G_{4}\alpha_{4}+U),X_{4}\sim B(1,P(X_{4}=1)) \\ P(X_{5}=1)=expit(X_{2}\beta_{25}+X_{4}\beta_{45}+G_{5}\alpha_{5}+U),X_{5}\sim B(1,P(X_{5}=1)) \\ P(X_{6}=1)=expit(X_{2}\beta_{26}+X_{5}\beta_{56}+G_{6}\alpha_{6}+U),X_{6}\sim B(1,P(X_{6}=1)) \\ P(X_{7}=1)=expit(X_{1}\beta_{17}+X_{2}\beta_{27}+G_{7}\alpha_{7}+U),X_{7}\sim B(1,P(X_{7}=1)) \\ P(X_{8}=1)=expit(X_{1}\beta_{18}+X_{2}\beta_{28}+G_{8}\alpha_{8}+U),X_{8}\sim B(1,P(X_{8}=1)) \\ U\sim N(0,1) \end{matrix}$$

For Gene regulatory (Figure 3(B)), 7 continuous phenotypes are generated from the following model:

$$\begin{matrix} X_{1}=G_{1}\alpha_{1}+U+\varepsilon_{1} \\ X_{2}=G_{2}\alpha_{2}+U+\varepsilon_{1} \\ X_{3}=X_{2}\beta_{23}+G_{3}\alpha_{3}+U+\varepsilon_{3} \\ X_{4}=X_{3}\beta_{34}+G_{4}\alpha_{4}+U+\varepsilon_{4} \\ X_{5}=X_{4}\beta_{45}+G_{5}\alpha_{5}+U+\varepsilon_{5} \\ X_{6}=X_{4}\beta_{46}+G_{6}\alpha_{6}+U+\varepsilon_{6} \\ X_{7}=X_{4}\beta_{17}+X_{4}\beta_{7}+X_{6}\beta_{67}+G_{7}\alpha_{7}+U+\varepsilon_{7} \\ \varepsilon_{1},\varepsilon_{2},\varepsilon_{3},\varepsilon_{4},\varepsilon_{5},\varepsilon_{6},\varepsilon_{7}\sim N(0,1),\mathrm{U}\sim N(0,1) \end{matrix}$$

For Metabolic syndrome (Figure 3(C)), 3 binary and 5 continuous phenotypes are generated from the following model:

$$\begin{matrix} X_{1}=G_{1}\alpha_{1}+U+\varepsilon_{1} \\ X_{2}=X_{1}\beta_{12}+G_{2}\alpha_{2}+U+\varepsilon_{2} \\ X_{3}=X_{1}\beta_{13}+G_{3}\alpha_{3}+U+\varepsilon_{3} \\ X_{4}=X_{3}\beta_{34}+G_{4}\alpha_{4}+U+\varepsilon_{4} \\ X_{5}=X_{3}\beta_{35}+G_{5}\alpha_{5}+U+\varepsilon_{5} \\ P(X_{6}=1)=expit(X_{2}\beta_{26}+X_{4}\beta_{46}+X_{5}\beta_{56}+G_{6}\alpha_{6}+U),X_{6}\sim B(1,P(X_{6}=1)) \\ P(X_{7}=1)=expit(X_{6}\beta_{67}+G_{7}\alpha_{7}+U),X_{7}\sim B(1,P(X_{7}=1)) \\ P(X_{8}=1)=expit(X_{4}\beta_{48}+X_{5}\beta_{58}+X_{7}\beta_{78}+G_{8}\alpha_{8}+U),X_{7}\sim B(1,P(X_{8}=1)) \\ \varepsilon_{1},\varepsilon_{2},\varepsilon_{3},\varepsilon_{4},\varepsilon_{5}\sim N(0,1),U\sim N(0,1) \end{matrix}$$

We generate 10,000 independent individuals for each variable and 1,000 repeated datasets. Then we generate summary data based on above individual data. Summary statistics can be obtained by linear (or logistic) regressions of each phenotype on genetic IVs. We generate the random graphs with 5, 10 and 15 nodes, respectively. Considering the different complexity of network, we set the probability of each edge to be present in a graph as 0.2, 0.5 and 0.8. In practice, there may be effects of different magnitude between traits, thus we consider $\beta$ follows uniform distribution with four parameter settings: U(0,0.25), U(0.25,0.5), U(0.5,0.75) and U(0.75,1) for continuous nodes, odd ratio (OR) U(1,1.5), U(1.5,2), U(2,2.5) and U(2.5,3) for binary nodes. The IVs are assumed uncorrelated, and subdivided into two categories: (1) $g_{1}$ SNPs that only predict one phenotype; (2) $g_{2}$ SNPs that predict all the phenotypes simultaneously. The marginal causal graph of MRSL is obtained by bi-directional IVW method. We vary across the number of SNPs $g_{1}$ and $g_{2}$ with 5, 10, 20, 30, 40 and 50, respectively.

# S9 Calculation formula of F1score, recall and precision

|  | True | | | |
| --- | --- | --- | --- | --- |
| Predict |  | True | False | All |
|  | Positive | TP | FP | P |
|  | Negative | FN | TN | N |
|  | All | T | F | P+N=T+F |

$$\begin{matrix} precision=\frac{TP}{TP+FP} \\ recall=\frac{TP}{TP+FN} \\ F1score=\frac{2\times recall\times precision}{recall+precision} \end{matrix}$$

# S10 Calculation formula of Relative spearman's footrule and Kendall’s tau

Let $[n]=\{1,2,...,n\}$ be a universe of elements, and $\sigma(i)$ denote the rank of the element *i*. We propose a relative Spearman's footrule to measure the distance between ranked lists:

$F_{R}(\sigma)=\frac{F(\sigma)}{n\times(n-1)}$,

where $F(\sigma)=\sum_{i} |i-\sigma(i)|$ is traditional Spearman's footrule, which is given as the sum of the absolute differences between the ranks of the two lists.

Another metric measuring the distance between ranked lists is Kendall’s tau

$$K(\sigma)=\sum_{(i,j):i>j} [\sigma(i)<\sigma(j)]$$

This measures the total number of pairwise inversions.


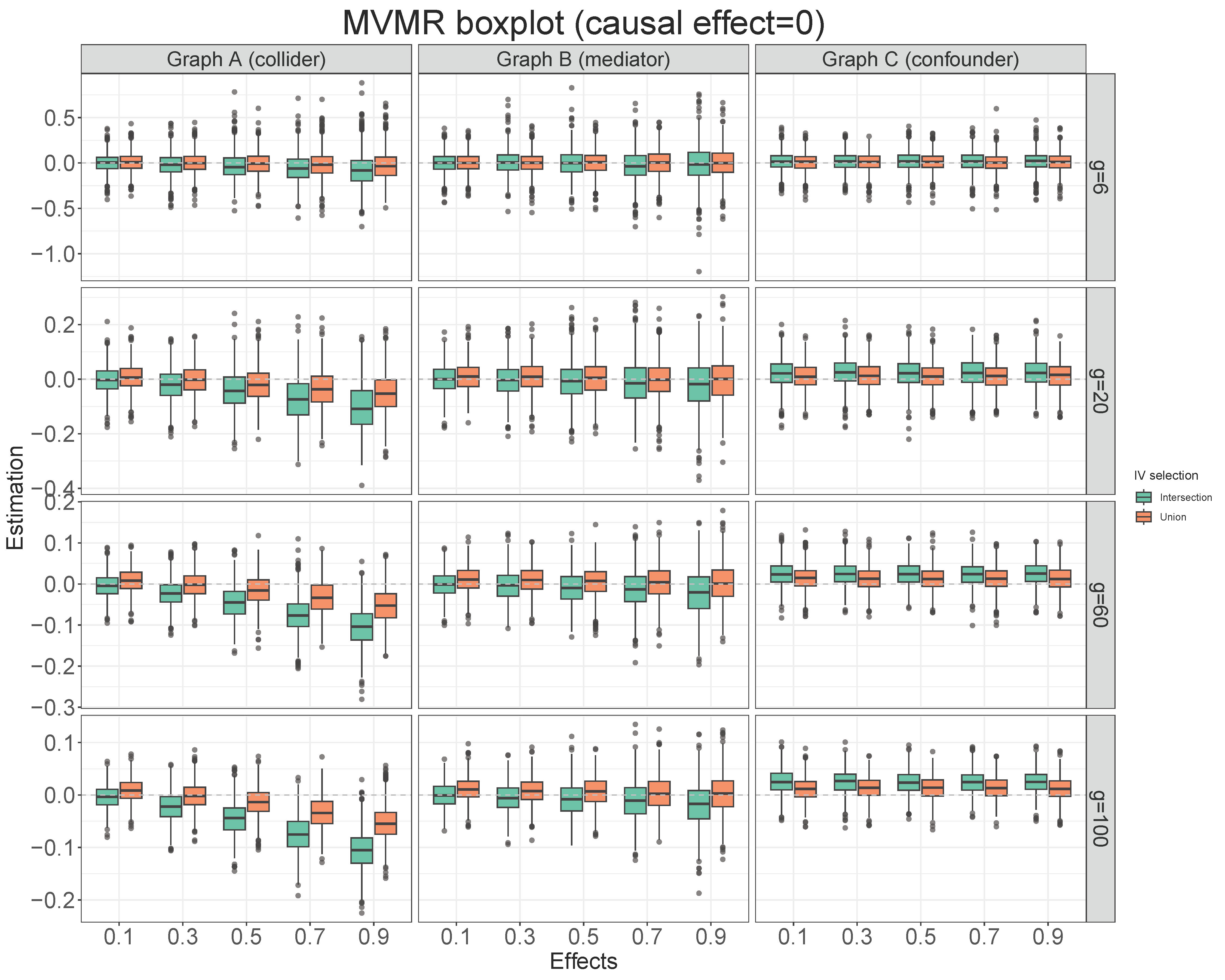


# Figure S1. Estimation of causal effect using MVMR in simulation study 1 (causal effect=0)


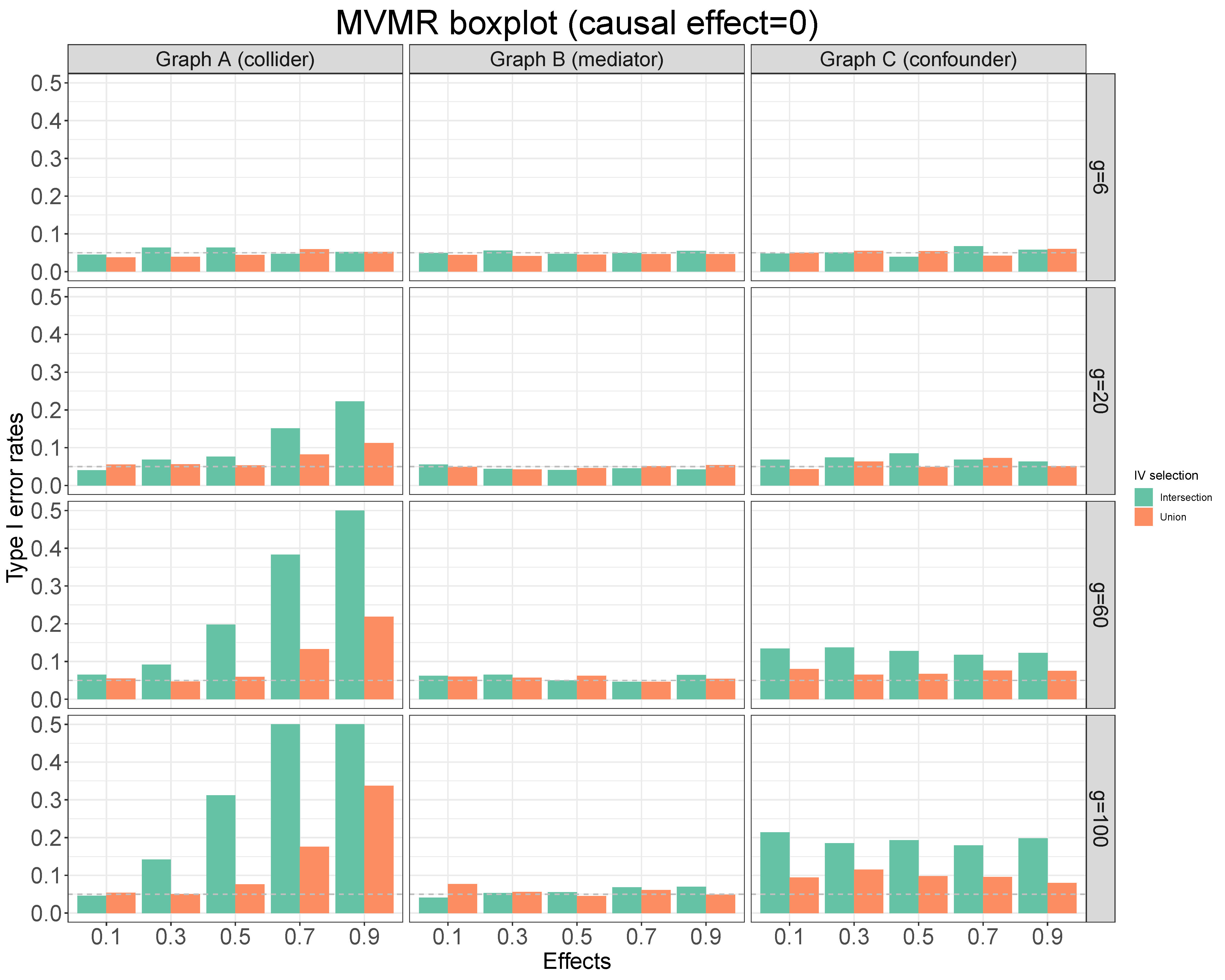


# Figure S2. Type I error rate of causal effect using MVMR in simulation study 1 (causal effect=0)


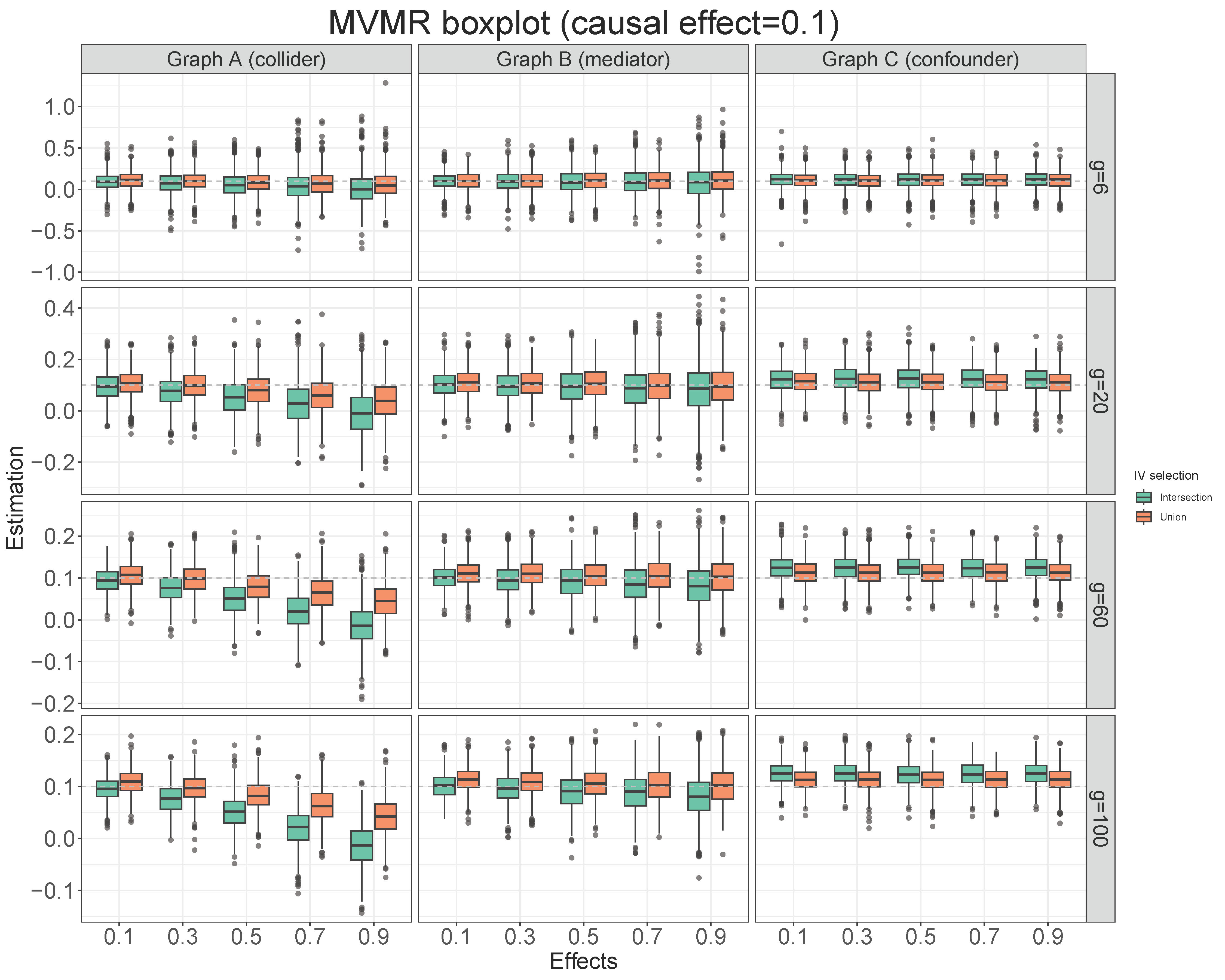


# Figure S3. Estimation of causal effect using MVMR in simulation study 1 (causal effect=0.1)


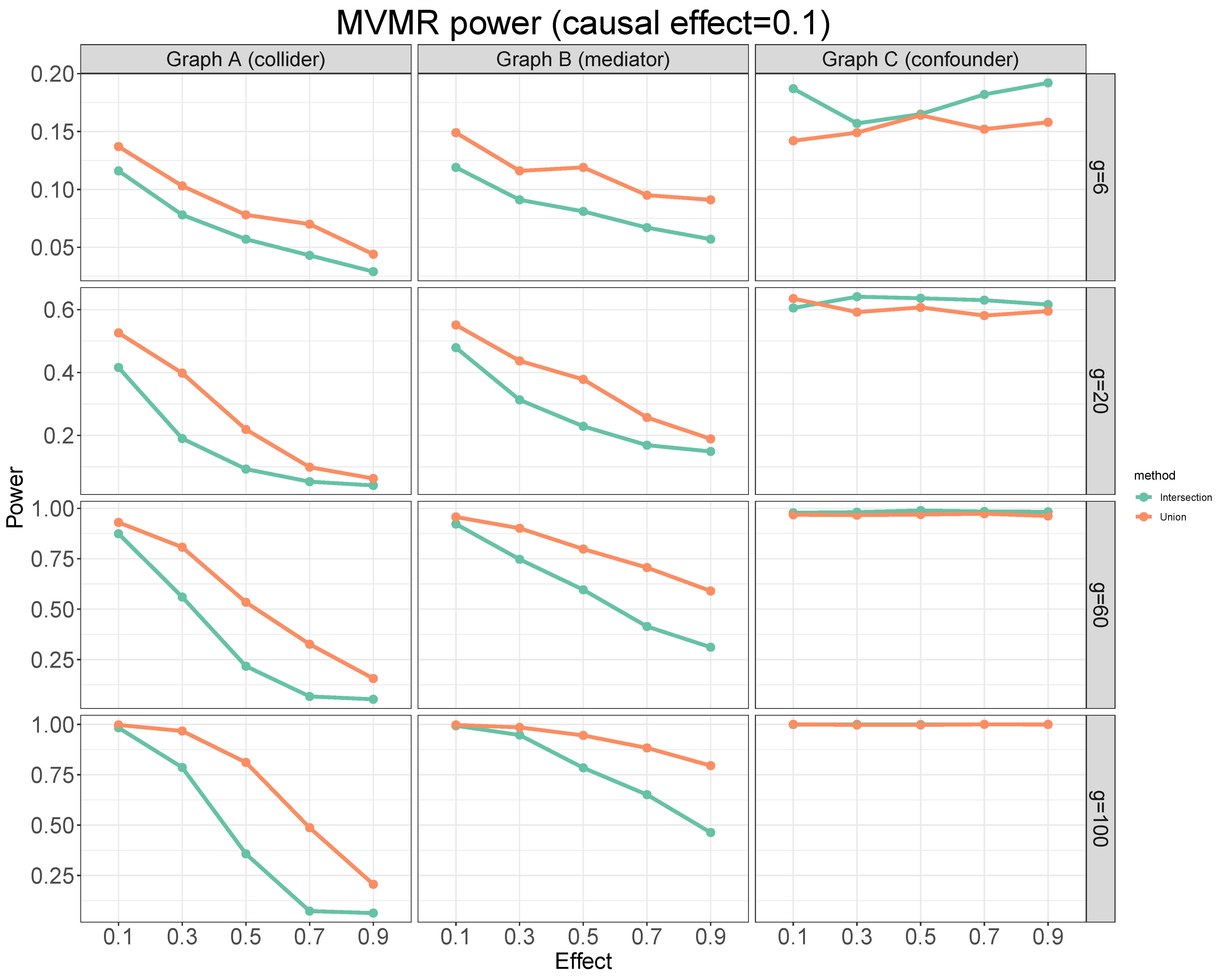


# Figure S4. Statistical power of causal effect using MVMR in simulation study 1 (causal effect=0.1)


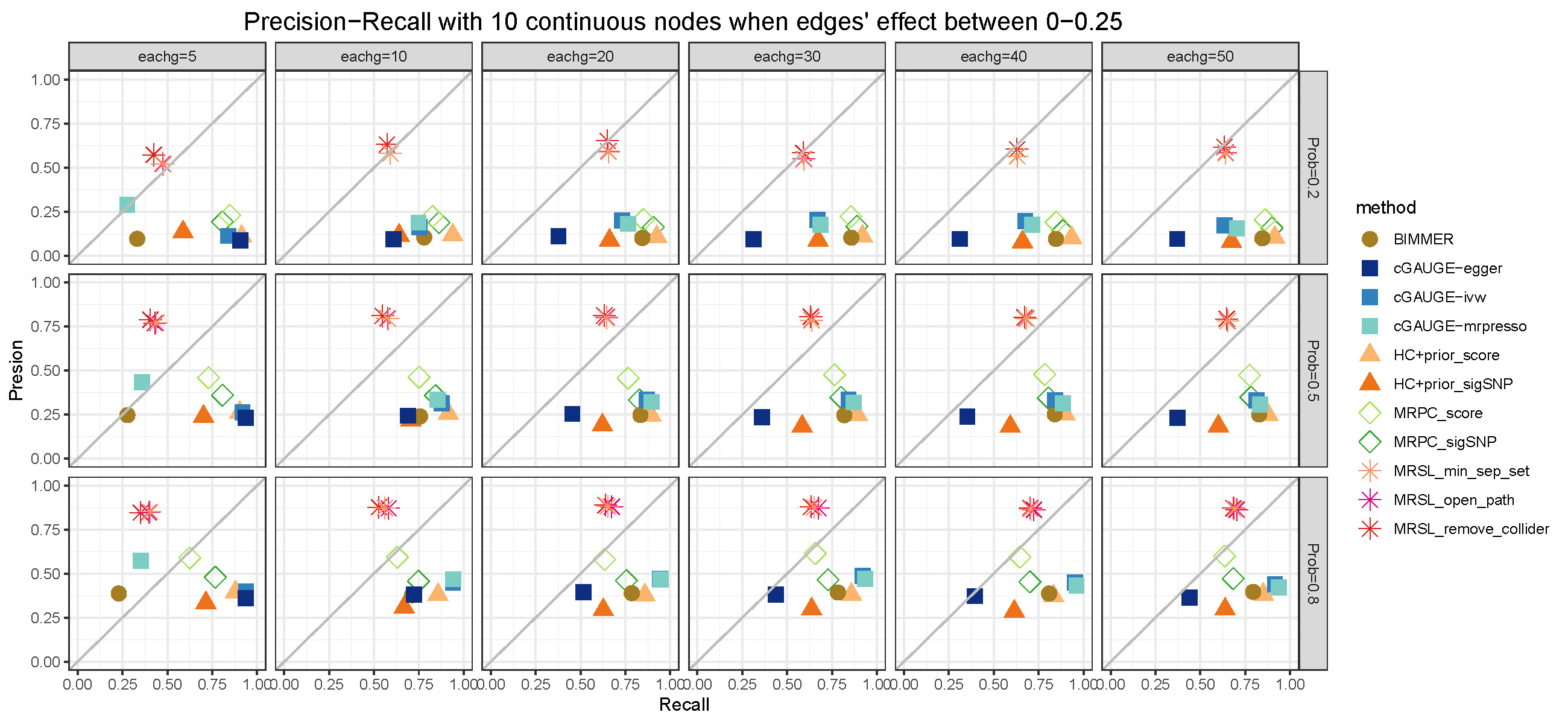


# Figure S5. Precision−Recall with 10 continuous nodes when edges' effect between 0−0.25 in simulation study 2


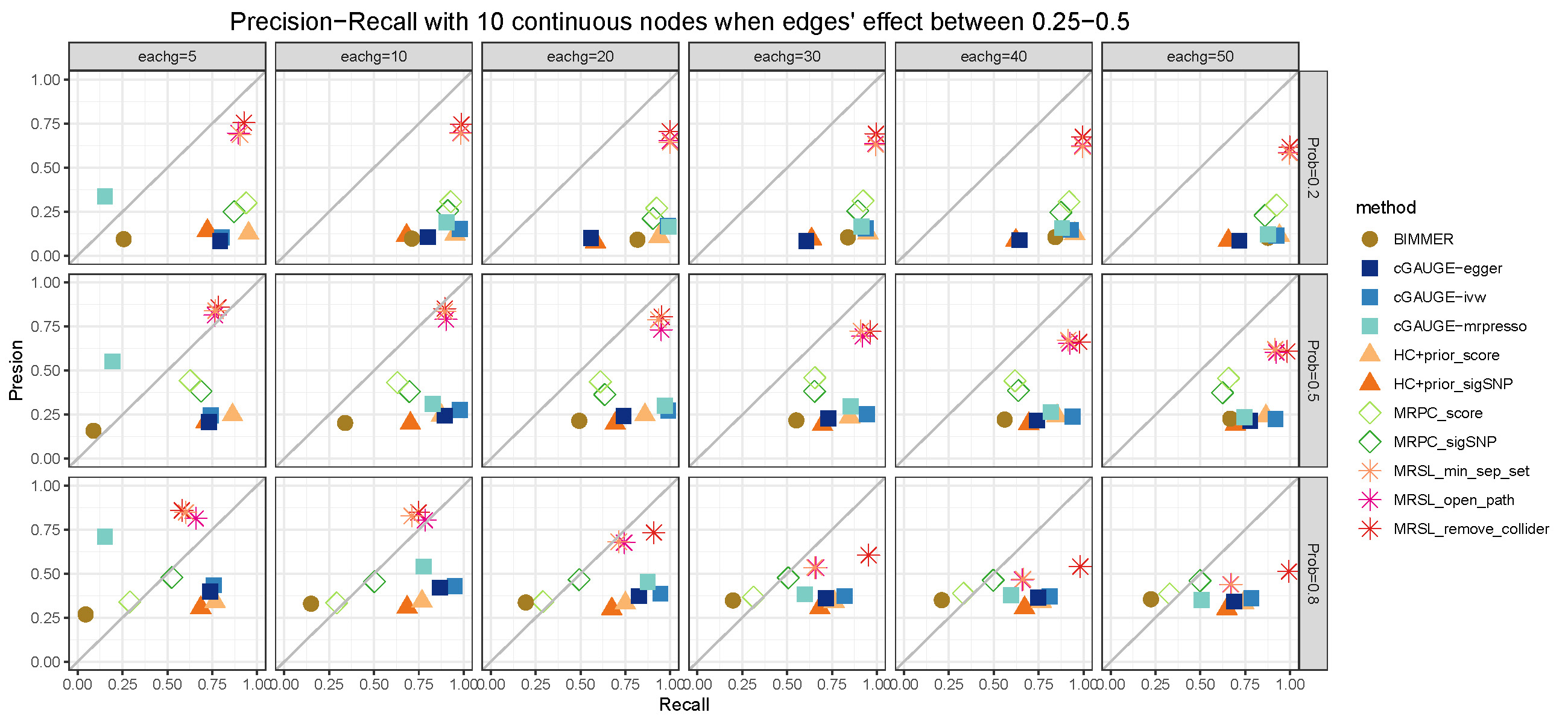


# Figure S6. Precision−Recall with 10 continuous nodes when edges' effect between 0.25−0.5 in simulation study 2


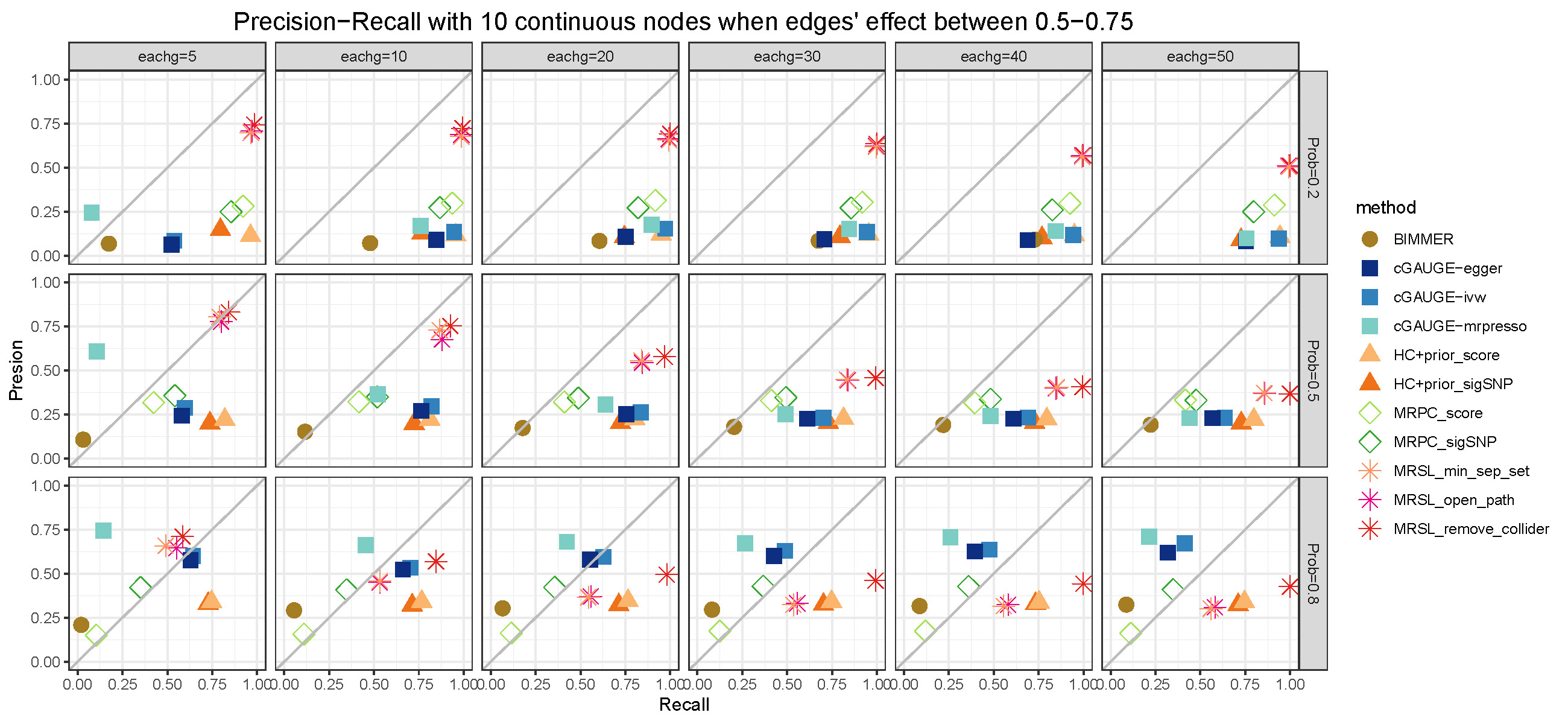


# Figure S7. Precision−Recall with 10 continuous nodes when edges' effect between 0.5−0.75 in simulation study 2


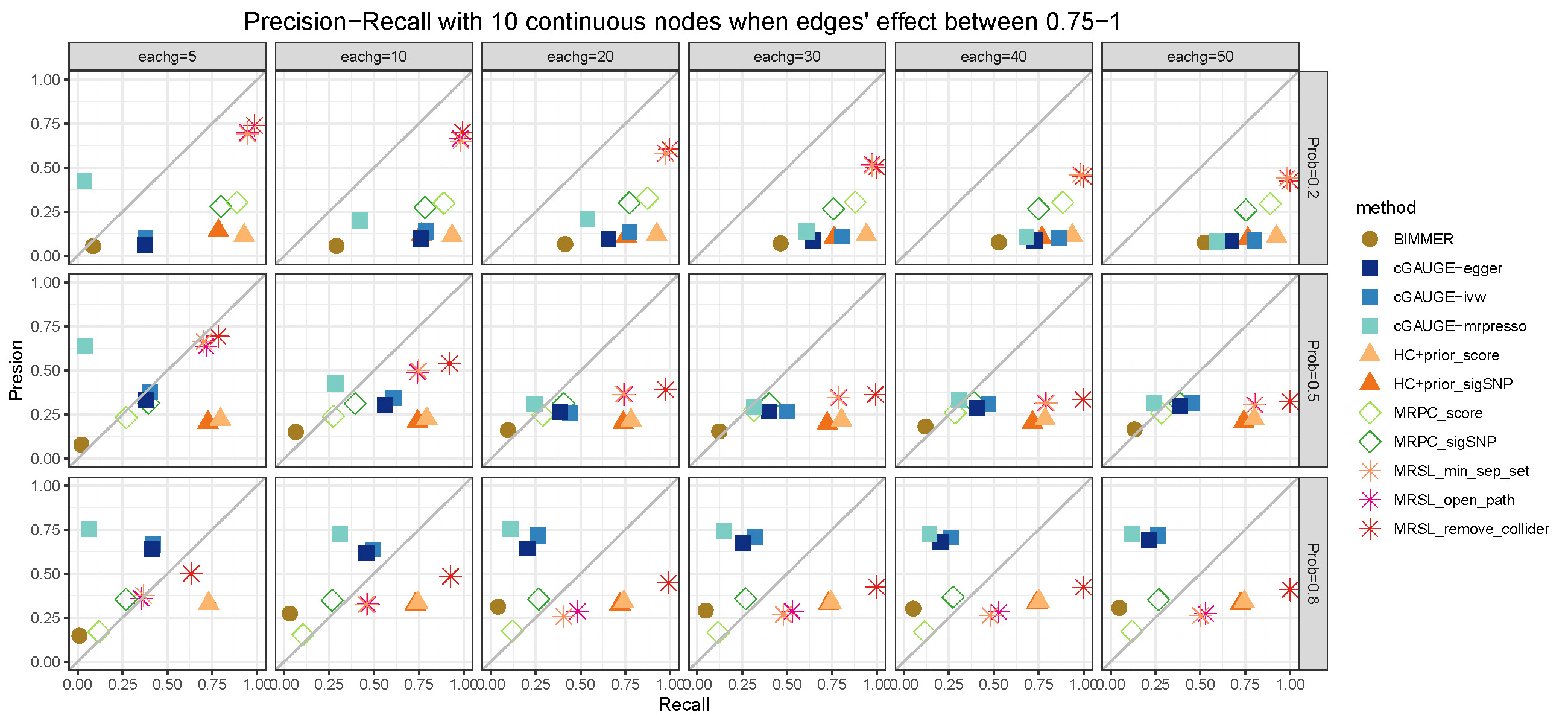


# Figure S8. Precision−Recall with 10 continuous nodes when edges' effect between 0.75−1 in simulation study 2


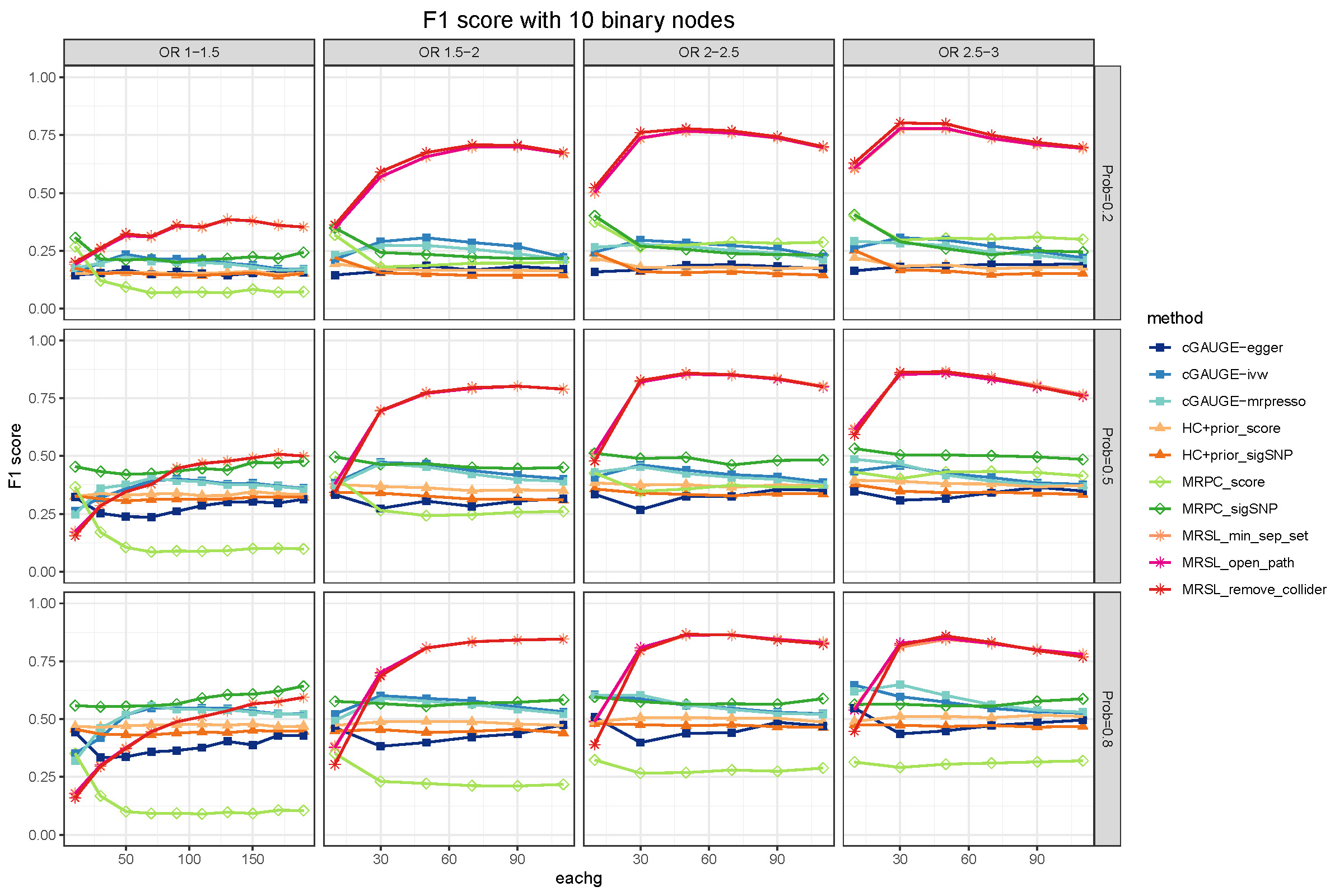


# Figure S9. F1 score with 10 binary nodes


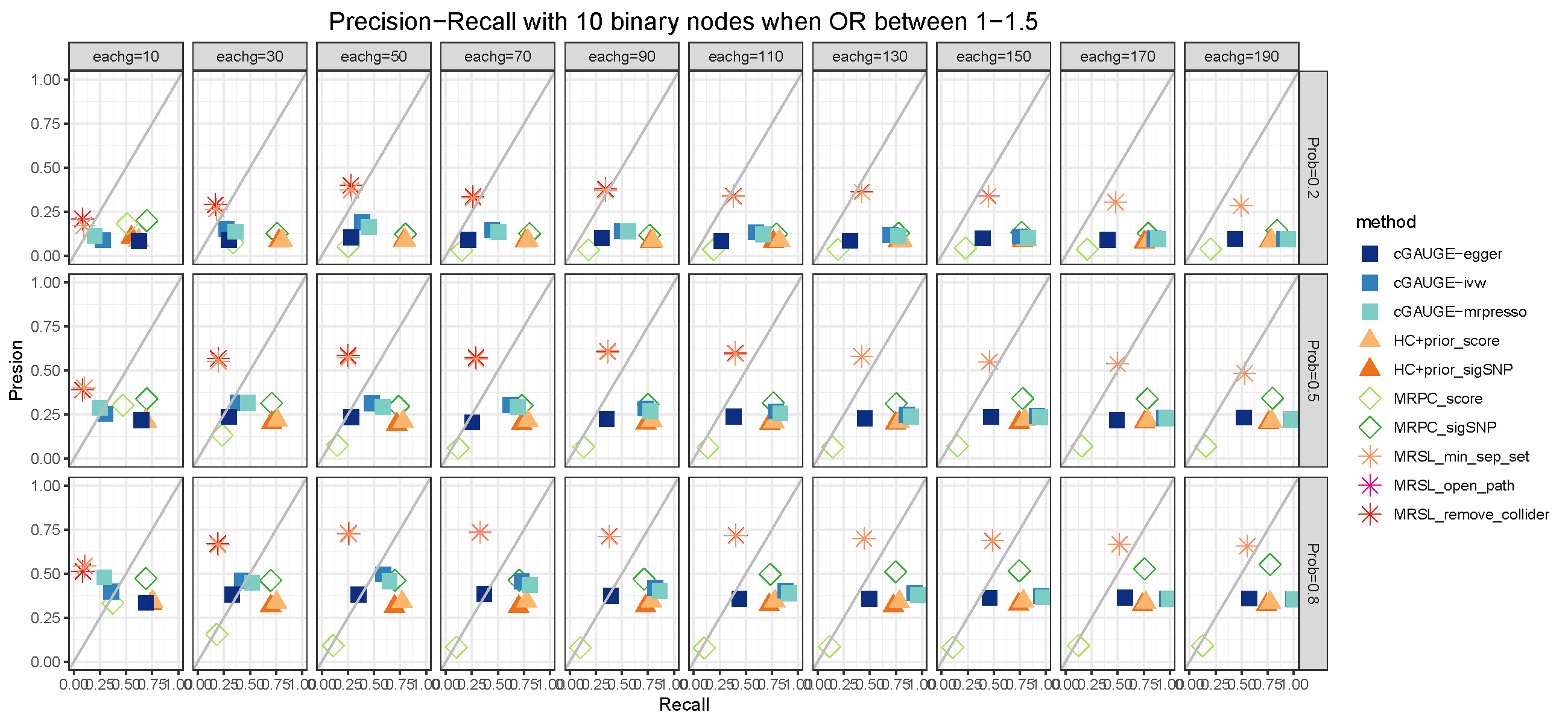


# Figure S10. Precision−Recall with 10 binary nodes when OR between 1−1.5 in simulation study 2


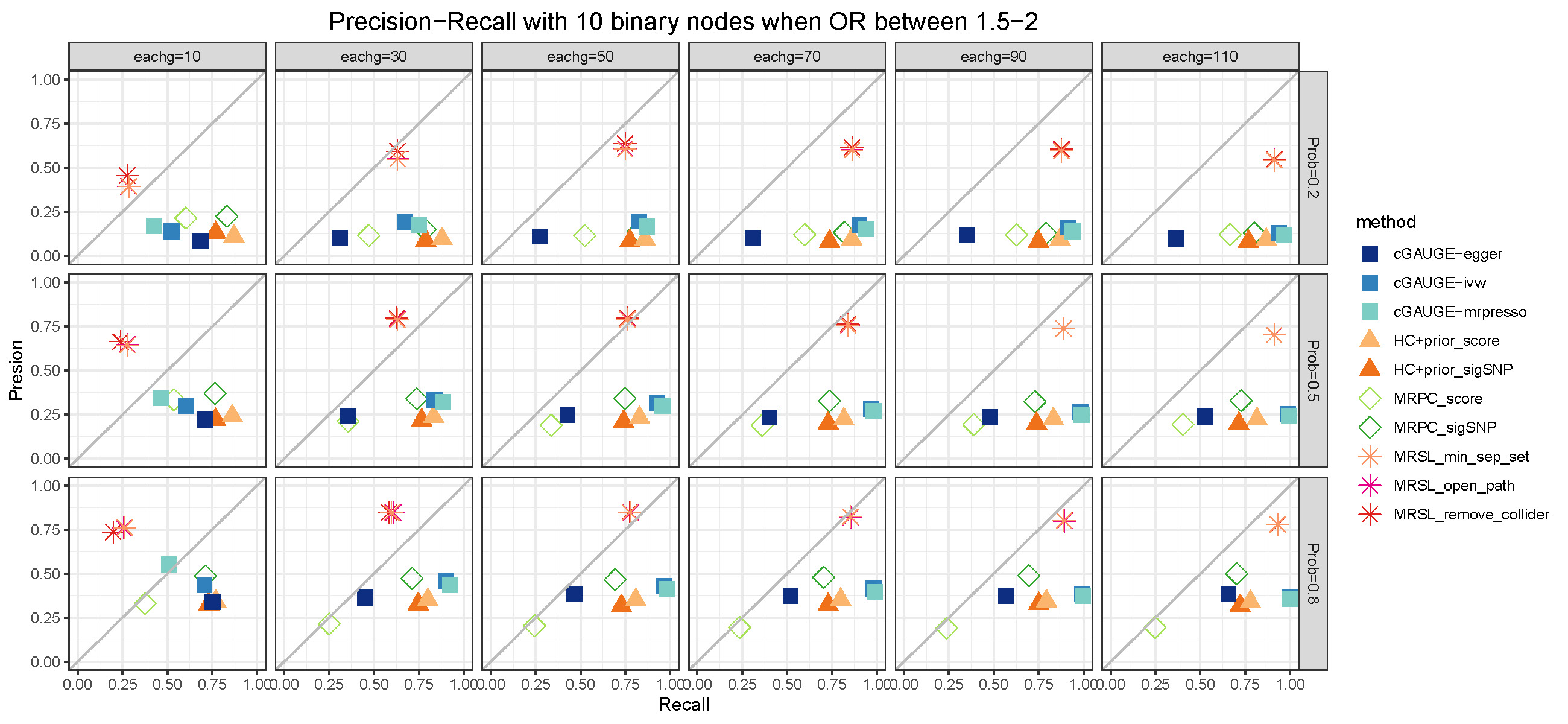


# Figure S11. Precision−Recall with 10 binary nodes when OR between 1.5−2 in simulation study 2


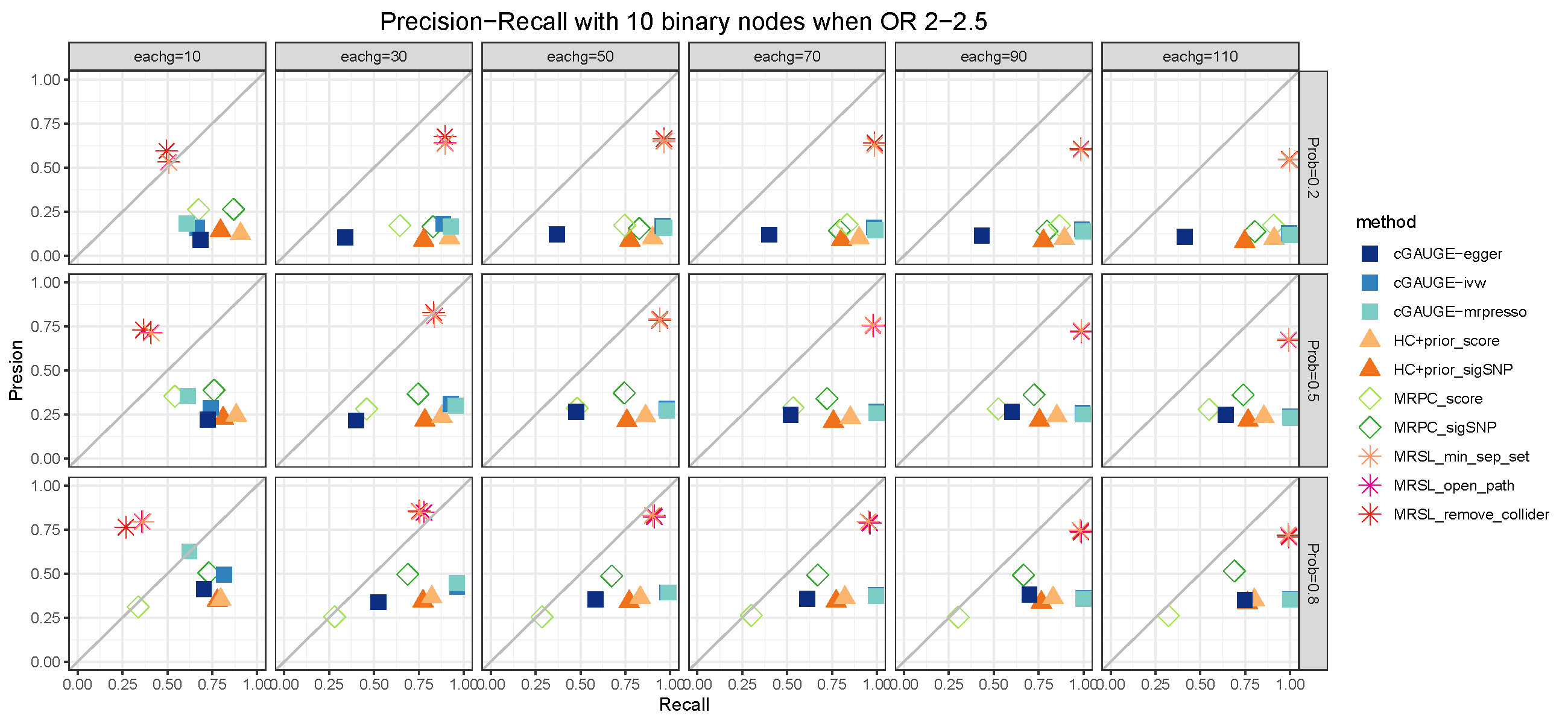


# Figure S12. Precision−Recall with 10 binary nodes when OR between 2−2.5 in simulation study 2


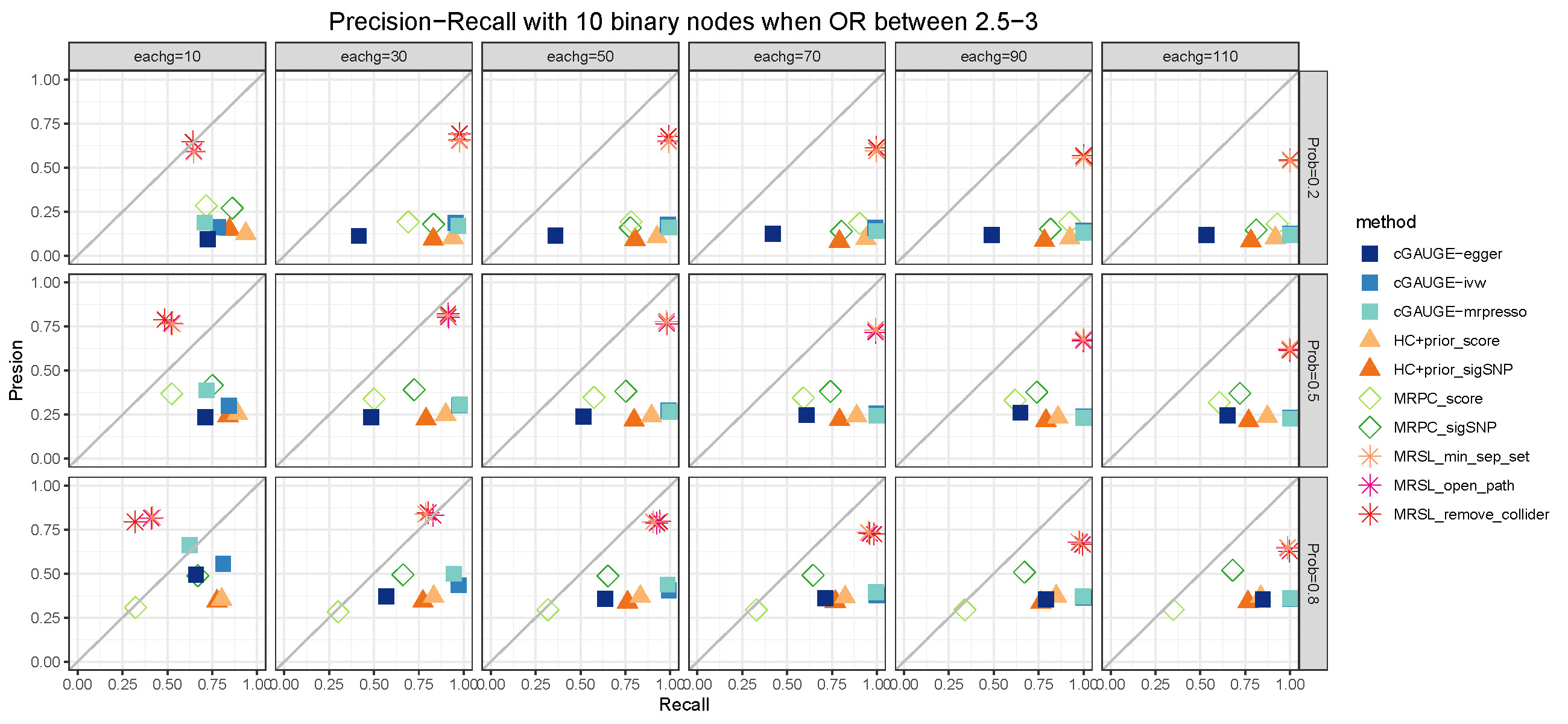


# Figure S13. Precision−Recall with 10 binary nodes when OR between 2.5−3 in simulation study 2


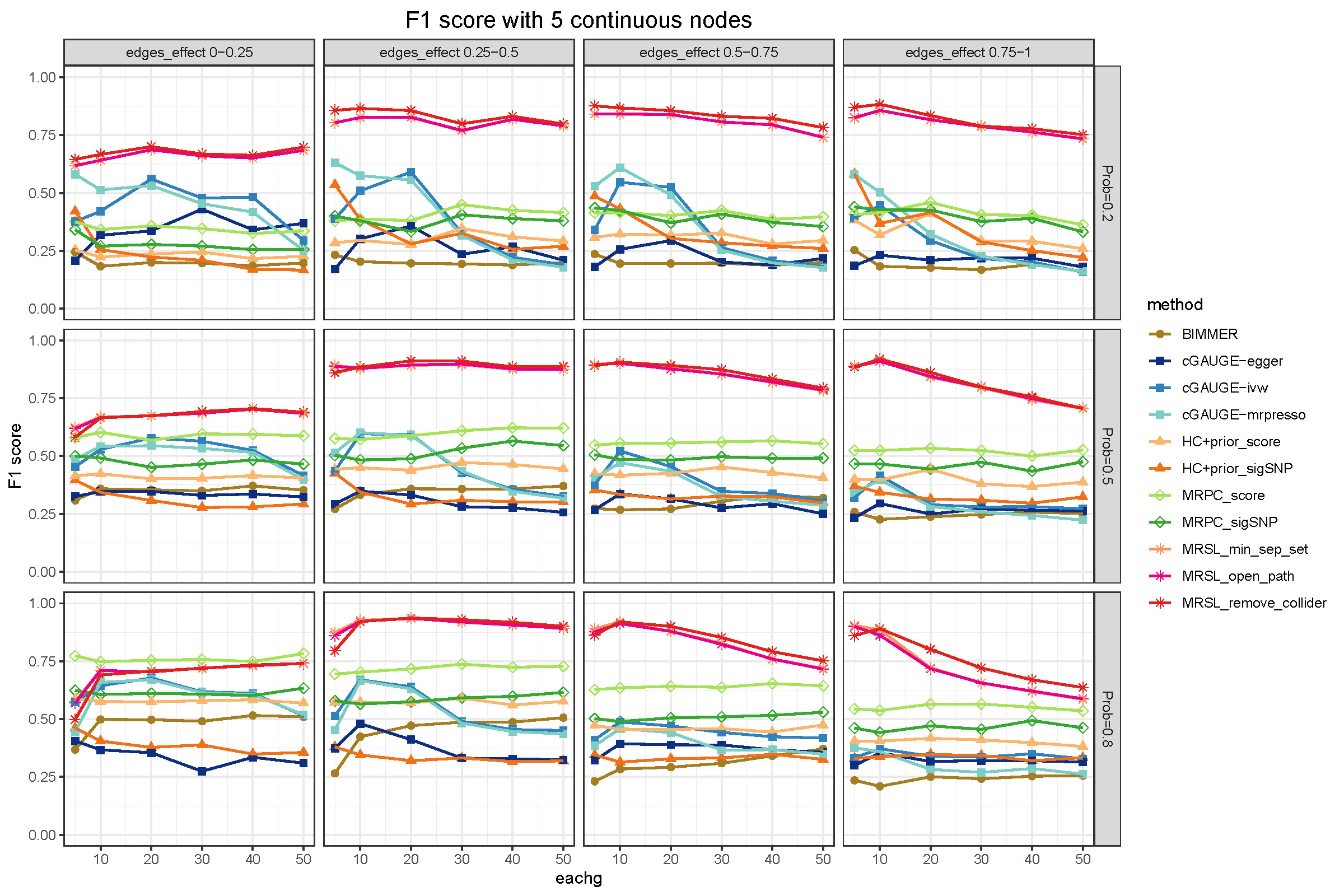


# Figure S14. F1 score with 5 continuous nodes in simulation study 2


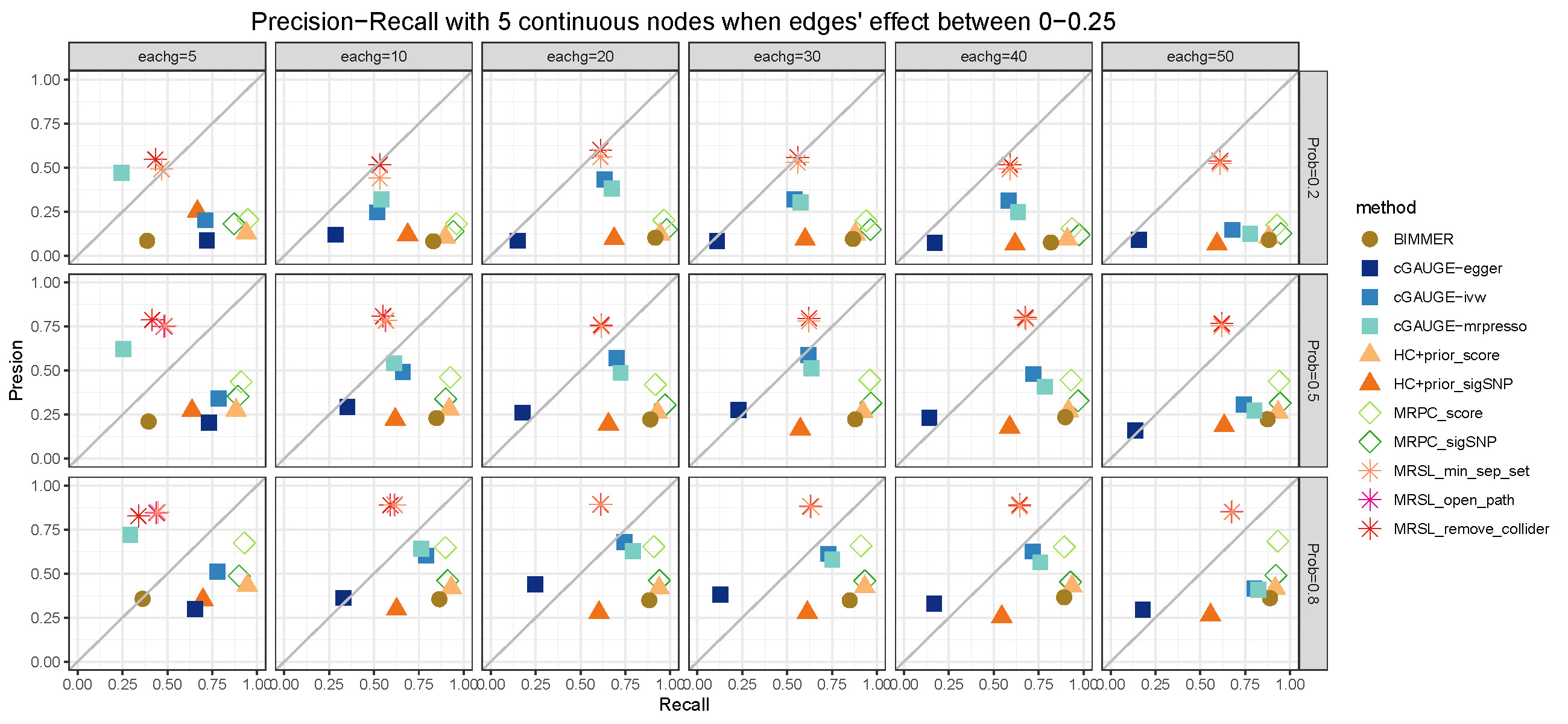


# Figure S15. Precision−Recall with 5 continuous nodes when edges' effect between 0−0.25 in simulation study 2


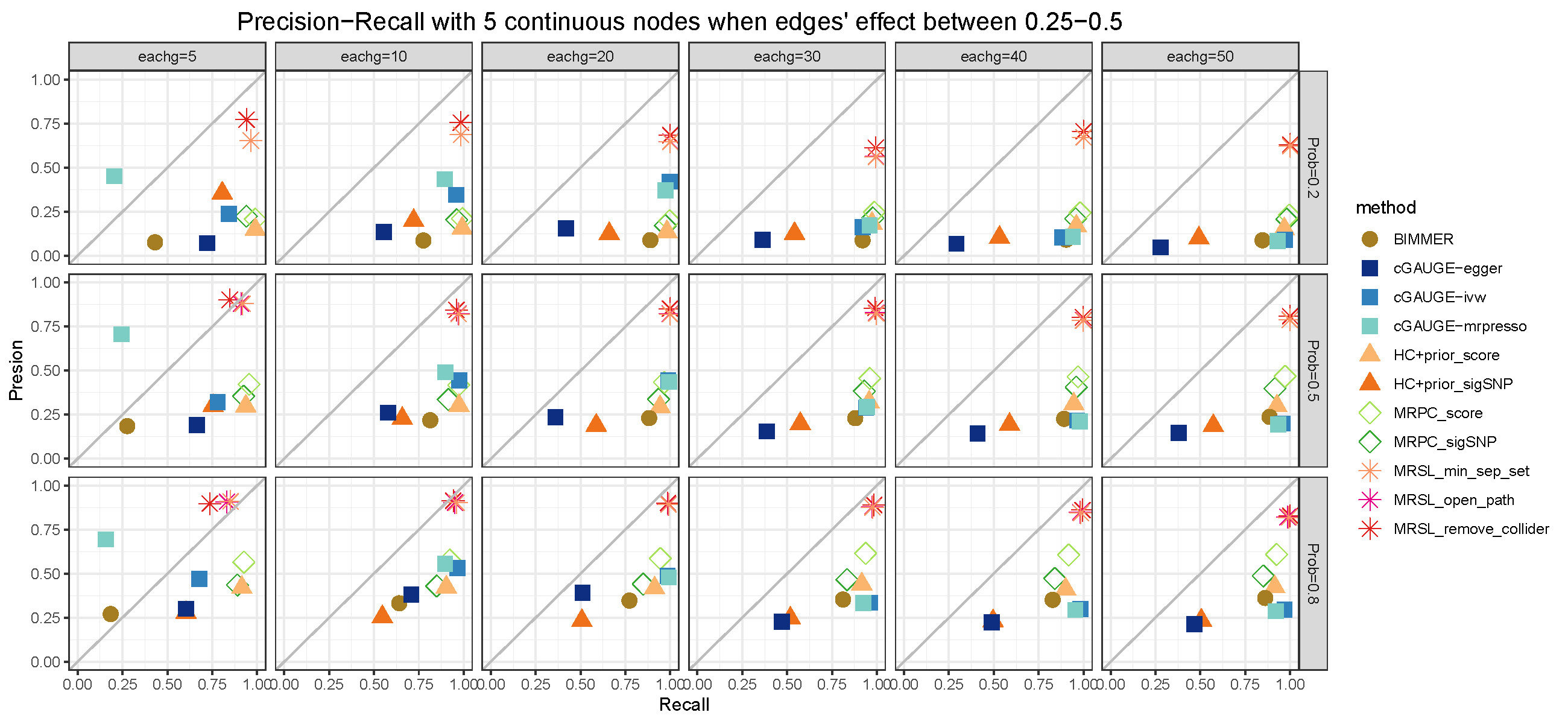


# Figure S16. Precision−Recall with 5 continuous nodes when edges' effect between 0.25−0.5 in simulation study 2


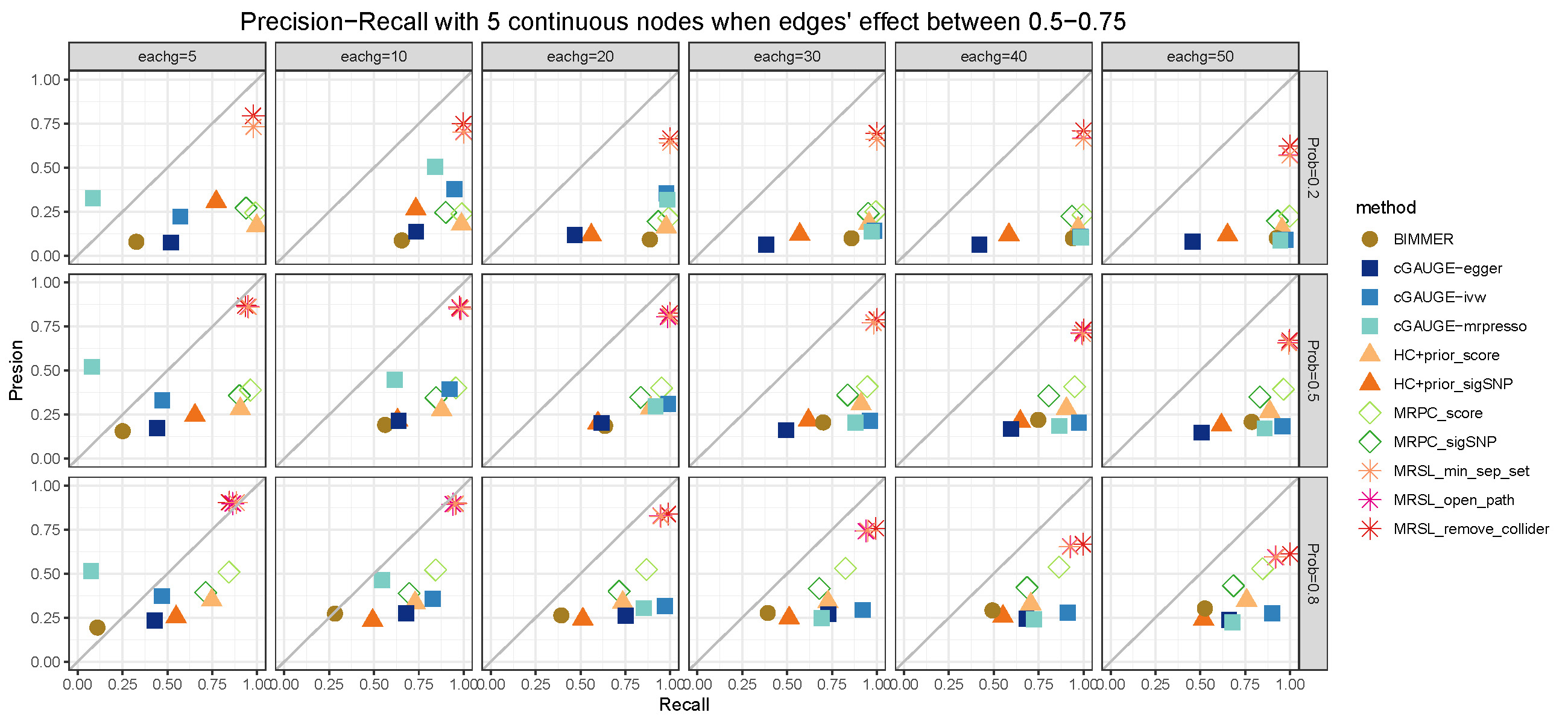


# Figure S17. Precision−Recall with 5 continuous nodes when edges' effect between 0.5−0.75 in simulation study 2


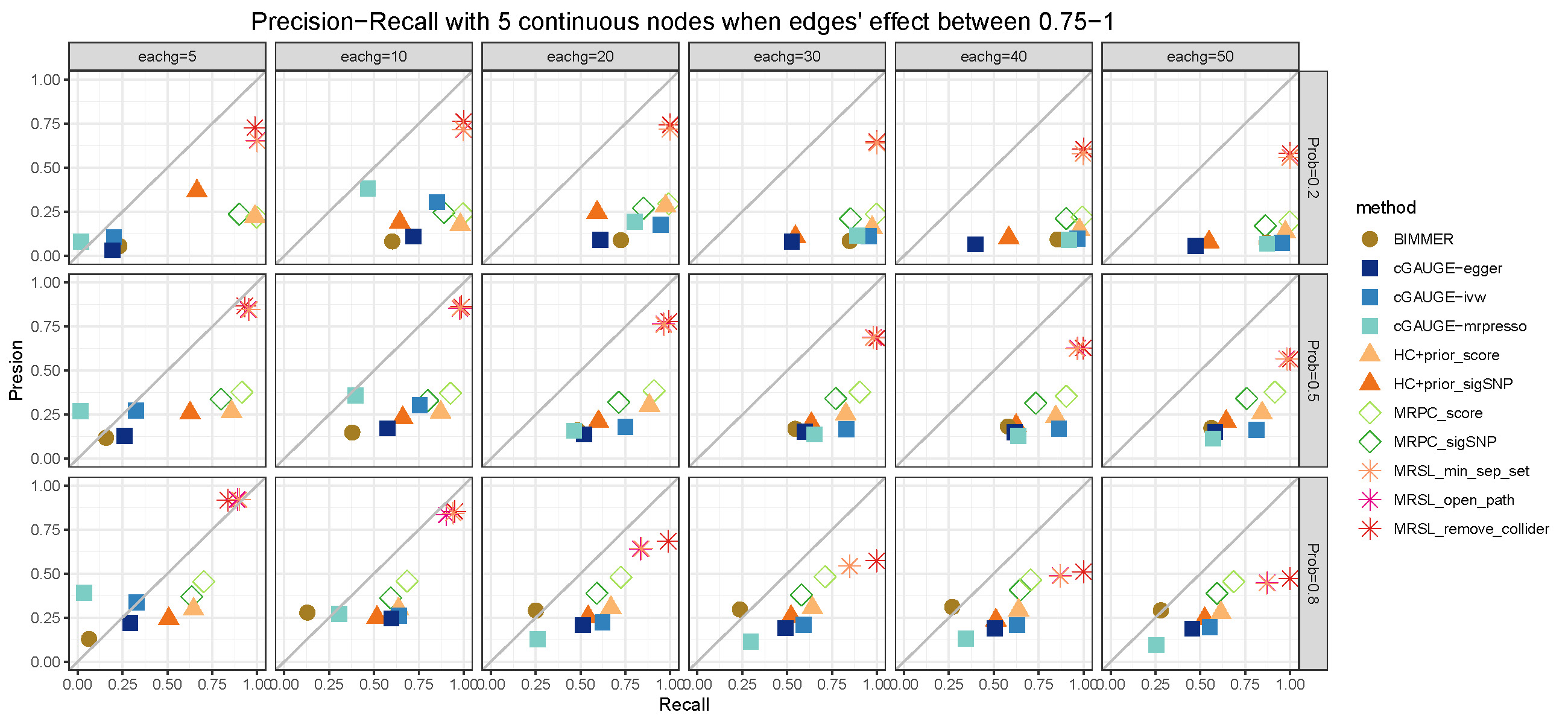


# Figure S18. Precision−Recall with 5 continuous nodes when edges' effect between 0.75−1 in simulation study 2


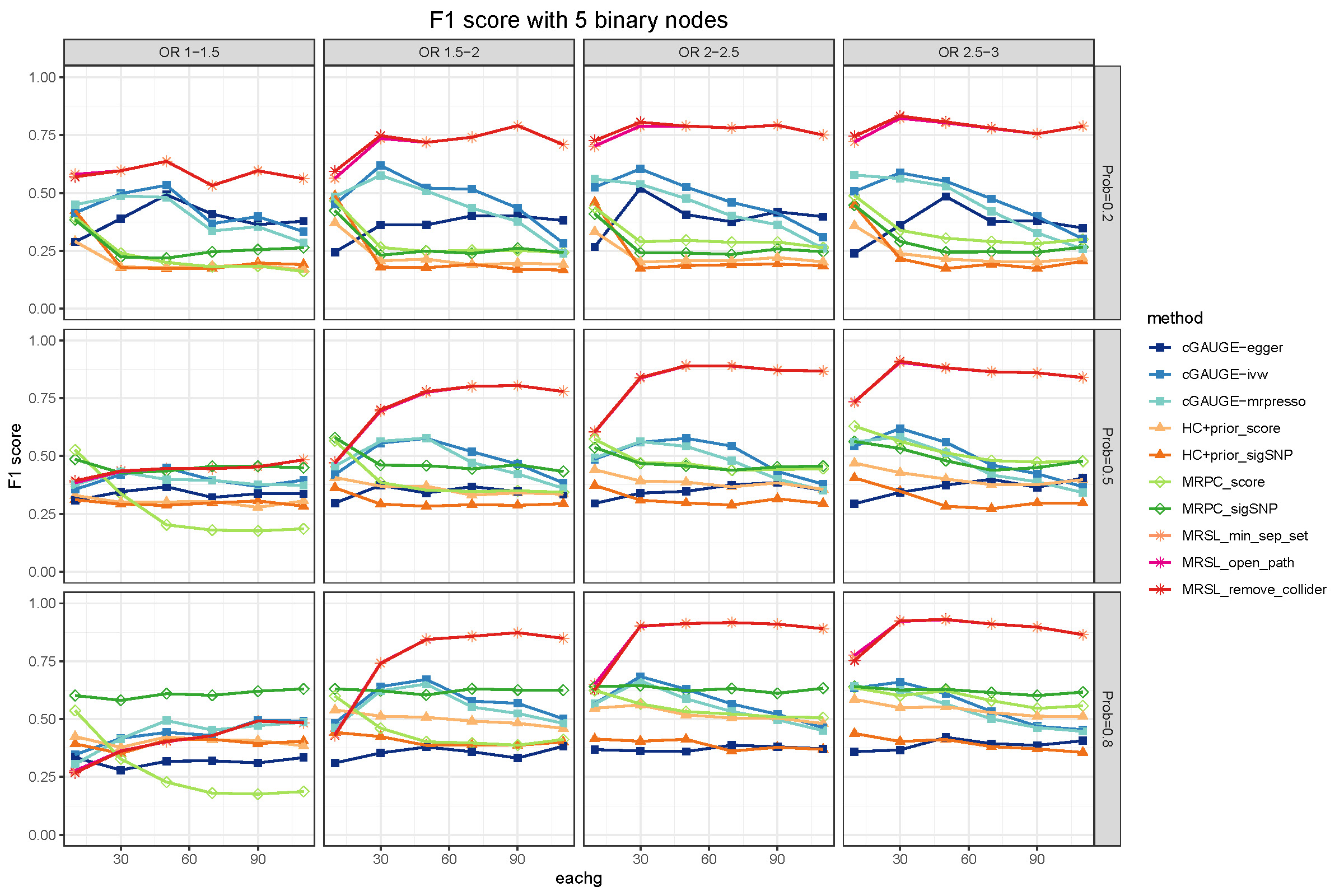


# Figure S19. F1 score with 5 binary nodes in simulation study 2


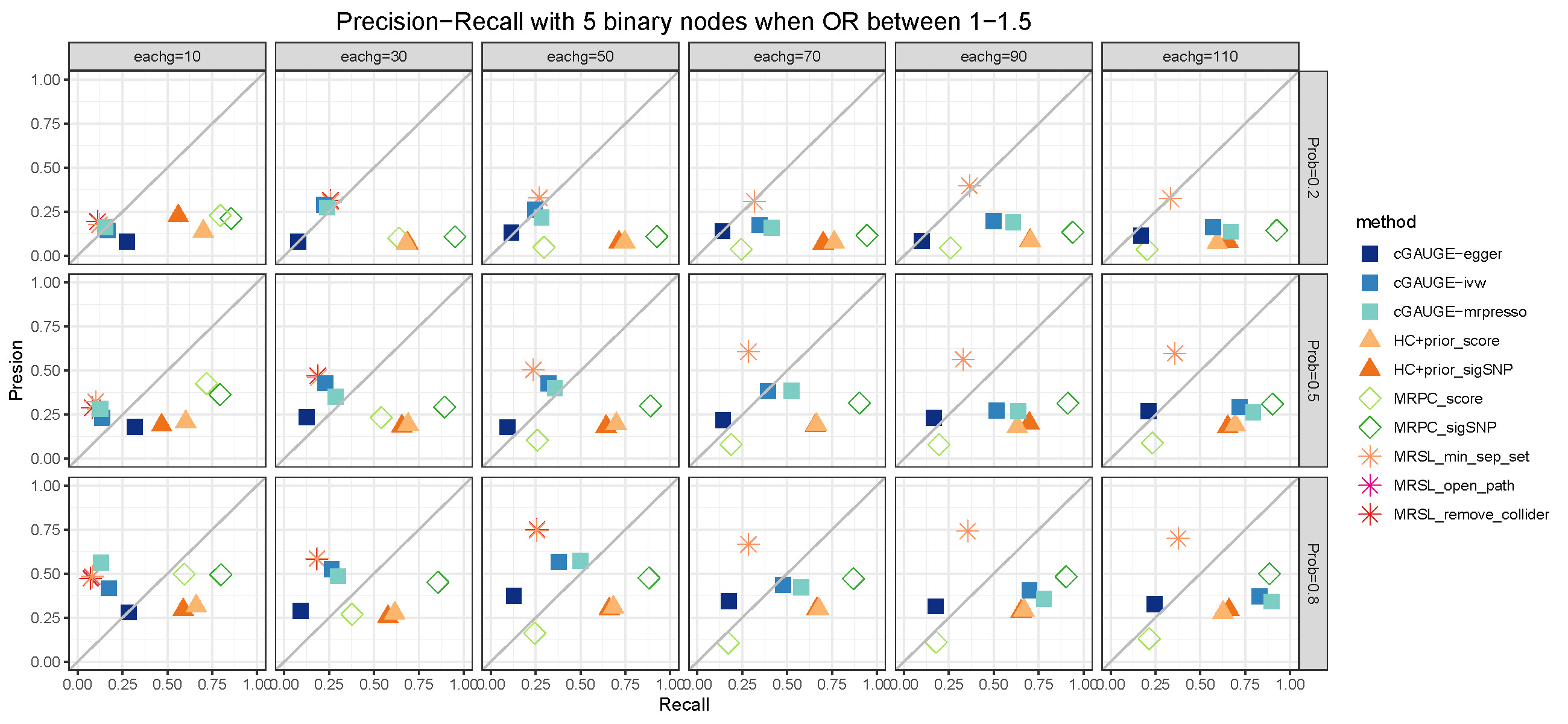


# Figure S20. Precision−Recall with 5 binary nodes when OR between 1−1.5 in simulation study 2


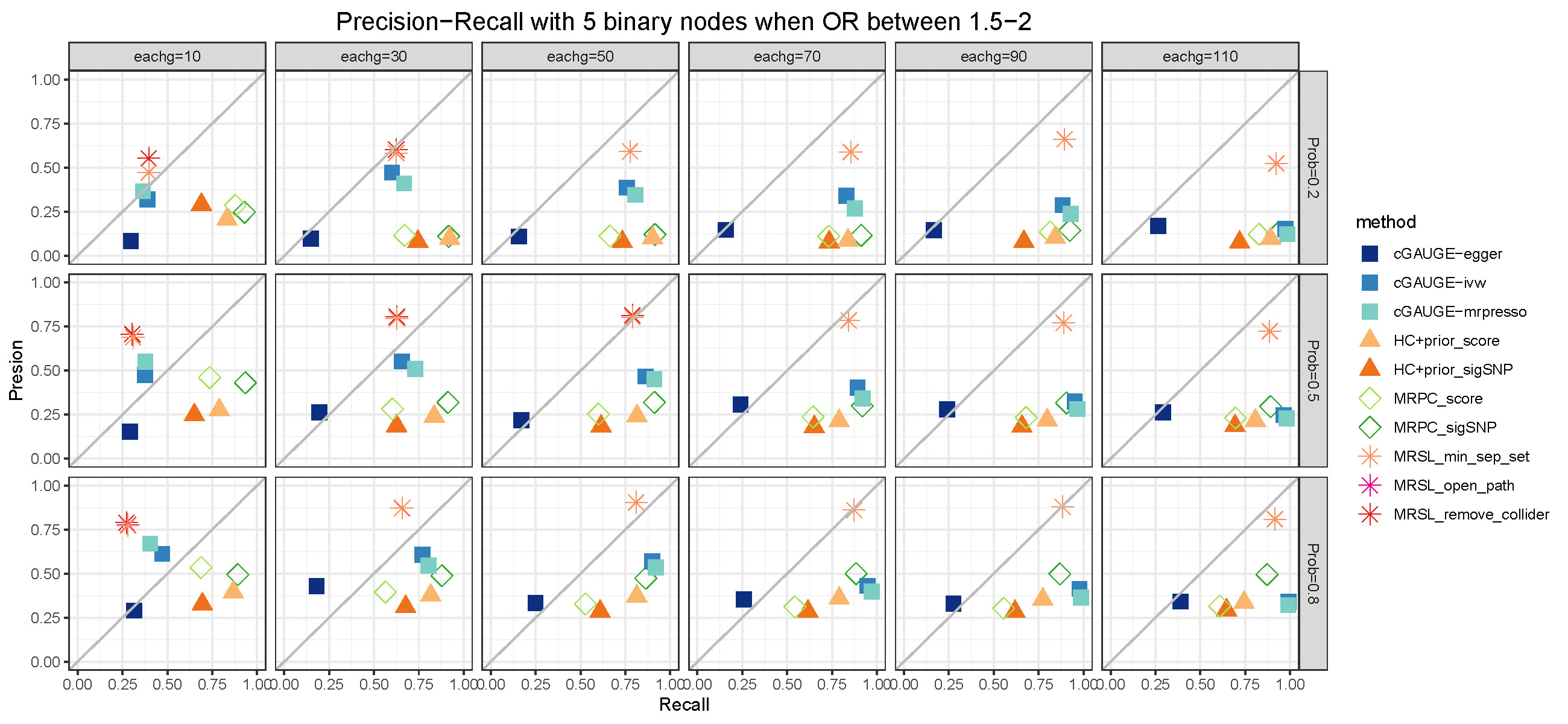


# Figure S21. Precision−Recall with 5 binary nodes when OR between 1.5−2 in simulation study 2


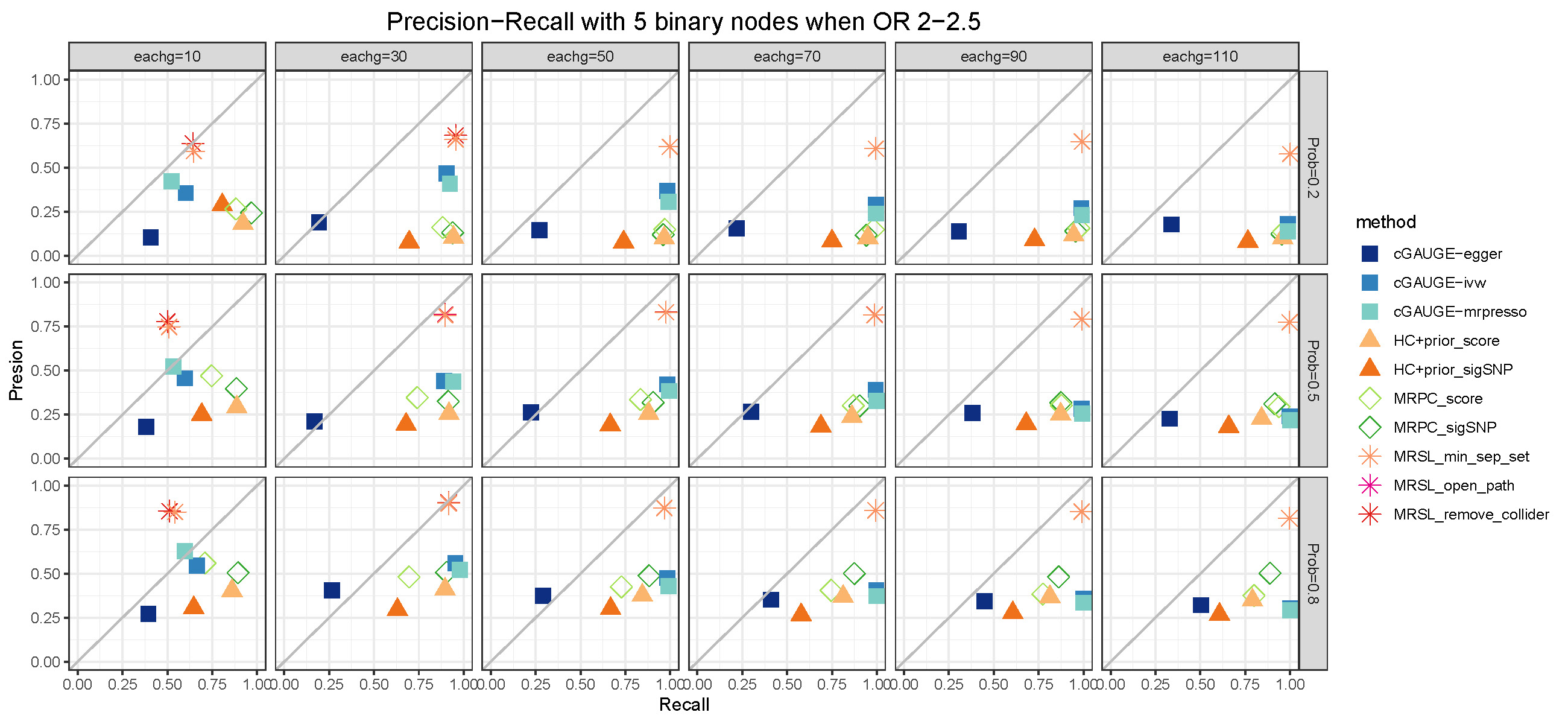


# Figure S22. Precision−Recall with 5 binary nodes when OR between 2−2.5 in simulation study 2


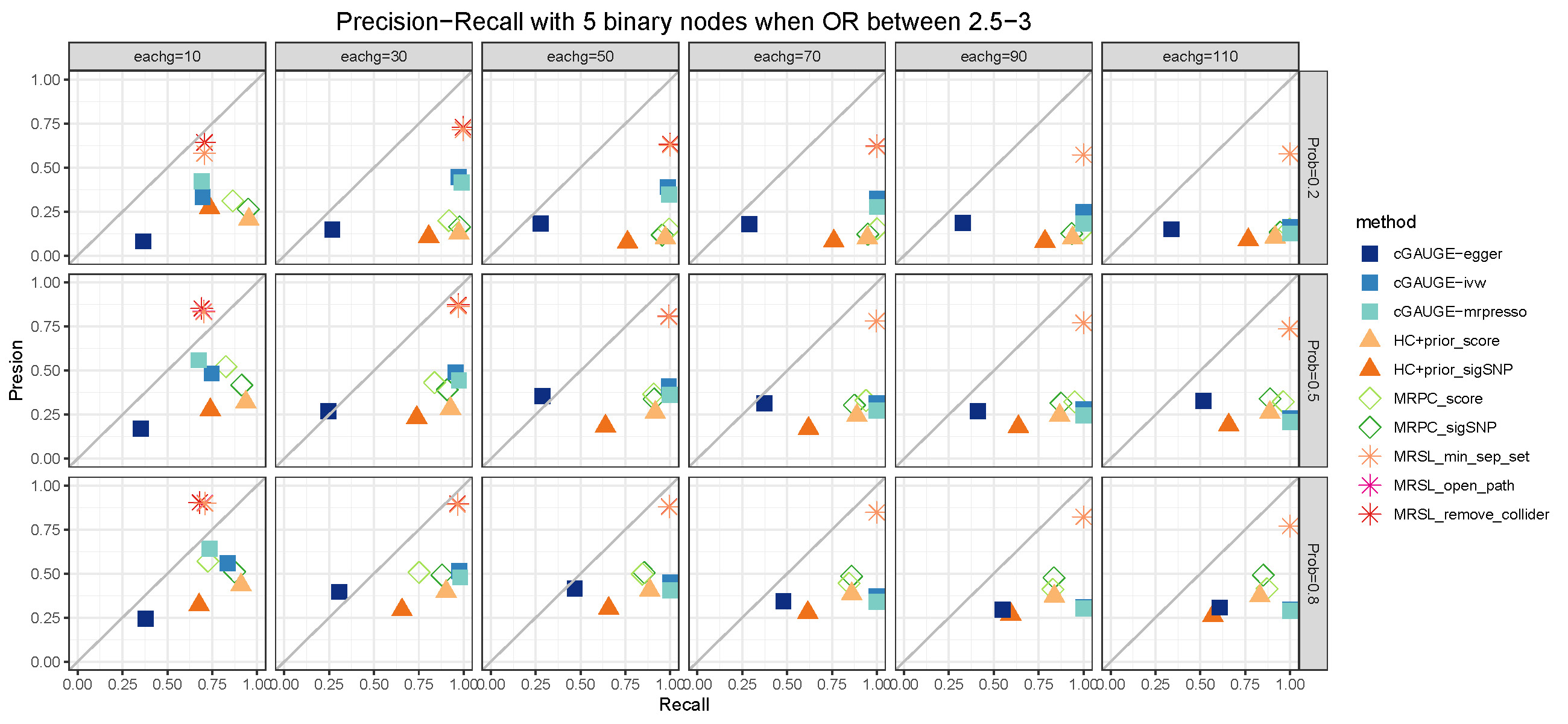


# Figure S23. Precision−Recall with 5 binary nodes when OR between 2.5−3 in simulation study 2


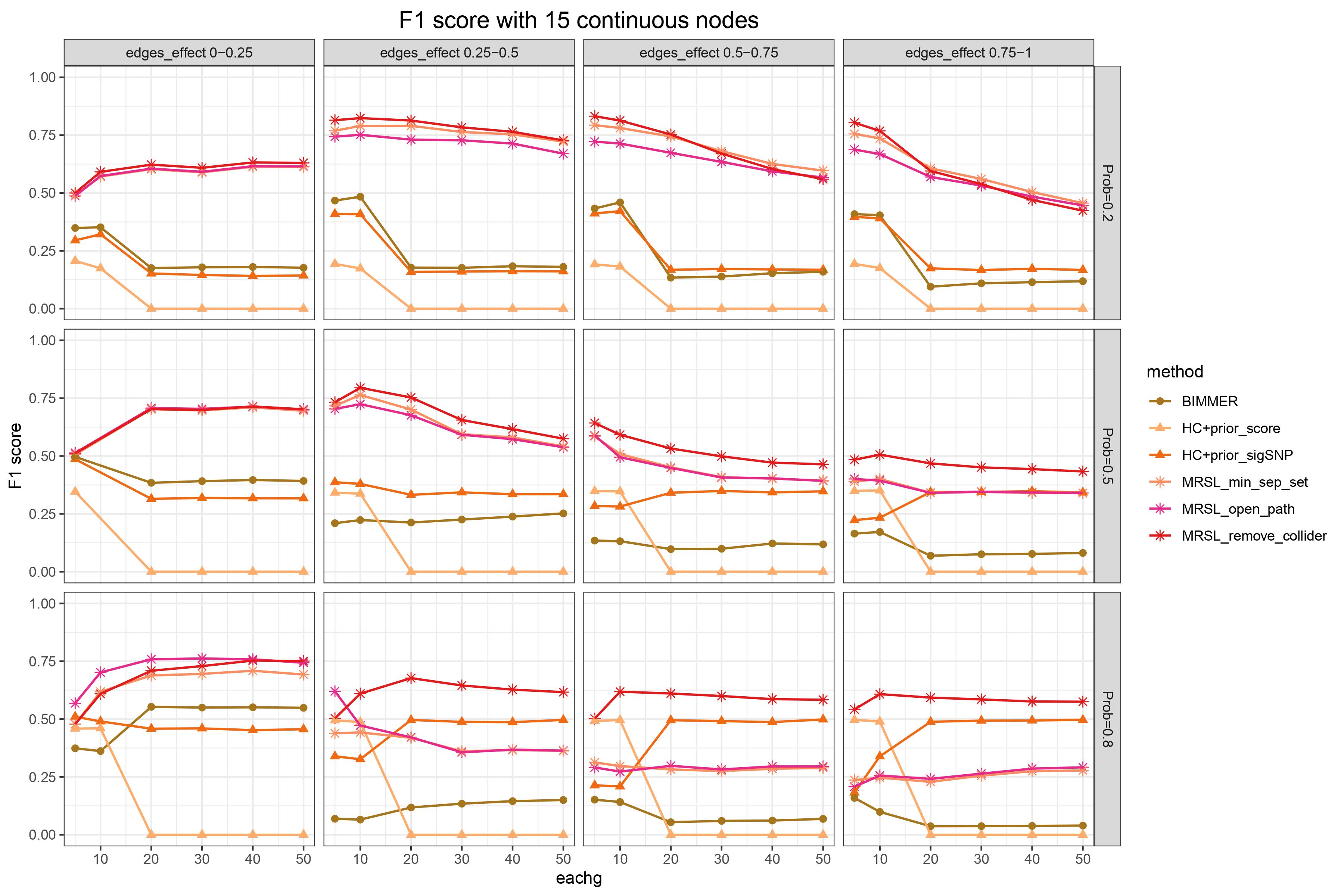


# Figure S24. F1 score with 15 continuous nodes in simulation study 2

MRPC and cGAUGE are not listed due to their huge time consuming.


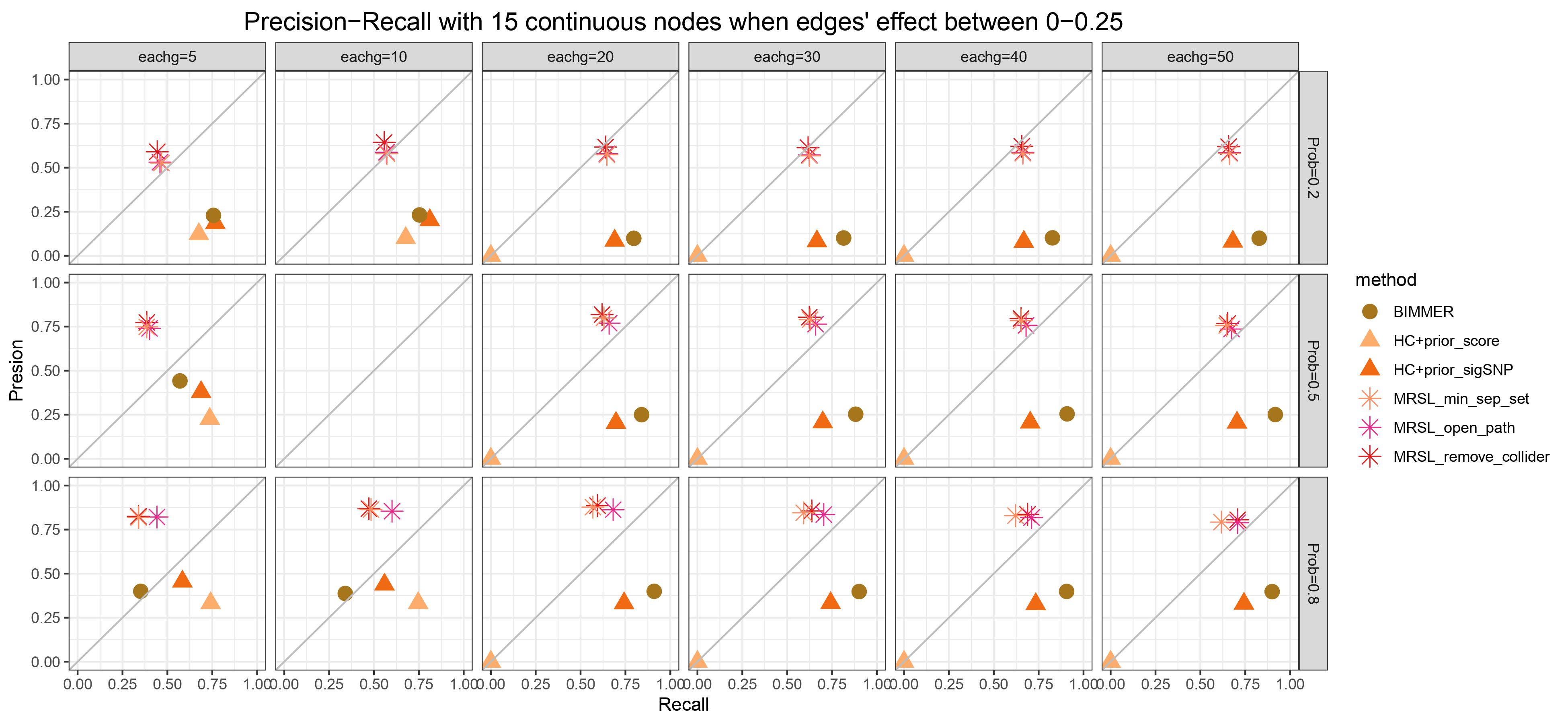


# Figure S25. Precision−Recall with 15 continuous nodes when edges' effect between 0−0.25 in simulation study 2

MRPC and cGAUGE are not listed due to their huge time consuming.


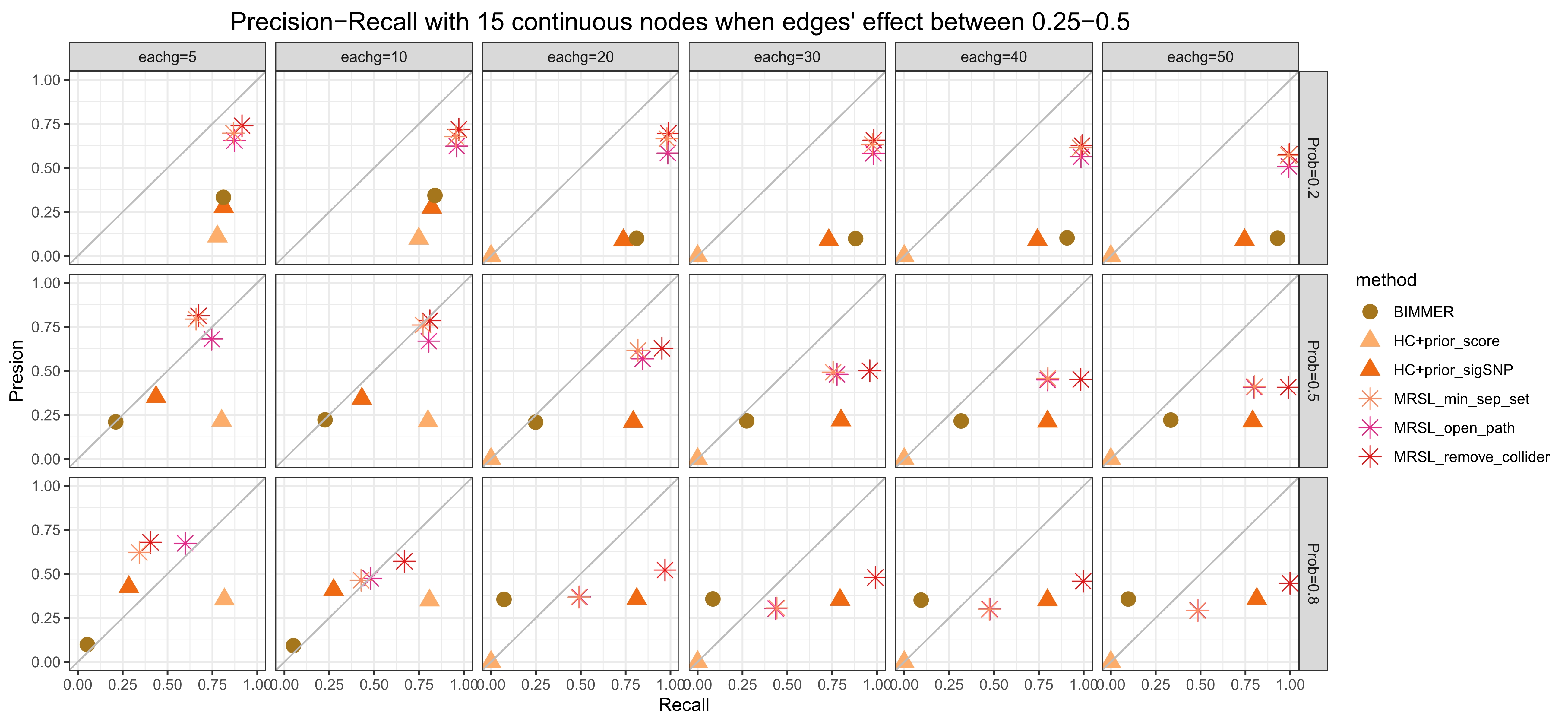


# Figure S26. Precision−Recall with 15 continuous nodes when edges' effect between 0.25−0.5 in simulation study 2

MRPC and cGAUGE are not listed due to their huge time consuming.


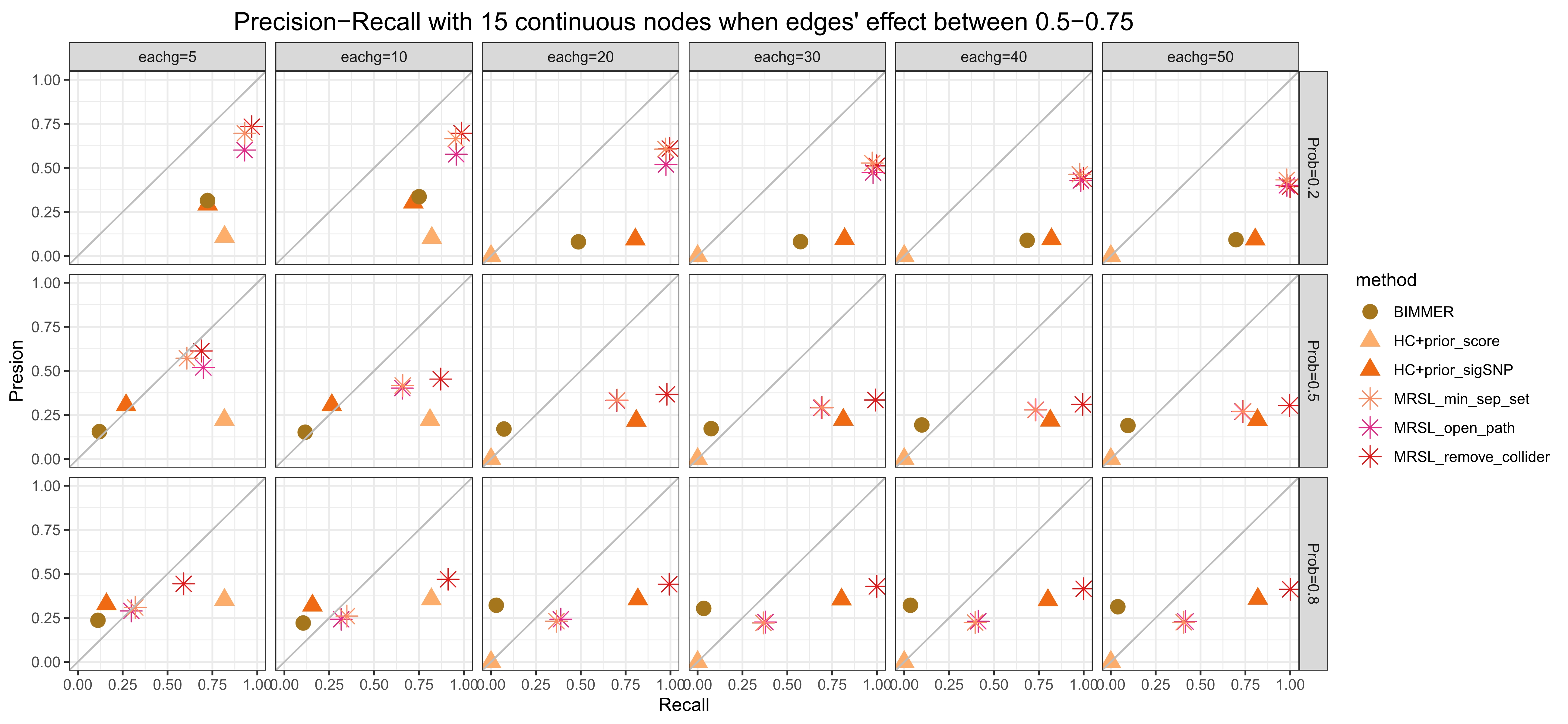


# Figure S27. Precision−Recall with 15 continuous nodes when edges' effect between 0.5−0.75 in simulation study 2

MRPC and cGAUGE are not listed due to their huge time consuming.


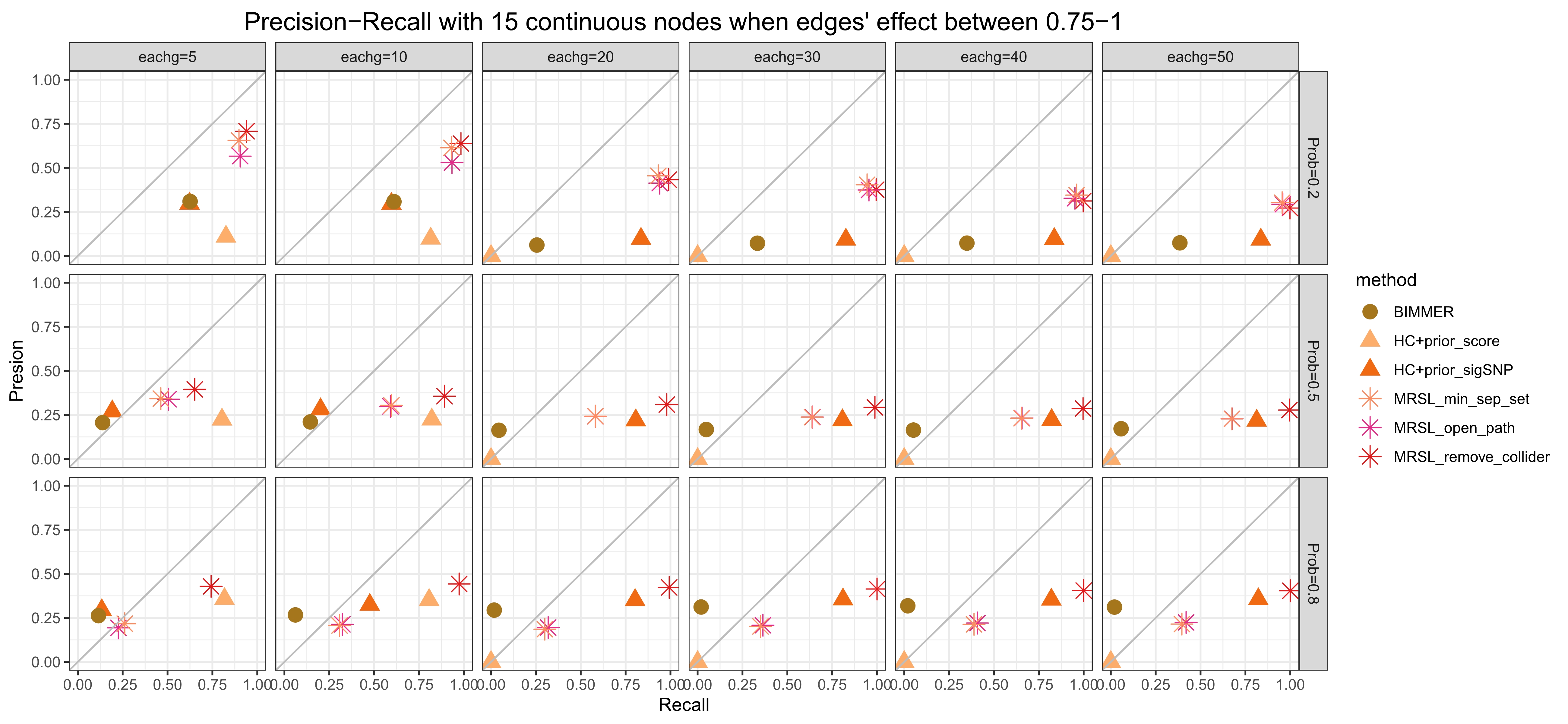


# Figure S28. Precision−Recall with 15 continuous nodes when edges' effect between 0.75−1 in simulation study 2

MRPC and cGAUGE are not listed due to their huge time consuming.


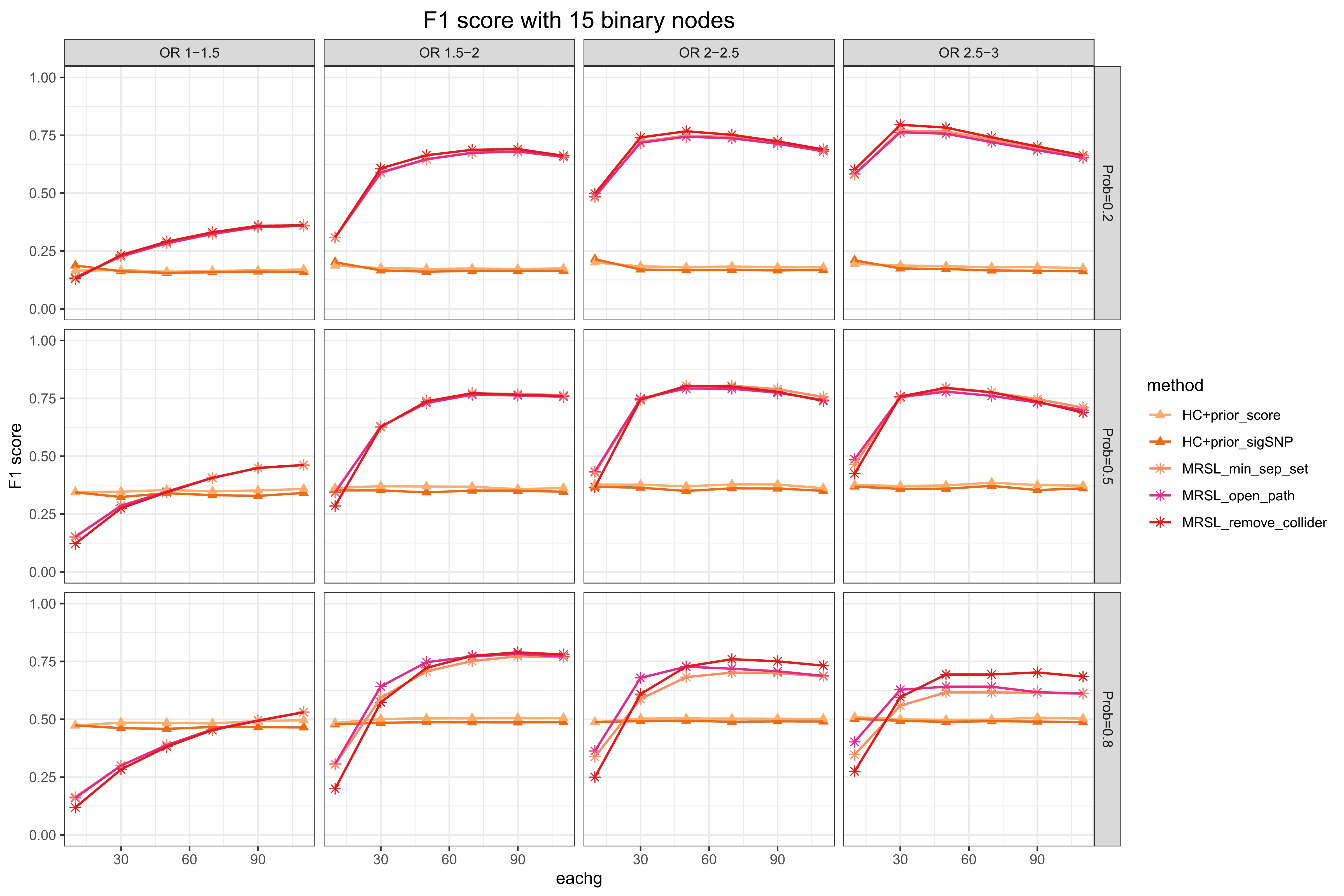


# Figure S29. F1 score with 15 binary nodes in simulation study 2

MRPC and cGAUGE are not listed due to their huge time consuming.


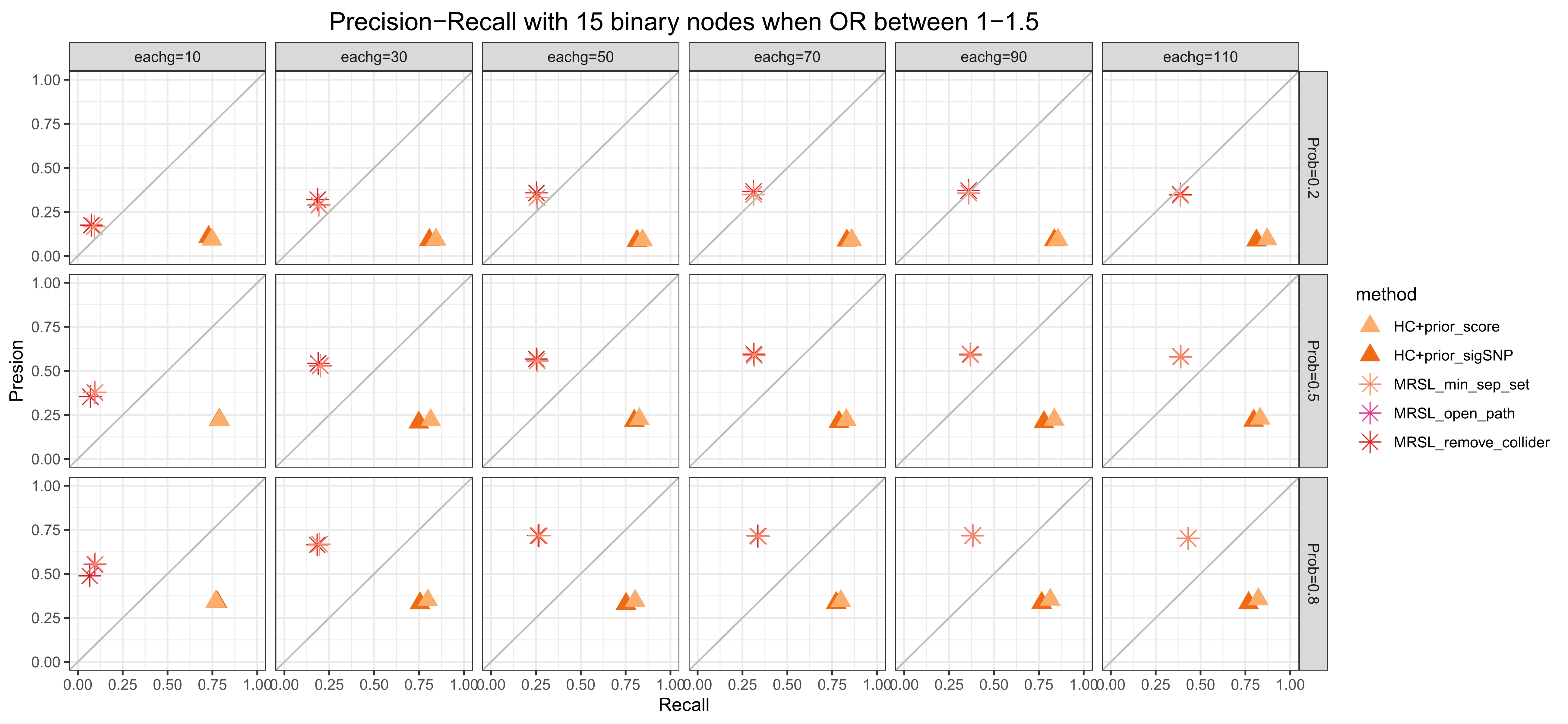


# Figure S30. Precision−Recall with 15 binary nodes when OR between 1−1.5 in simulation study 2

MRPC and cGAUGE are not listed due to their huge time consuming.


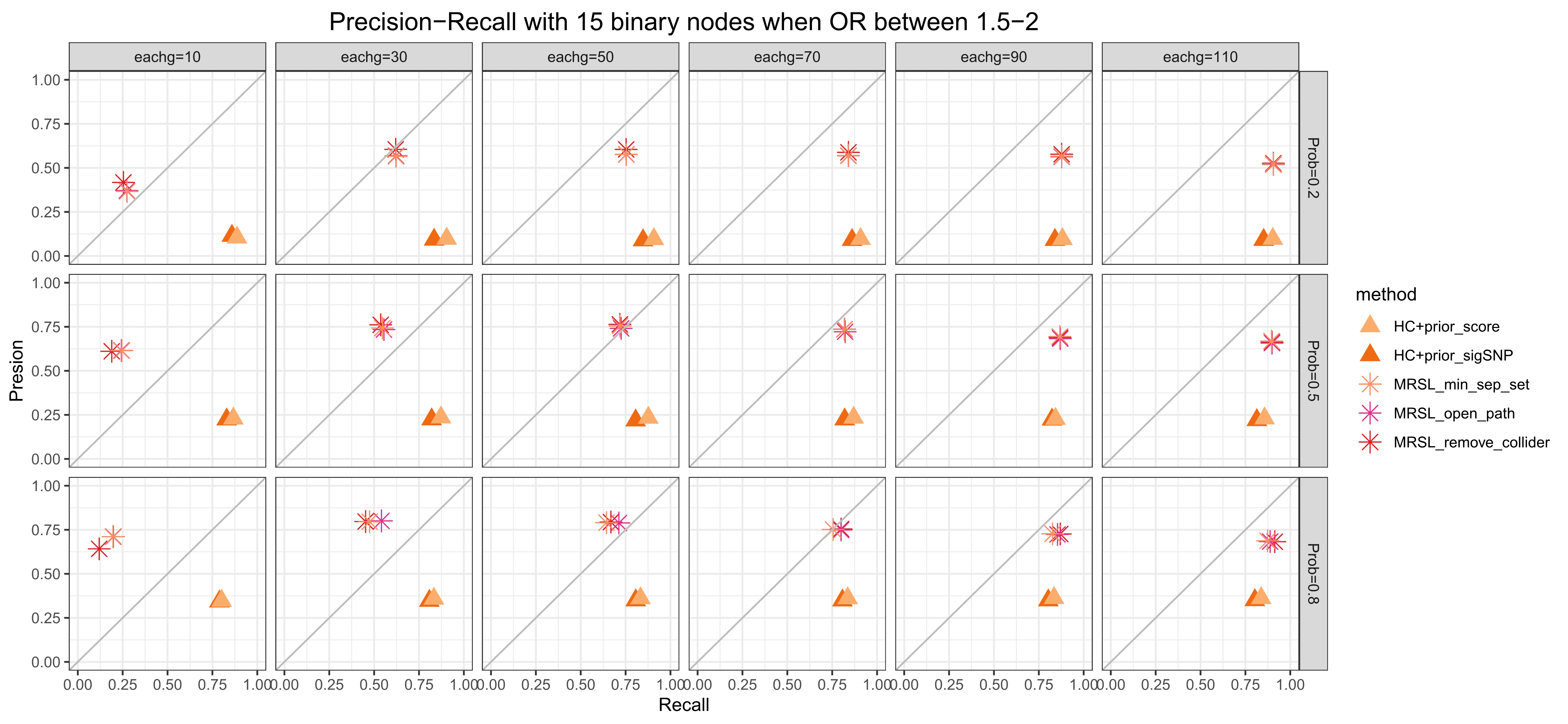


# Figure S31. Precision−Recall with 15 binary nodes when OR between 1.5−2 in simulation study 2

MRPC and cGAUGE are not listed due to their huge time consuming.


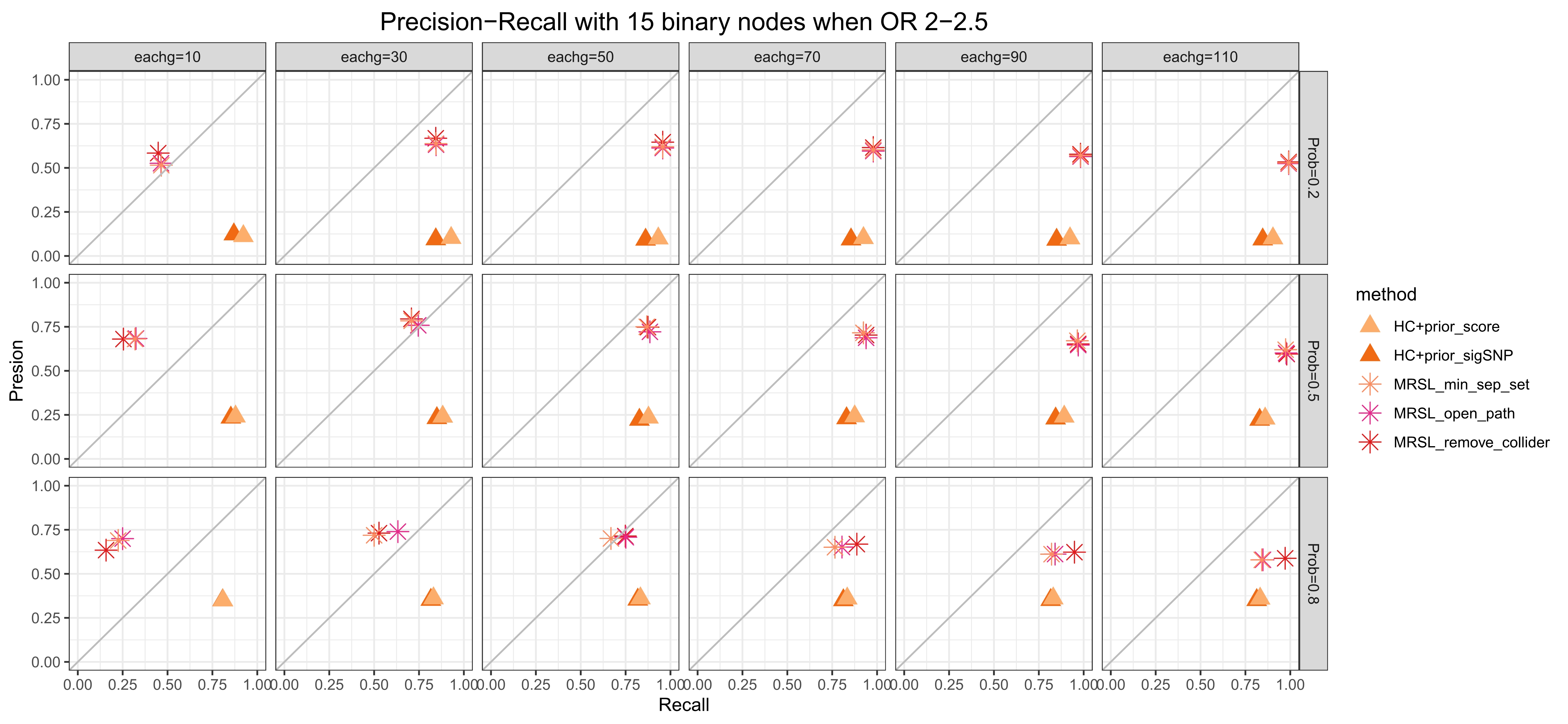


# Figure S32. Precision−Recall with 15 binary nodes when OR between 2−2.5 in simulation study 2

MRPC and cGAUGE are not listed due to their huge time consuming.


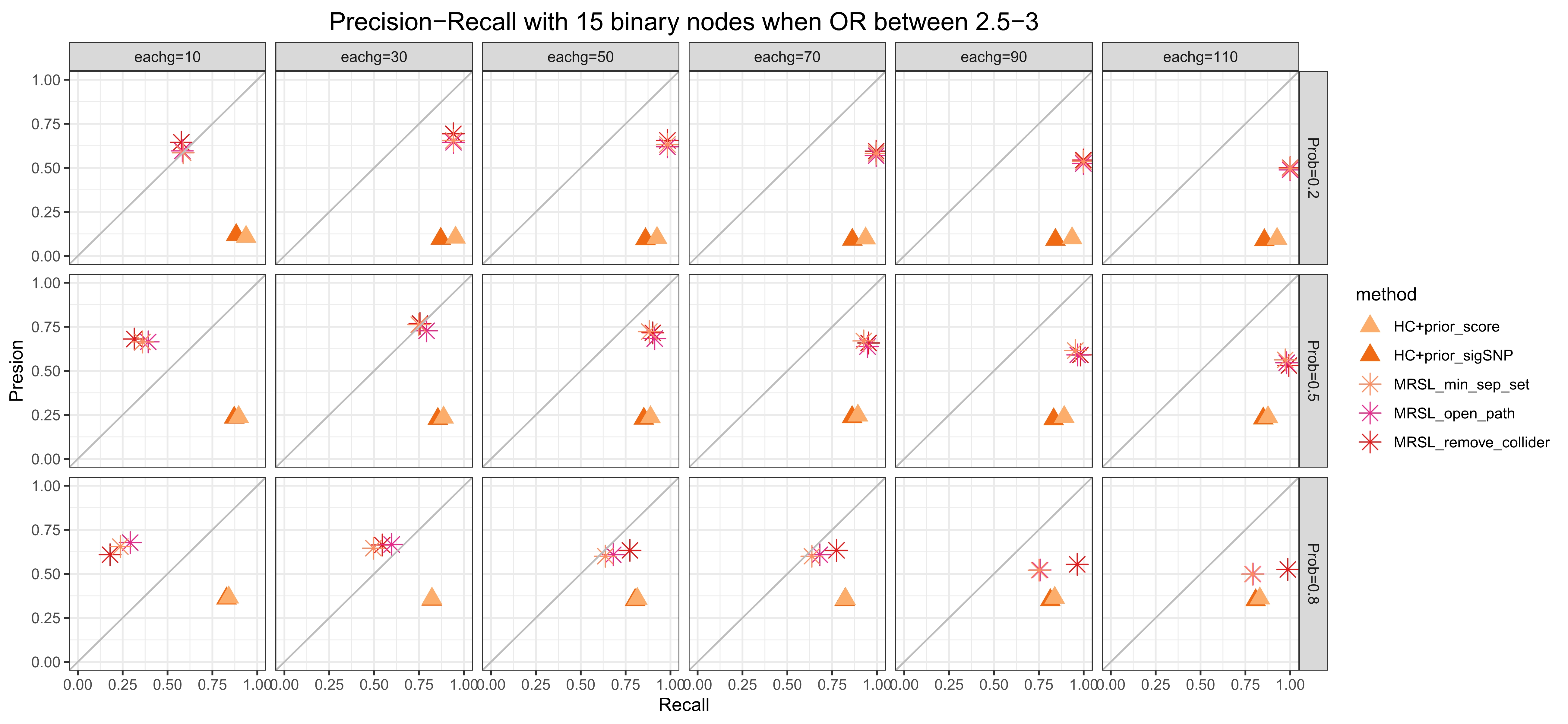


# Figure S33. Precision−Recall with 15 binary nodes when OR between 2.5−3 in simulation study 2

MRPC and cGAUGE are not listed due to their huge time consuming.


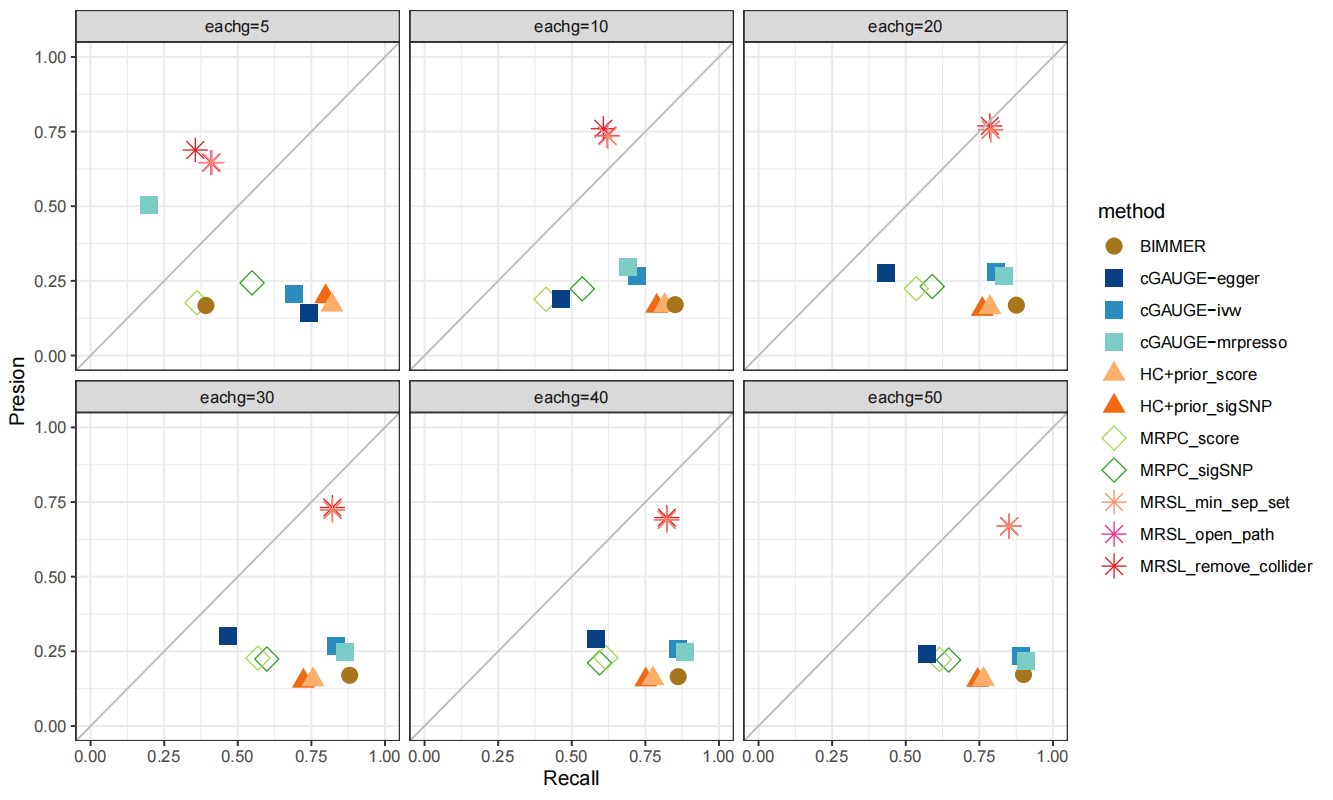


# Figure S34. Precision−Recall with Gene regulatory graph when edges' effect between 0−0.25 in simulation study 3


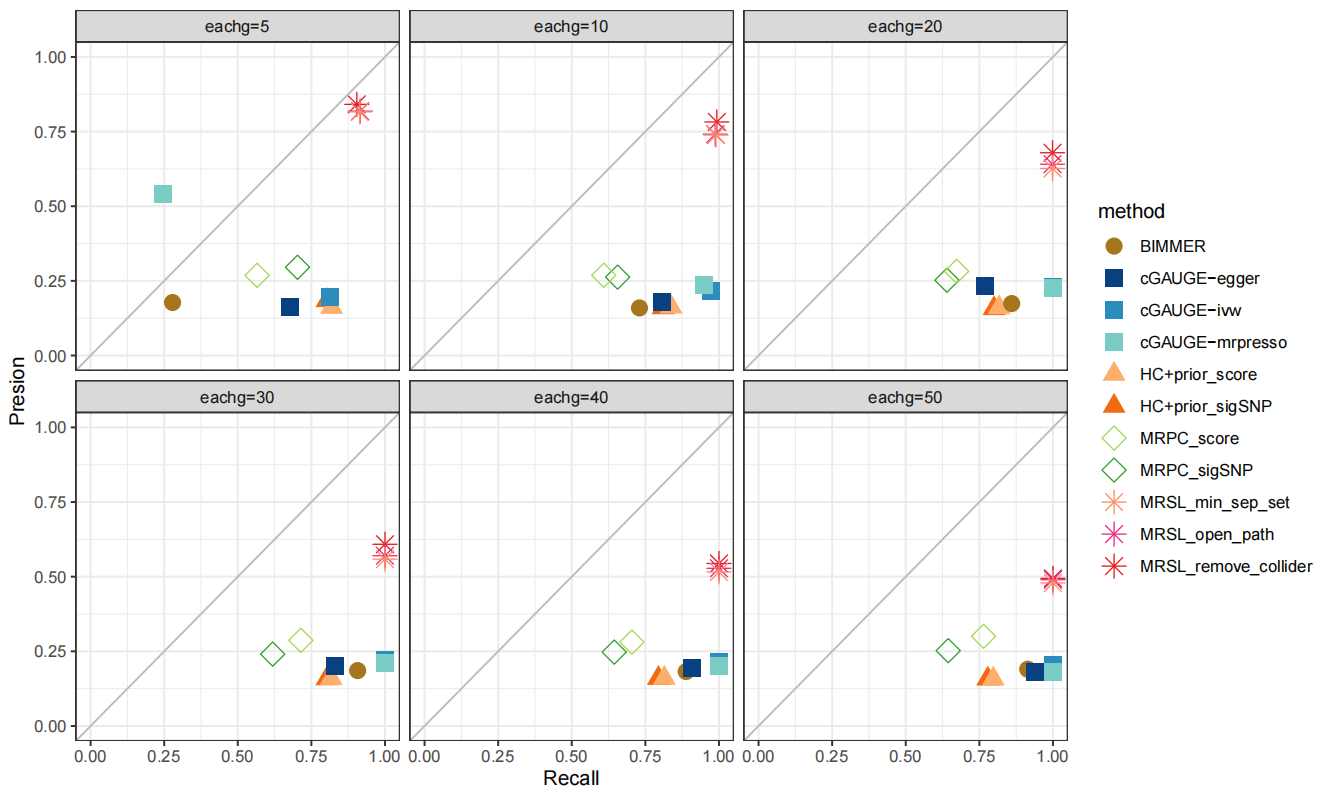


# Figure S35. Precision−Recall with Gene regulatory graph when edges' effect between 0.25−0.5 in simulation study 3


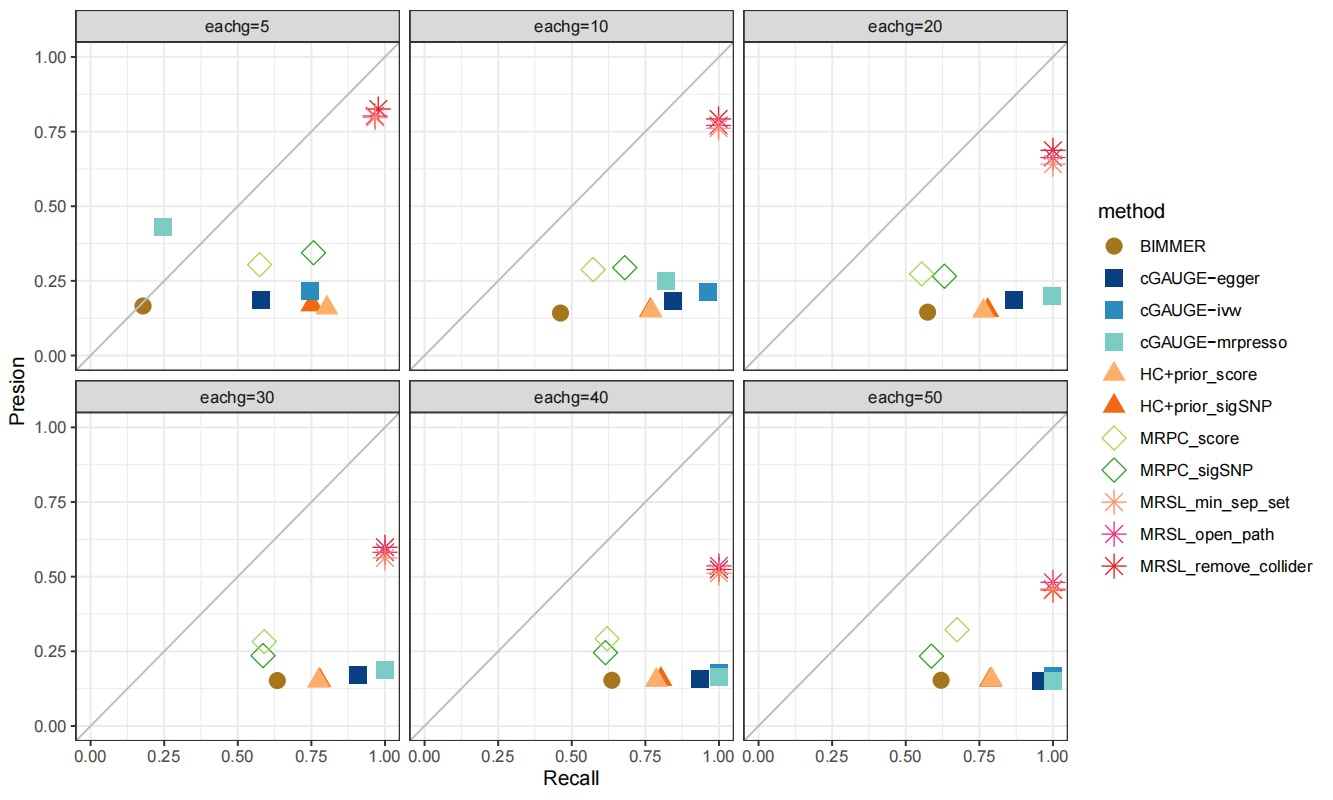


# Figure S36. Precision−Recall with Gene regulatory graph when edges' effect between 0.5−0.75 in simulation study 3


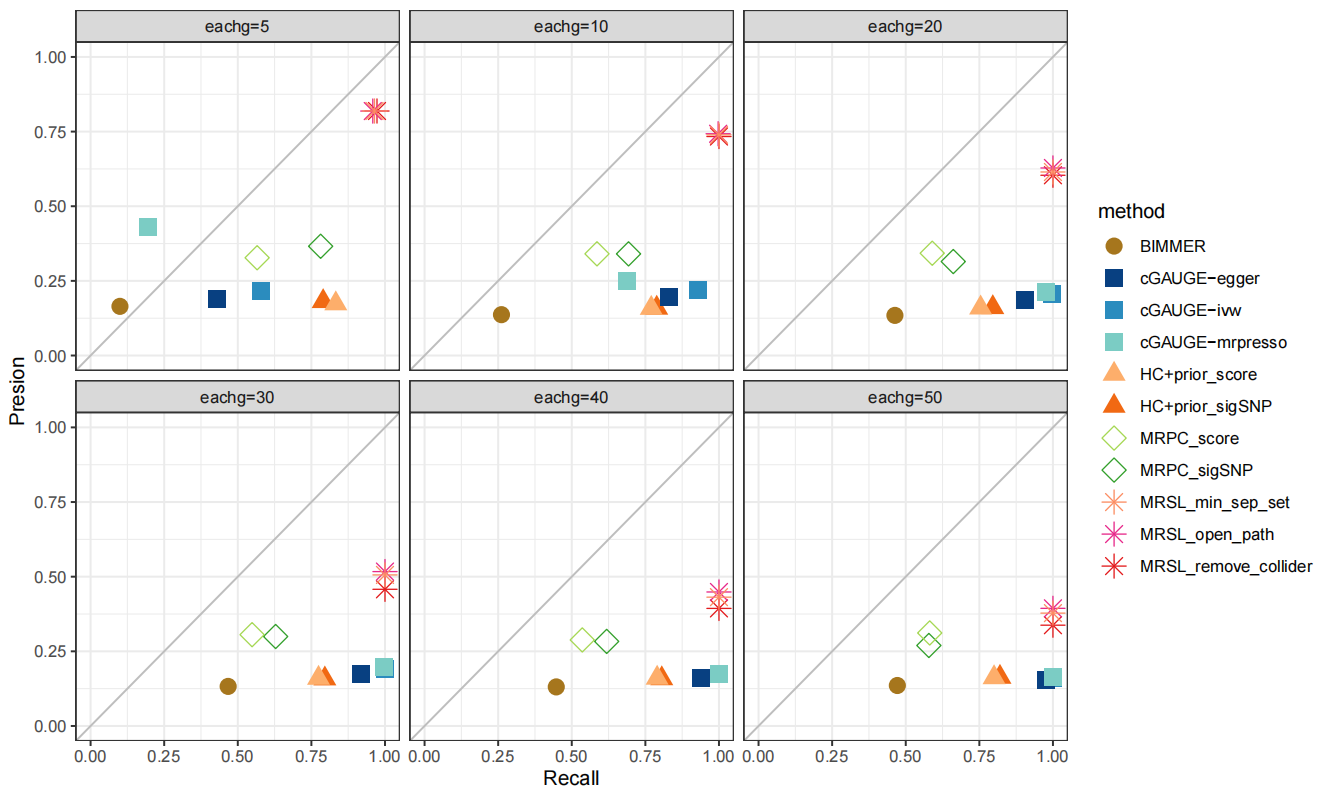


# Figure S37. Precision−Recall with Gene regulatory graph when edges' effect between 0.75−1 in simulation study 3


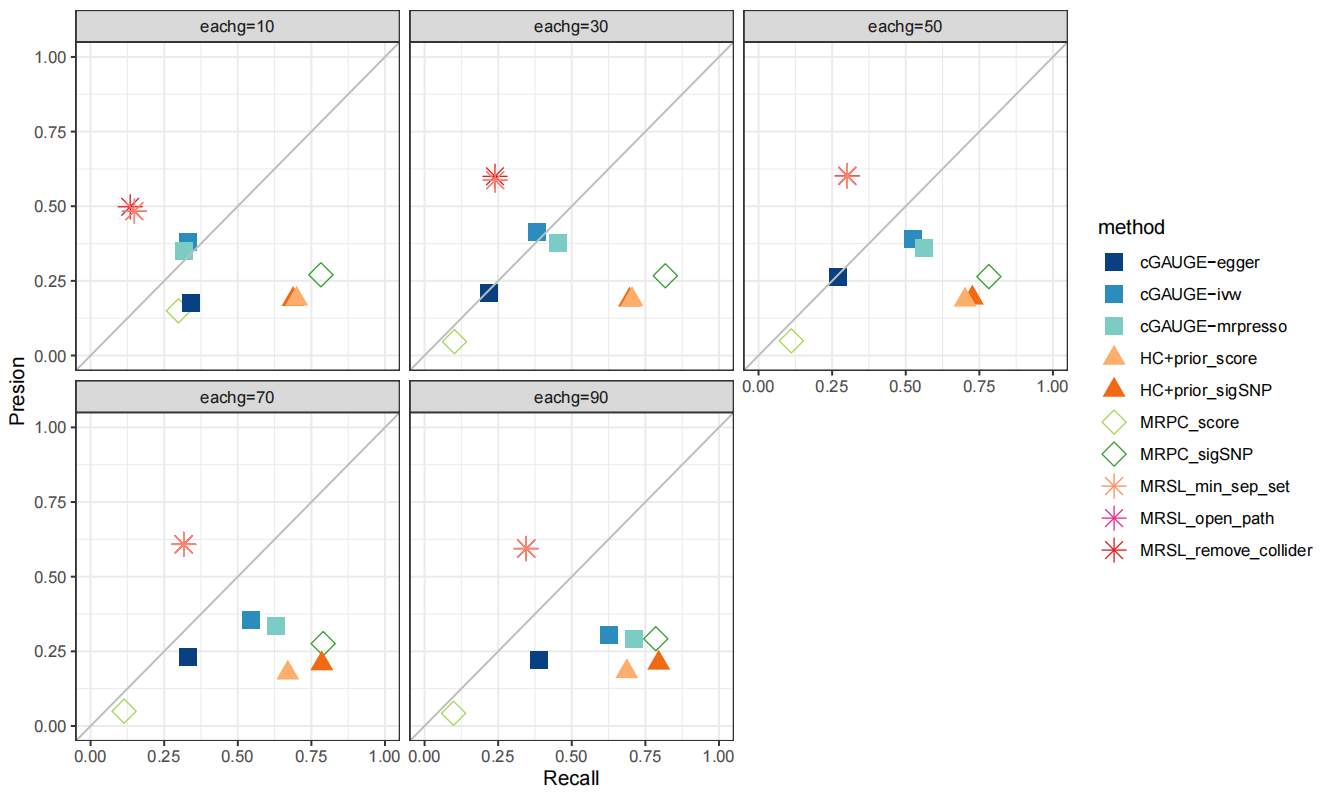


# Figure S38. Precision−Recall with Protein-Signaling graph when OR between 1−1.5 in simulation study 3


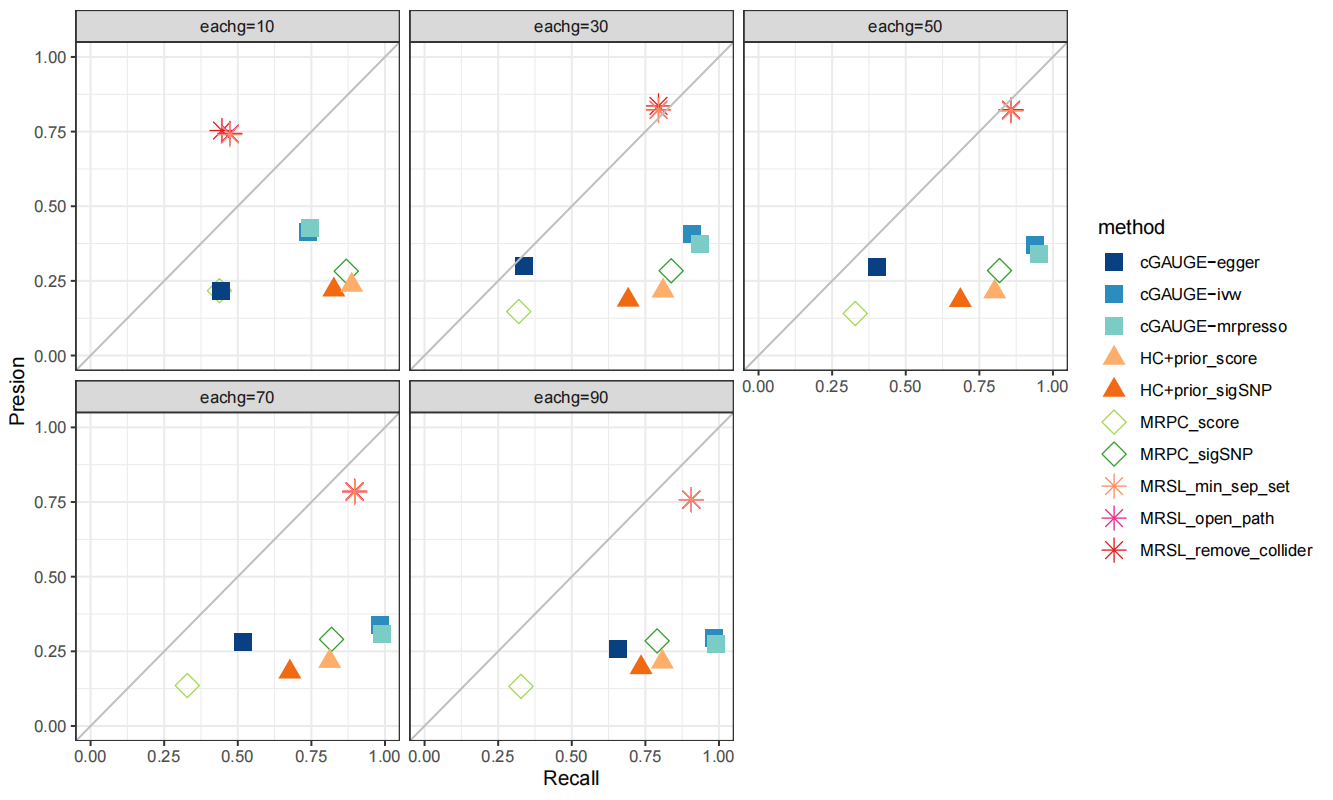


# Figure S39. Precision−Recall with Protein-Signaling graph when OR between 1.5−2 in simulation study 3


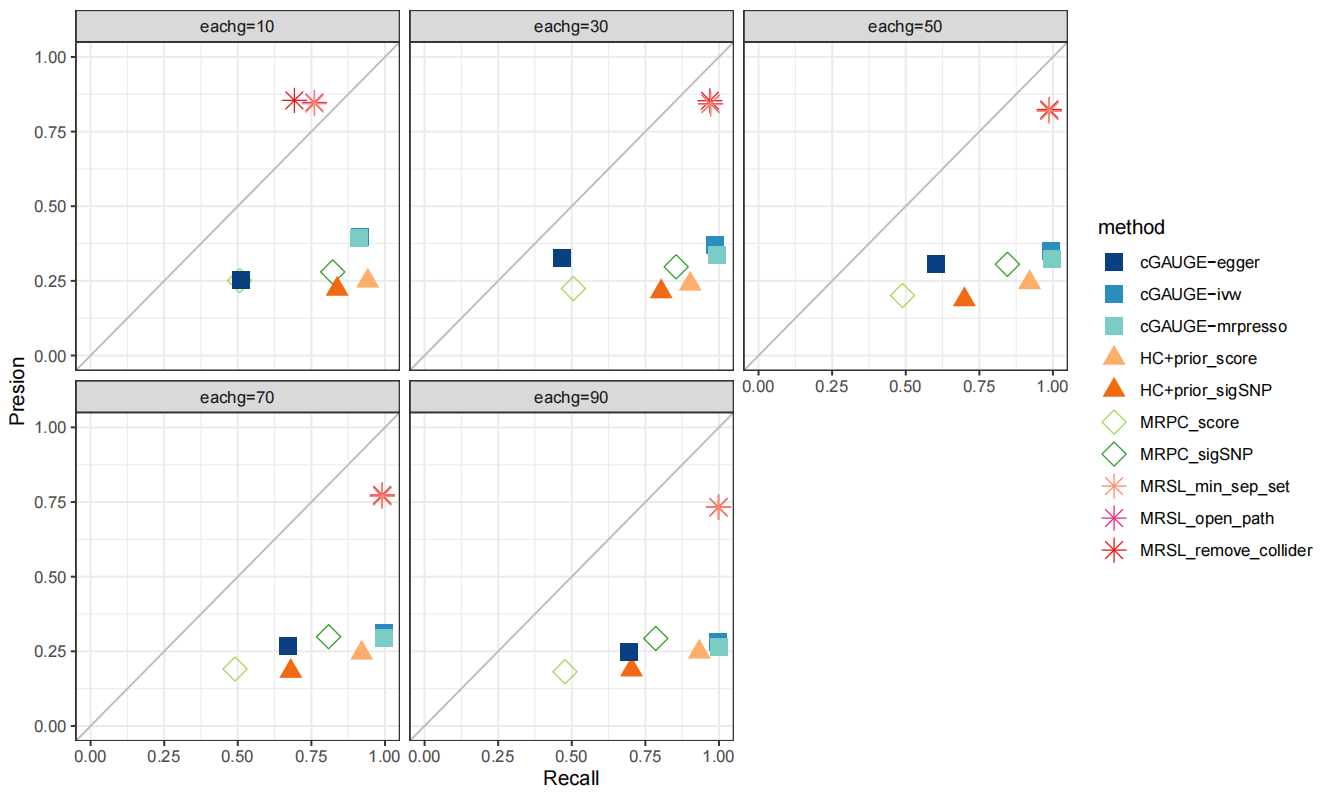


# Figure S40. Precision−Recall with Protein-Signaling graph when OR between 2−2.5 in simulation study 3


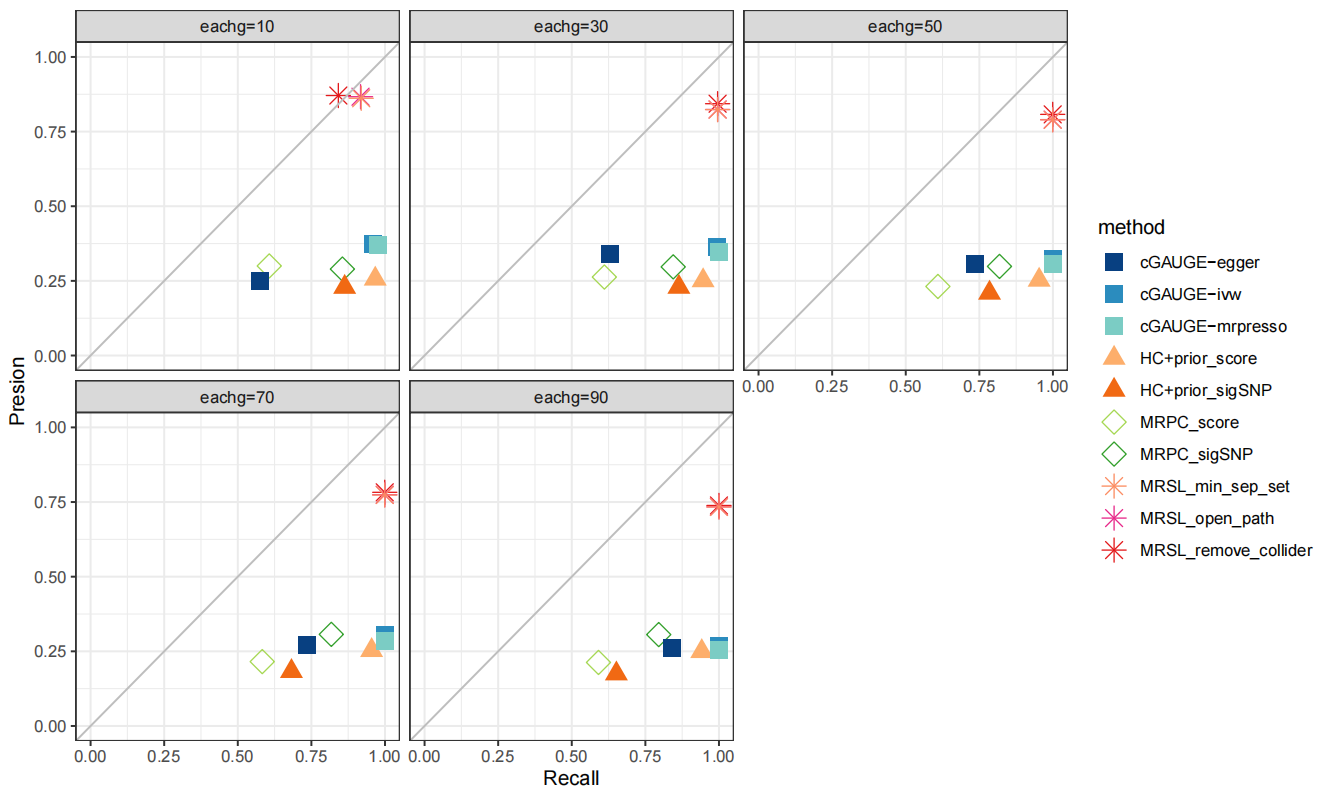


# Figure S41. Precision−Recall with Protein-Signaling graph when OR between 2.5−3 in simulation study 3


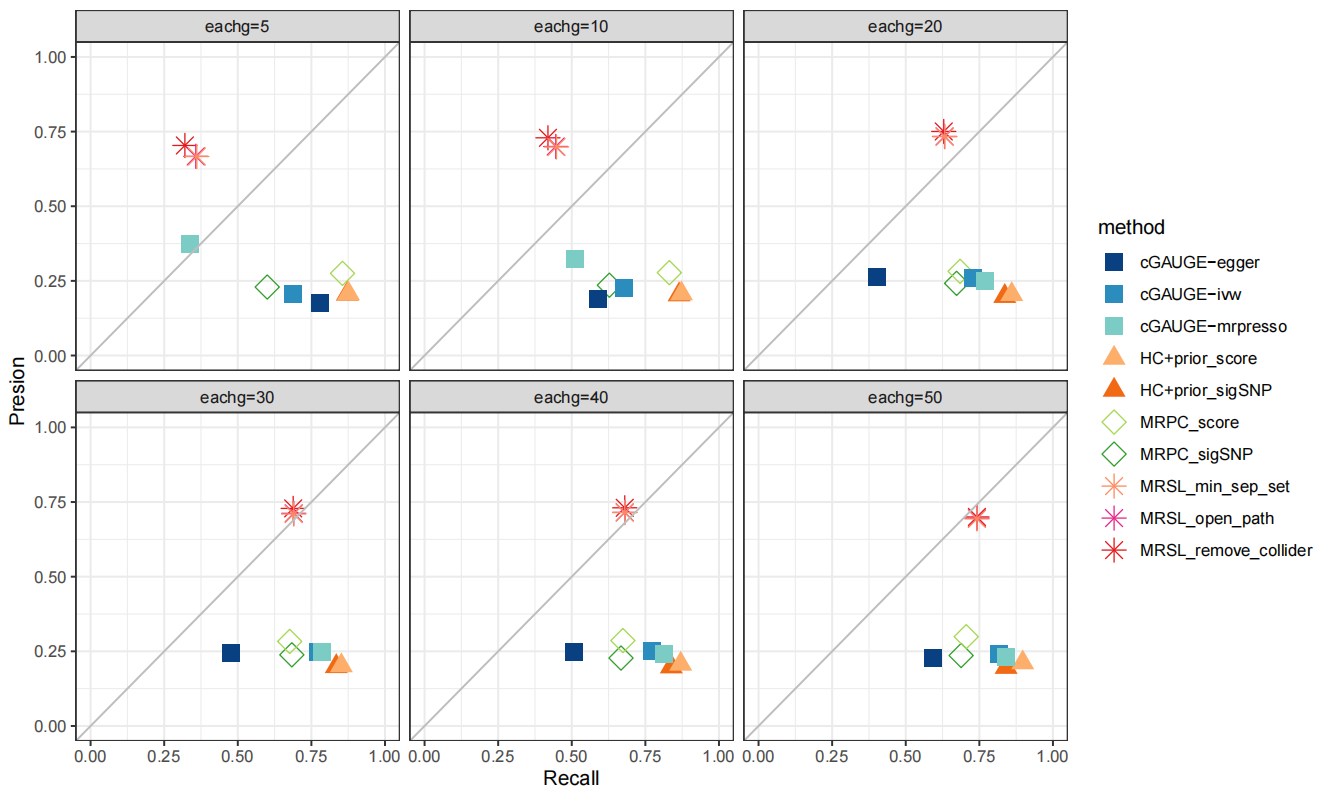


# Figure S42. Precision−Recall with Metabolic syndrome graph when edges' effect between 0−0.25


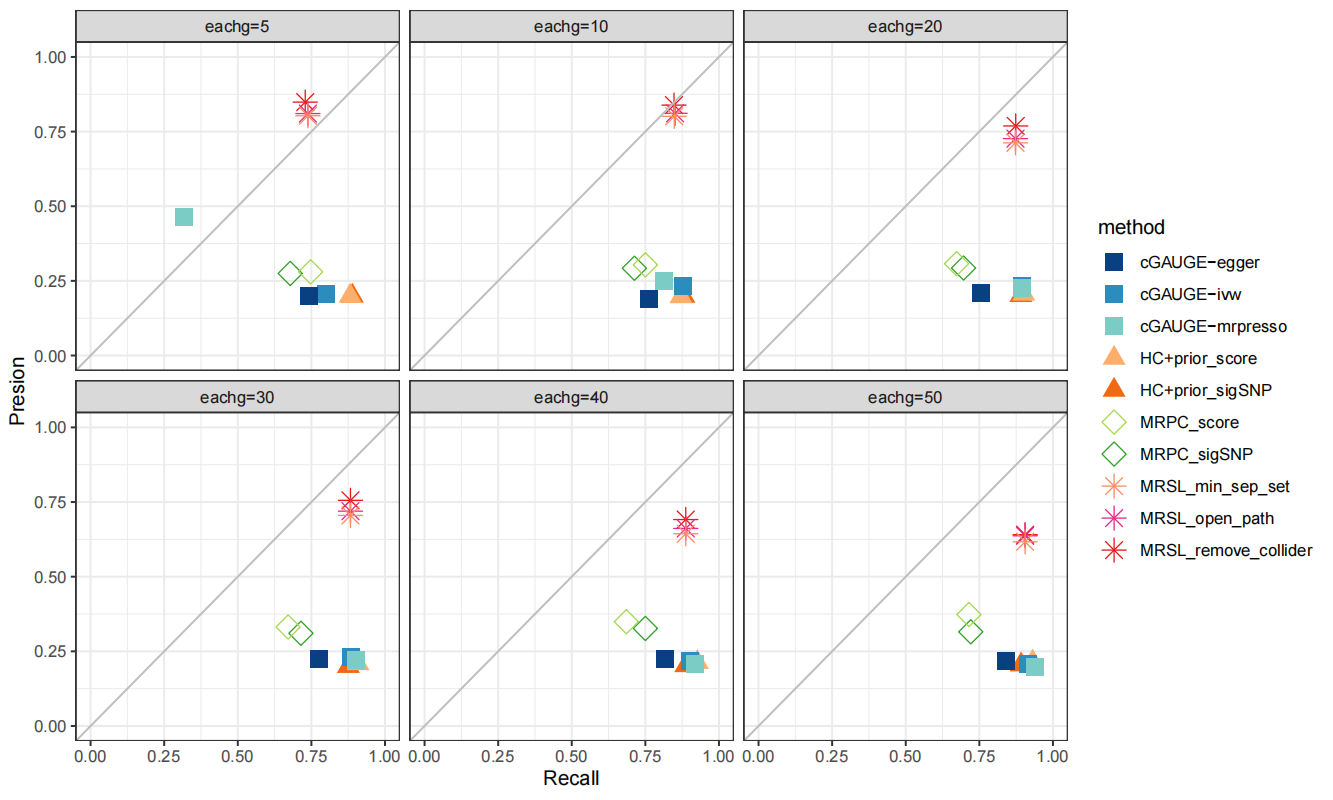


# Figure S43. Precision−Recall with Metabolic syndrome graph when edges' effect between 0.25−0.5


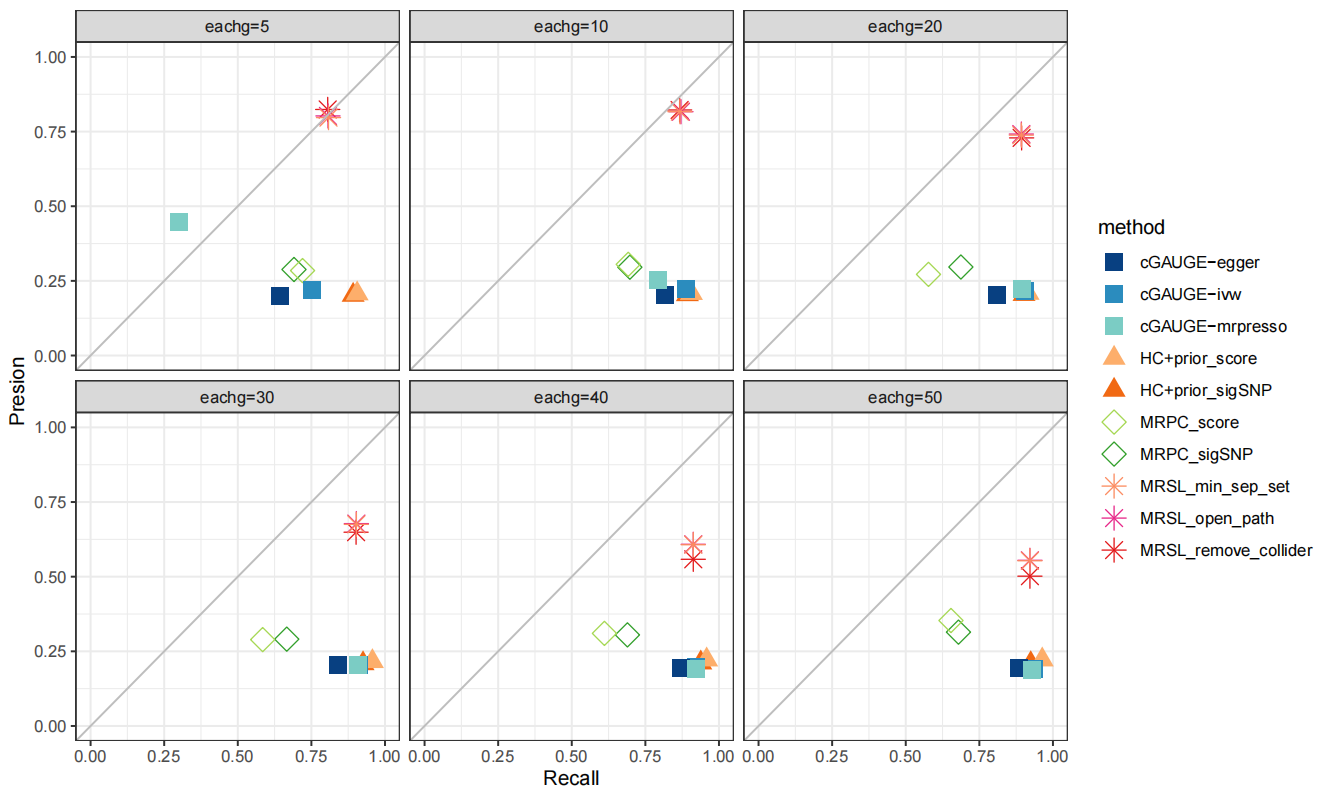


# Figure S44. Precision−Recall with Metabolic syndrome graph when edges' effect between 0.5−0.75


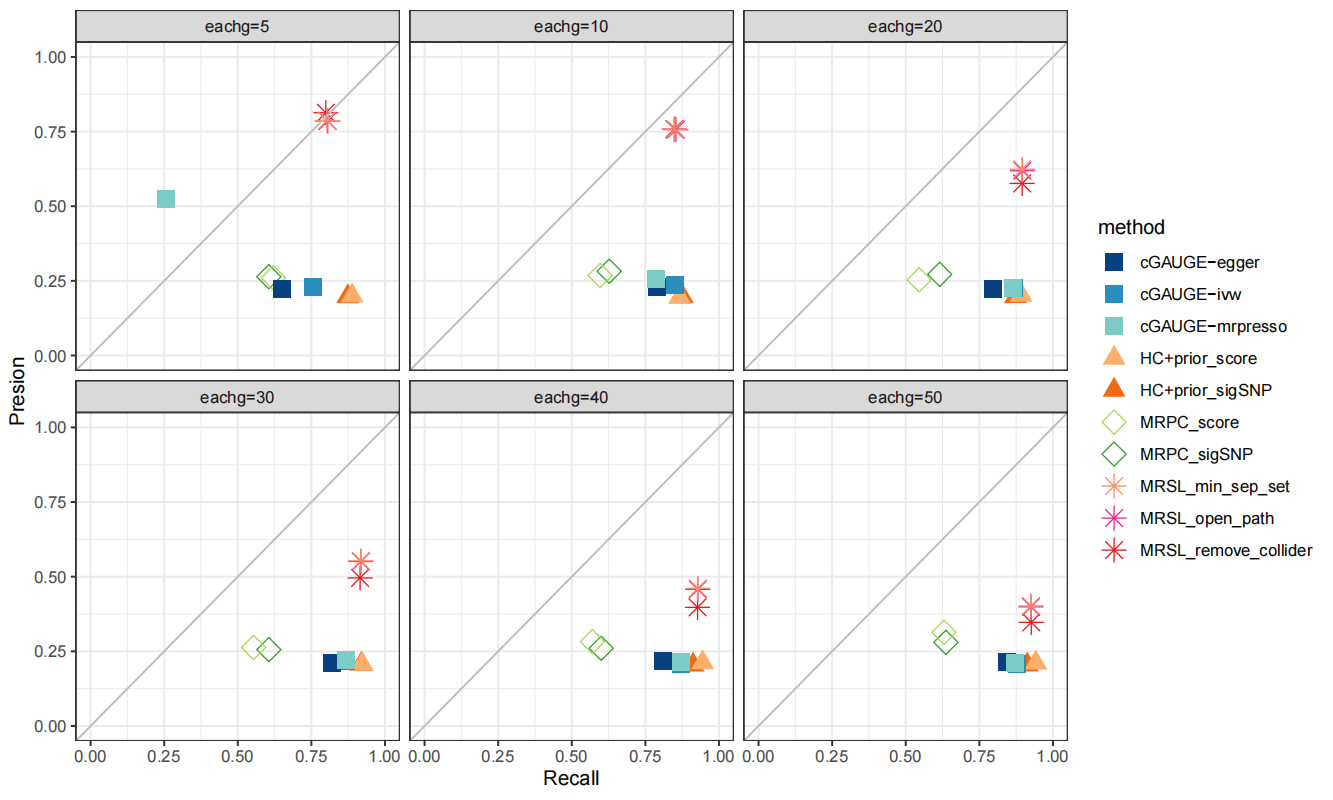


# Figure S45. Precision−Recall with Metabolic syndrome graph when edges' effect between 0.75−1


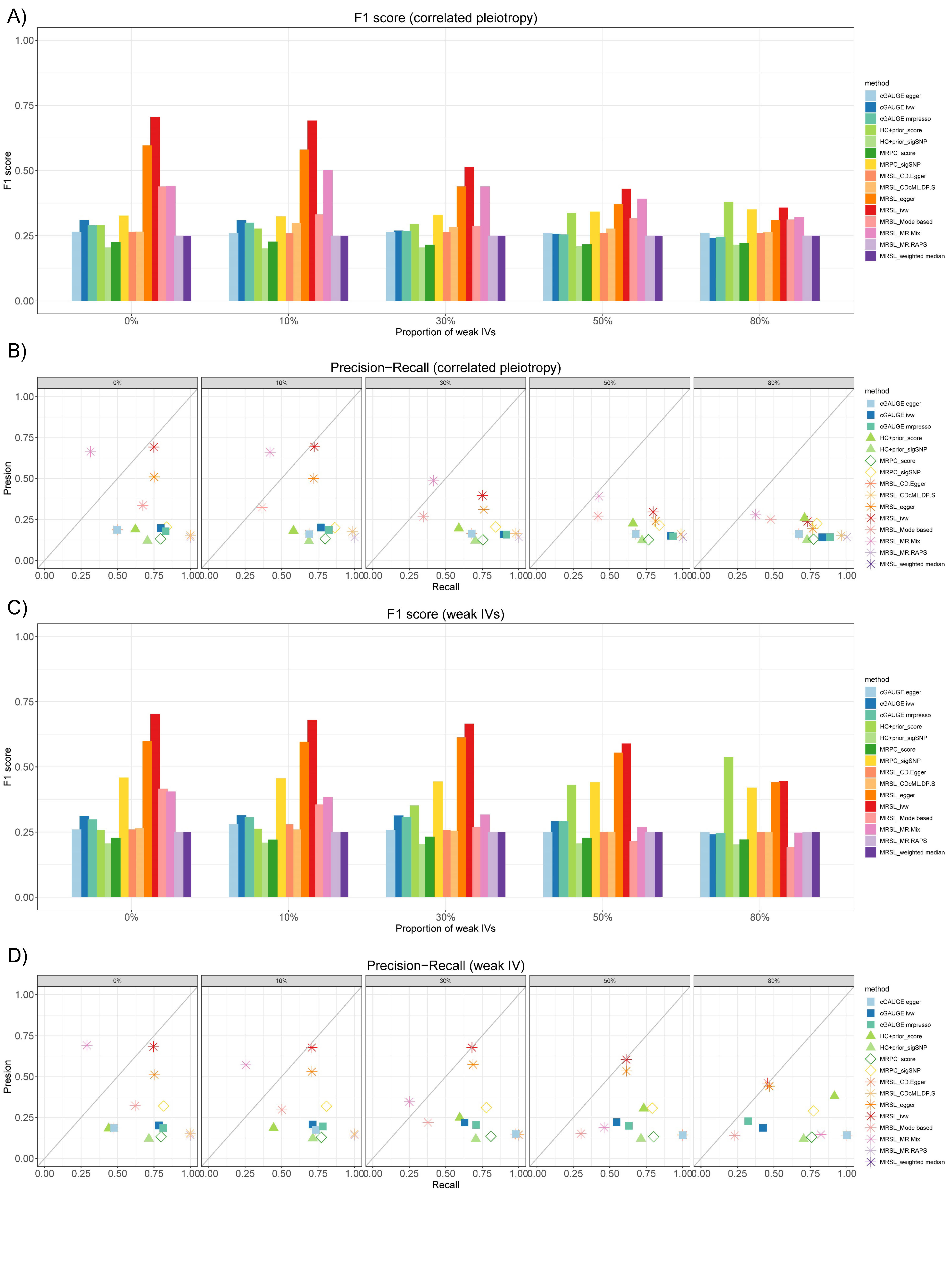


# Figure S46. F1score, precision and recall in simulation study 4 (binary variable)


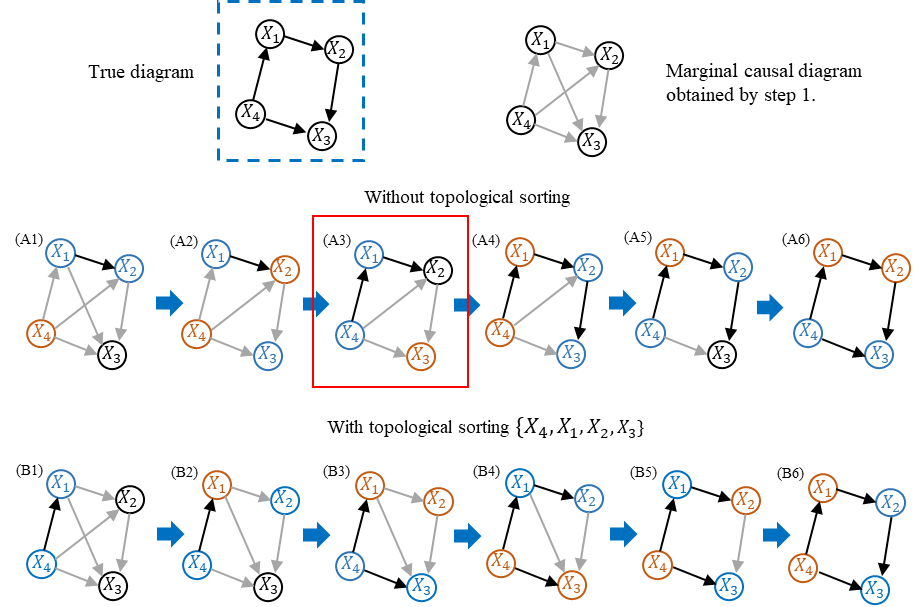


# Figure S47. Example for MRSL with and without topological sorting

The topological sorting avoids the case in Figure S46, in which we show the MVMR adjusting for the genetic associations with the phenotypes in $ADJ_{X_{p}\to X_{q}}^{(3)}$ with and without topological sorting. For the latter, when we perform MVMR to test whether the edge $X_{4}\to X_{1}$ exists, the genetic association with $X_{3}$ is adjusted in MVMR. However, in A3, $X_{3}$ is a collider but is not included in $S^{d}$, and $X_{2}$ is included in $S^{d}$. Then we perform MVMR $\hat{\beta}_{X_{1j}}\text{\textasciitilde}\alpha_{41}\hat{\beta}_{X_{4j}}+\alpha_{31}\hat{\beta}_{X_{3j}}$ and the estimation for direct effect of $X_{4}$ on $X_{1}$ is biased because $X_{3}$ is a collider. The bias formula of causal estimation when adjusting for a collider using MVMR are shown in Supplementary Notes. For the former, this kind of problem can be avoided and the process of removing edges is more accurate and faster after topological sorting.


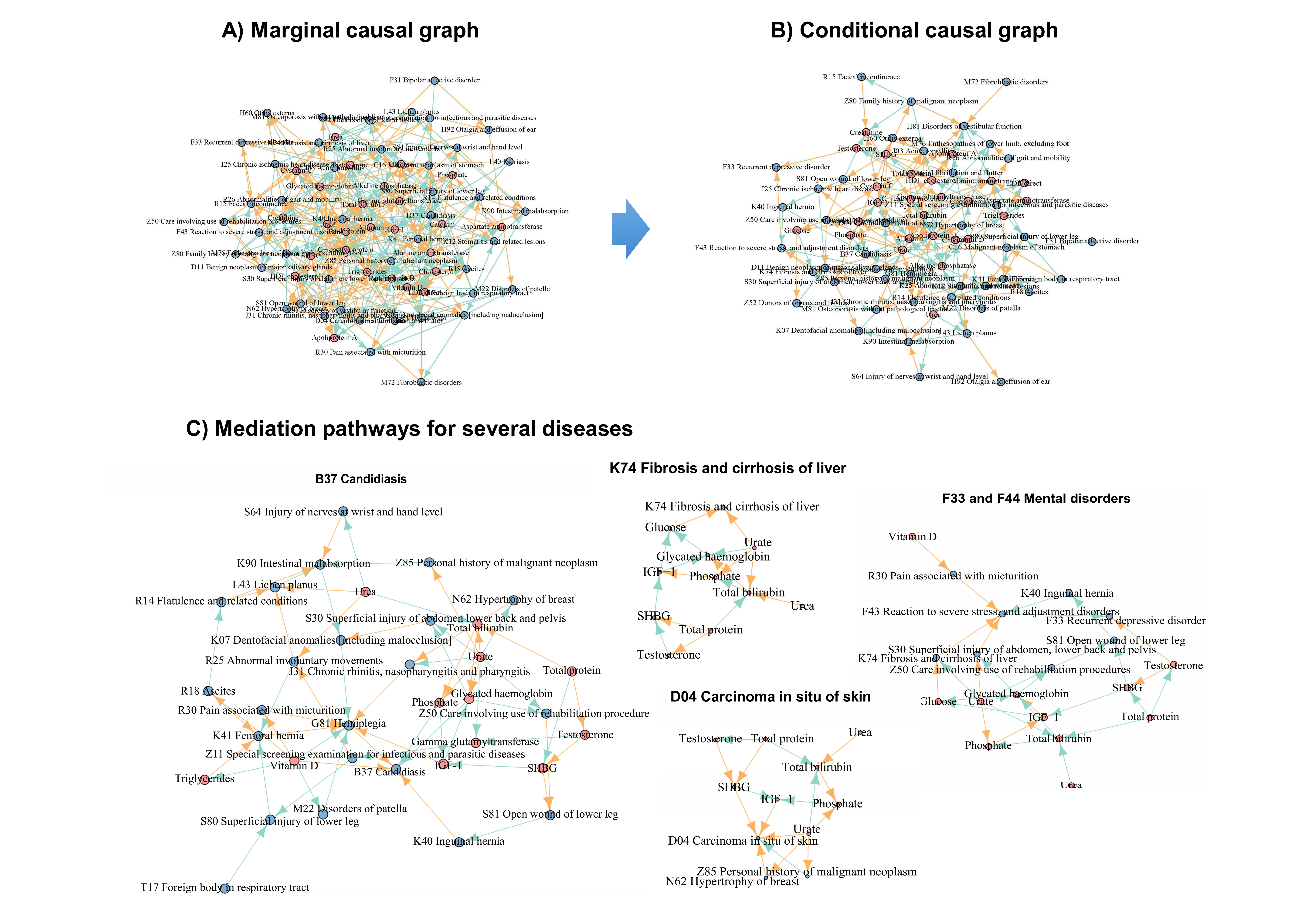

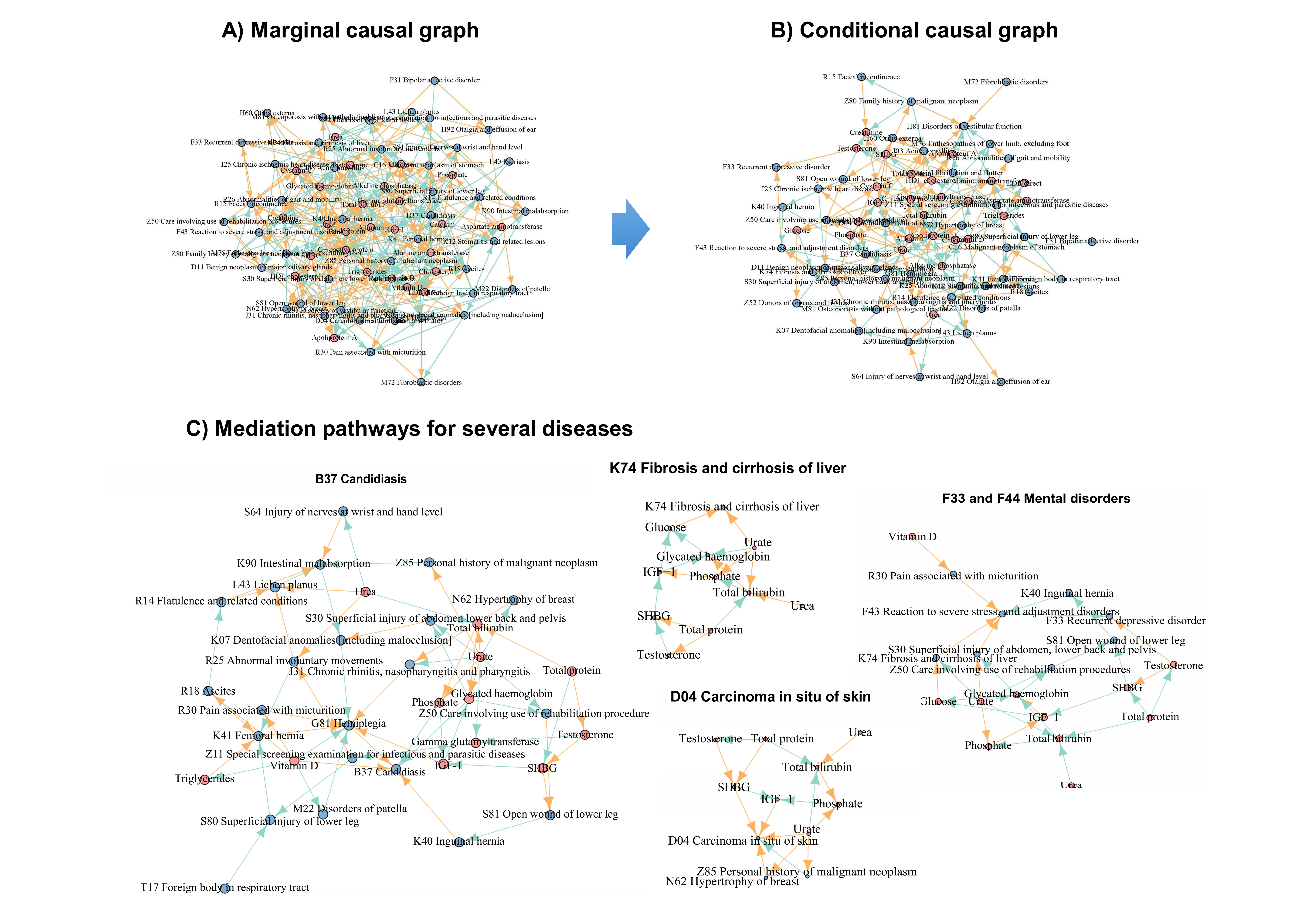


# Figure S48. Marginal and Conditional causal graph in Applied example 2

# Table S1. Computing time with network of 10 continuous nodes in simulation study 2 (seconds).

| edge effect | g | prob | MRSL^1^ | MRSL^2^ | MRSL^3^ | MRPC^1^ | MRPC^2^ | HC^1^ | HC^2^ | BIMMER | cGAUGE^1^ | cGAUGE^2^ | cGAUGE^3^ |
| --- | --- | --- | --- | --- | --- | --- | --- | --- | --- | --- | --- | --- | --- |
| 0-0.25 | 5 | 0.2 | 0.44 | 0.63 | 0.01 | 10.18 | 9.98 | 1.38 | 1.09 | 9.63 | 89.86 | 93.17 | 153.23 |
|  |  | 0.5 | 1.16 | 1.55 | 0.01 | 12.42 | 11.49 | 2.24 | 1.62 | 10.06 | 104.54 | 106.87 | 167.74 |
|  |  | 0.8 | 2.86 | 2.49 | 0.01 | 11.00 | 11.73 | 2.70 | 2.27 | 9.62 | 107.74 | 105.53 | 162.45 |
|  | 10 | 0.2 | 0.49 | 0.70 | 0.01 | 62.28 | 11.50 | 2.28 | 1.22 | 8.00 | 170.85 | 174.88 | 369.29 |
|  |  | 0.5 | 1.40 | 1.51 | 0.01 | 59.68 | 11.10 | 3.03 | 1.58 | 9.05 | 176.03 | 182.82 | 372.44 |
|  |  | 0.8 | 4.18 | 3.30 | 0.02 | 66.91 | 10.58 | 3.17 | 2.08 | 9.35 | 149.90 | 141.86 | 274.87 |
|  | 20 | 0.2 | 0.25 | 0.33 | 0.01 | 318.35 | 6.99 | 2.00 | 0.86 | 3.47 | 187.00 | 180.95 | 374.32 |
|  |  | 0.5 | 0.79 | 0.89 | 0.02 | 524.53 | 5.94 | 2.47 | 1.08 | 4.07 | 188.95 | 185.82 | 375.04 |
|  |  | 0.8 | 1.97 | 1.95 | 0.03 | 225.19 | 5.92 | 2.44 | 1.54 | 5.02 | 189.15 | 186.96 | 377.61 |
|  | 30 | 0.2 | 0.26 | 0.33 | 0.02 | 358.74 | 6.42 | 1.74 | 0.77 | 3.38 | 264.44 | 261.51 | 483.54 |
|  |  | 0.5 | 0.81 | 0.80 | 0.03 | 433.10 | 6.45 | 2.46 | 1.08 | 4.28 | 291.43 | 304.86 | 632.83 |
|  |  | 0.8 | 3.20 | 3.06 | 0.09 | 532.94 | 11.56 | 3.49 | 2.27 | 9.92 | 472.89 | 468.45 | 836.35 |
|  | 40 | 0.2 | 0.41 | 0.54 | 0.06 | 438.62 | 13.90 | 2.84 | 1.46 | 6.45 | 609.54 | 625.28 | 1107.09 |
|  |  | 0.5 | 1.65 | 1.62 | 0.10 | 1159.72 | 11.64 | 3.63 | 1.83 | 7.18 | 602.11 | 565.83 | 1049.76 |
|  |  | 0.8 | 3.53 | 4.00 | 0.16 | 548.12 | 11.62 | 3.61 | 2.52 | 11.34 | 630.67 | 618.76 | 1037.17 |
|  | 50 | 0.2 | 0.43 | 0.54 | 0.08 | 454.50 | 13.33 | 2.69 | 1.45 | 6.33 | 769.21 | 755.44 | 1177.71 |
|  |  | 0.5 | 1.62 | 1.62 | 0.15 | 620.90 | 11.57 | 3.31 | 1.86 | 7.64 | 761.88 | 716.36 | 1161.77 |
|  |  | 0.8 | 3.58 | 3.66 | 0.24 | 556.61 | 11.41 | 3.33 | 2.51 | 11.42 | 740.36 | 716.61 | 1130.72 |
| 0.25-0.5 | 5 | 0.2 | 1.17 | 1.74 | 0.01 | 7.78 | 11.09 | 2.06 | 1.33 | 12.31 | 112.22 | 108.91 | 141.70 |
|  |  | 0.5 | 6.29 | 4.16 | 0.02 | 5.34 | 10.03 | 2.65 | 2.99 | 8.63 | 115.11 | 112.67 | 139.95 |
|  |  | 0.8 | 8.35 | 6.30 | 0.03 | 3.90 | 8.87 | 2.25 | 3.50 | 6.98 | 122.92 | 111.16 | 122.10 |
|  | 10 | 0.2 | 1.02 | 1.31 | 0.01 | 23.29 | 8.86 | 2.48 | 1.19 | 10.84 | 189.62 | 184.54 | 369.08 |
|  |  | 0.5 | 5.43 | 3.77 | 0.03 | 12.91 | 8.99 | 2.59 | 2.78 | 9.34 | 194.07 | 197.01 | 345.75 |
|  |  | 0.8 | 6.93 | 6.53 | 0.04 | 3.99 | 7.54 | 1.90 | 3.09 | 6.72 | 186.45 | 191.20 | 300.31 |
|  | 20 | 0.2 | 1.10 | 1.39 | 0.03 | 110.26 | 9.65 | 2.77 | 1.21 | 8.42 | 338.80 | 332.62 | 610.84 |
|  |  | 0.5 | 6.15 | 5.02 | 0.07 | 33.04 | 8.77 | 2.73 | 3.06 | 11.34 | 362.36 | 353.72 | 571.79 |
|  |  | 0.8 | 8.54 | 10.07 | 0.08 | 5.60 | 7.53 | 2.00 | 3.34 | 7.43 | 344.11 | 339.55 | 523.73 |
|  | 30 | 0.2 | 1.33 | 1.48 | 0.06 | 98.30 | 7.28 | 2.14 | 1.05 | 8.19 | 471.69 | 477.34 | 679.46 |
|  |  | 0.5 | 6.00 | 5.28 | 0.12 | 32.94 | 8.25 | 2.48 | 2.55 | 11.39 | 508.80 | 497.39 | 639.58 |
|  |  | 0.8 | 10.46 | 13.60 | 0.10 | 4.93 | 7.28 | 1.80 | 2.97 | 7.55 | 510.18 | 483.41 | 621.97 |
|  | 40 | 0.2 | 1.24 | 1.47 | 0.10 | 65.86 | 8.39 | 2.12 | 1.23 | 8.66 | 620.99 | 628.76 | 830.62 |
|  |  | 0.5 | 6.51 | 6.63 | 0.20 | 31.50 | 7.80 | 2.26 | 2.69 | 12.14 | 648.79 | 631.50 | 786.62 |
|  |  | 0.8 | 11.71 | 16.66 | 0.11 | 4.86 | 7.49 | 1.85 | 3.15 | 7.60 | 637.01 | 635.31 | 747.73 |
|  | 50 | 0.2 | 1.31 | 1.47 | 0.14 | 56.75 | 8.11 | 1.90 | 1.17 | 8.64 | 743.79 | 757.10 | 960.77 |
|  |  | 0.5 | 7.11 | 7.50 | 0.28 | 25.80 | 8.48 | 2.41 | 2.76 | 14.58 | 791.12 | 733.85 | 942.95 |
|  |  | 0.8 | 15.77 | 18.18 | 0.11 | 4.00 | 7.47 | 1.77 | 3.15 | 7.90 | 773.35 | 755.31 | 895.70 |
| 0.5-0.75 | 5 | 0.2 | 1.28 | 1.81 | 0.01 | 4.77 | 10.17 | 2.06 | 1.53 | 10.40 | 110.09 | 105.68 | 132.13 |
|  |  | 0.5 | 6.99 | 4.42 | 0.02 | 2.86 | 5.44 | 2.43 | 3.53 | 6.77 | 116.25 | 108.11 | 115.02 |
|  |  | 0.8 | 9.75 | 8.56 | 0.03 | 1.91 | 3.25 | 1.85 | 3.15 | 5.42 | 114.58 | 108.85 | 117.62 |
|  | 10 | 0.2 | 1.43 | 1.95 | 0.02 | 12.40 | 8.78 | 2.38 | 1.40 | 11.30 | 186.49 | 183.82 | 332.79 |
|  |  | 0.5 | 6.44 | 4.89 | 0.03 | 2.59 | 4.92 | 2.08 | 3.09 | 6.58 | 180.79 | 167.62 | 251.79 |
|  |  | 0.8 | 11.43 | 13.31 | 0.03 | 1.92 | 3.15 | 1.72 | 2.86 | 5.89 | 197.75 | 191.00 | 238.67 |
|  | 20 | 0.2 | 1.52 | 1.83 | 0.04 | 24.12 | 7.52 | 2.41 | 1.50 | 9.56 | 344.63 | 323.82 | 551.96 |
|  |  | 0.5 | 8.27 | 8.21 | 0.06 | 4.20 | 4.94 | 2.04 | 3.21 | 7.29 | 351.35 | 350.21 | 442.85 |
|  |  | 0.8 | 12.58 | 17.88 | 0.03 | 1.52 | 2.70 | 1.51 | 2.73 | 5.55 | 354.66 | 347.40 | 377.69 |
|  | 30 | 0.2 | 1.73 | 1.95 | 0.07 | 39.08 | 7.15 | 2.08 | 1.36 | 9.95 | 495.09 | 482.30 | 648.55 |
|  |  | 0.5 | 4.95 | 5.99 | 0.05 | 3.29 | 2.31 | 1.15 | 1.80 | 3.76 | 271.43 | 269.86 | 323.41 |
|  |  | 0.8 | 6.93 | 10.06 | 0.02 | 0.75 | 1.36 | 0.84 | 1.58 | 2.90 | 269.35 | 267.84 | 281.50 |
|  | 40 | 0.2 | 0.98 | 1.12 | 0.05 | 18.69 | 4.16 | 1.23 | 0.86 | 5.12 | 341.58 | 340.39 | 443.68 |
|  |  | 0.5 | 5.97 | 7.34 | 0.06 | 1.47 | 2.37 | 1.14 | 1.86 | 3.95 | 346.86 | 345.11 | 399.00 |
|  |  | 0.8 | 7.58 | 10.74 | 0.02 | 0.68 | 1.27 | 0.82 | 1.61 | 2.93 | 344.79 | 343.40 | 358.77 |
|  | 50 | 0.2 | 1.20 | 1.23 | 0.08 | 15.27 | 3.37 | 1.05 | 0.78 | 5.15 | 417.83 | 416.32 | 517.33 |
|  |  | 0.5 | 8.42 | 12.04 | 0.10 | 2.00 | 3.54 | 1.53 | 2.63 | 5.72 | 589.59 | 651.33 | 864.82 |
|  |  | 0.8 | 15.49 | 23.46 | 0.03 | 1.43 | 3.10 | 1.41 | 2.77 | 5.86 | 779.40 | 762.43 | 789.32 |
| 0.75-1 | 5 | 0.2 | 1.38 | 1.68 | 0.01 | 2.50 | 5.55 | 1.86 | 1.55 | 8.89 | 105.81 | 99.97 | 113.52 |
|  |  | 0.5 | 8.34 | 6.12 | 0.02 | 1.71 | 3.30 | 2.30 | 3.33 | 6.15 | 119.01 | 112.45 | 113.43 |
|  |  | 0.8 | 10.01 | 10.06 | 0.02 | 1.22 | 1.30 | 1.41 | 2.20 | 4.24 | 101.53 | 92.05 | 103.46 |
|  | 10 | 0.2 | 1.98 | 2.10 | 0.02 | 9.48 | 6.78 | 2.39 | 1.78 | 10.15 | 187.27 | 182.40 | 285.24 |
|  |  | 0.5 | 10.06 | 9.33 | 0.03 | 1.62 | 3.12 | 1.90 | 3.08 | 6.18 | 188.20 | 180.01 | 228.00 |
|  |  | 0.8 | 13.86 | 17.23 | 0.02 | 1.34 | 1.65 | 1.53 | 2.65 | 5.67 | 194.16 | 189.48 | 214.66 |
|  | 20 | 0.2 | 1.86 | 2.07 | 0.04 | 11.28 | 4.41 | 2.05 | 1.49 | 8.57 | 334.46 | 335.41 | 429.89 |
|  |  | 0.5 | 11.23 | 13.83 | 0.05 | 1.61 | 2.68 | 1.87 | 2.98 | 6.15 | 359.54 | 347.45 | 374.20 |
|  |  | 0.8 | 14.08 | 20.04 | 0.02 | 1.24 | 1.38 | 1.26 | 2.25 | 4.97 | 345.71 | 338.63 | 342.01 |
|  | 30 | 0.2 | 2.70 | 2.70 | 0.08 | 14.25 | 6.77 | 2.12 | 1.76 | 9.16 | 497.69 | 455.60 | 628.40 |
|  |  | 0.5 | 11.16 | 14.84 | 0.06 | 1.52 | 2.82 | 1.74 | 3.04 | 6.61 | 492.69 | 494.02 | 542.91 |
|  |  | 0.8 | 15.16 | 22.26 | 0.02 | 1.11 | 1.50 | 1.35 | 2.47 | 5.07 | 491.96 | 486.01 | 477.01 |
|  | 40 | 0.2 | 2.93 | 3.06 | 0.11 | 12.11 | 6.45 | 1.95 | 1.66 | 8.79 | 628.35 | 613.06 | 774.53 |
|  |  | 0.5 | 12.29 | 16.44 | 0.07 | 1.34 | 2.67 | 1.69 | 3.06 | 5.85 | 633.40 | 639.30 | 693.39 |
|  |  | 0.8 | 14.00 | 20.57 | 0.02 | 1.01 | 1.36 | 1.22 | 2.28 | 4.68 | 613.71 | 637.82 | 641.41 |
|  | 50 | 0.2 | 3.44 | 3.46 | 0.16 | 40.28 | 7.01 | 1.82 | 1.64 | 9.37 | 758.43 | 758.60 | 928.54 |
|  |  | 0.5 | 15.67 | 19.07 | 0.09 | 1.38 | 3.44 | 1.74 | 3.26 | 6.63 | 780.50 | 776.38 | 821.59 |
|  |  | 0.8 | 13.79 | 20.74 | 0.02 | 0.99 | 1.55 | 1.13 | 2.19 | 4.56 | 629.16 | 589.81 | 590.85 |

MRSL^1^, MRSL when adjusting for all nodes on the open paths; MRSL^2^, MRSL when adjusting for minimum separated set; MRSL^3^, MRSL when adjusting for V\{$X_{p}$,$X_{q}$,$S^{d}$ and U}. MRPC^1^, MRPC algorithm based on the most significant SNP; MRPC^2^, MRPC algorithm based on genetic risk score. HC^1^, HC algorithm incorporating genetic anchors based on the most significant SNP; HC^2^, HC algorithm incorporating genetic anchors based on genetic risk score. cGAUGE^1^, cGAUGE based on IVW; cGAUGE^2^, cGAUGE based on MR Egger; cGAUGE^3^, cGAUGE based on MR PRESSO.

# Table S2. Computing time with network of 10 binary nodes in simulation study 2 (seconds).

| edge effect | g | prob | MRSL^1^ | MRSL^2^ | MRSL^3^ | MRPC^1^ | MRPC^2^ | HC^1^ | HC^2^ | cGAUGE^1^ | cGAUGE^2^ | cGAUGE^3^ |
| --- | --- | --- | --- | --- | --- | --- | --- | --- | --- | --- | --- | --- |
| OR 1-1.5 | 10 | 0.2 | 0.11 | 0.13 | 0.00 | 11.75 | 1.75 | 1.38 | 0.67 | 92.53 | 91.45 | 195.02 |
|  |  | 0.5 | 0.13 | 0.16 | 0.00 | 21.69 | 4.26 | 2.15 | 1.24 | 99.18 | 98.52 | 198.48 |
|  |  | 0.8 | 0.16 | 0.21 | 0.00 | 26.13 | 5.71 | 2.27 | 1.50 | 100.97 | 100.50 | 195.60 |
|  | 30 | 0.2 | 0.13 | 0.16 | 0.01 | 195.71 | 6.67 | 2.14 | 1.26 | 251.33 | 255.37 | 591.87 |
|  |  | 0.5 | 0.32 | 0.42 | 0.03 | 164.27 | 11.71 | 3.68 | 2.40 | 452.22 | 451.41 | 1010.35 |
|  |  | 0.8 | 0.45 | 0.58 | 0.03 | 215.97 | 13.17 | 4.21 | 2.94 | 464.24 | 449.07 | 992.08 |
|  | 50 | 0.2 | 0.26 | 0.35 | 0.06 | 566.49 | 14.80 | 4.11 | 2.64 | 726.53 | 720.08 | 1559.85 |
|  |  | 0.5 | 0.39 | 0.53 | 0.07 | 247.24 | 14.79 | 3.94 | 3.12 | 754.02 | 739.02 | 1581.99 |
|  |  | 0.8 | 0.61 | 0.75 | 0.08 | 237.86 | 14.84 | 4.24 | 3.49 | 748.31 | 739.65 | 1580.56 |
|  | 70 | 0.2 | 0.28 | 0.37 | 0.10 | 257.14 | 15.67 | 4.22 | 3.37 | 1014.26 | 1017.65 | 2147.42 |
|  |  | 0.5 | 0.53 | 0.68 | 0.14 | 169.79 | 16.60 | 4.12 | 3.79 | 1014.85 | 1024.53 | 2167.73 |
|  |  | 0.8 | 0.97 | 1.05 | 0.17 | 214.79 | 17.46 | 4.25 | 4.12 | 1025.37 | 1019.90 | 2118.80 |
|  | 90 | 0.2 | 0.31 | 0.42 | 0.16 | 203.81 | 15.19 | 4.22 | 3.67 | 1302.12 | 1302.18 | 2728.89 |
|  |  | 0.5 | 0.69 | 0.80 | 0.25 | 133.96 | 18.72 | 3.95 | 4.22 | 1284.73 | 1300.76 | 2725.47 |
|  |  | 0.8 | 1.30 | 1.30 | 0.32 | 160.22 | 20.46 | 3.93 | 4.31 | 1314.60 | 1324.01 | 2693.18 |
|  | 110 | 0.2 | 0.45 | 0.57 | 0.29 | 120.10 | 17.84 | 4.21 | 4.42 | 1603.72 | 1601.96 | 3201.64 |
|  |  | 0.5 | 0.85 | 0.97 | 0.41 | 110.98 | 20.52 | 3.99 | 4.72 | 1606.83 | 1609.27 | 3170.30 |
|  |  | 0.8 | 1.46 | 1.46 | 0.47 | 107.43 | 21.83 | 3.78 | 4.80 | 1577.56 | 1578.63 | 3136.53 |
|  | 130 | 0.2 | 0.28 | 0.36 | 0.25 | 65.00 | 9.49 | 2.70 | 3.00 | 1002.92 | 993.41 | 2021.41 |
|  |  | 0.5 | 0.61 | 0.63 | 0.36 | 59.38 | 11.36 | 2.50 | 3.15 | 1006.87 | 993.74 | 1953.26 |
|  |  | 0.8 | 1.04 | 1.02 | 0.43 | 47.23 | 13.11 | 2.40 | 3.30 | 1001.13 | 997.17 | 1896.63 |
|  | 150 | 0.2 | 0.39 | 0.43 | 0.41 | 52.86 | 10.88 | 2.61 | 3.20 | 1136.99 | 1135.89 | 2205.91 |
|  |  | 0.5 | 0.85 | 0.82 | 0.55 | 41.99 | 13.00 | 2.46 | 3.30 | 1135.58 | 1134.79 | 2157.91 |
|  |  | 0.8 | 1.40 | 1.41 | 0.68 | 47.00 | 13.19 | 2.38 | 3.44 | 1145.18 | 1142.79 | 2145.34 |
|  | 170 | 0.2 | 0.46 | 0.51 | 0.55 | 51.45 | 11.04 | 2.63 | 3.42 | 1329.08 | 1310.50 | 2444.80 |
|  |  | 0.5 | 1.08 | 1.10 | 0.76 | 43.82 | 13.88 | 2.53 | 3.51 | 1294.91 | 1296.44 | 2440.78 |
|  |  | 0.8 | 1.66 | 1.78 | 0.93 | 42.37 | 15.16 | 2.44 | 3.58 | 1286.82 | 1284.81 | 2341.66 |
|  | 190 | 0.2 | 0.69 | 0.67 | 0.80 | 37.59 | 11.27 | 2.50 | 3.50 | 1441.47 | 1437.31 | 2639.68 |
|  |  | 0.5 | 1.48 | 1.39 | 1.11 | 38.40 | 14.66 | 2.42 | 3.56 | 1440.29 | 1438.36 | 2598.63 |
|  |  | 0.8 | 2.03 | 2.37 | 1.26 | 32.10 | 15.84 | 2.37 | 3.67 | 1441.53 | 1438.41 | 2564.43 |
| OR 1.5-2 | 10 | 0.2 | 0.28 | 0.37 | 0.01 | 28.37 | 3.51 | 2.81 | 1.31 | 179.04 | 173.00 | 359.48 |
|  |  | 0.5 | 0.48 | 0.67 | 0.01 | 36.33 | 9.28 | 3.67 | 2.41 | 192.86 | 185.66 | 352.42 |
|  |  | 0.8 | 0.84 | 1.12 | 0.01 | 30.08 | 13.79 | 3.73 | 2.77 | 195.74 | 192.83 | 309.22 |
|  | 30 | 0.2 | 0.40 | 0.53 | 0.04 | 375.42 | 10.27 | 3.33 | 1.95 | 449.86 | 440.29 | 971.85 |
|  |  | 0.5 | 0.76 | 0.73 | 0.03 | 72.41 | 5.33 | 2.43 | 1.71 | 249.62 | 247.73 | 507.25 |
|  |  | 0.8 | 1.41 | 1.44 | 0.04 | 82.26 | 6.46 | 2.68 | 2.00 | 251.26 | 249.20 | 484.94 |
|  | 50 | 0.2 | 0.26 | 0.35 | 0.04 | 234.44 | 8.13 | 2.46 | 1.73 | 416.40 | 461.57 | 1180.79 |
|  |  | 0.5 | 1.67 | 1.59 | 0.13 | 175.00 | 15.80 | 3.94 | 3.34 | 759.47 | 723.71 | 1518.48 |
|  |  | 0.8 | 3.26 | 3.85 | 0.19 | 116.43 | 16.47 | 4.16 | 3.87 | 760.80 | 755.92 | 1454.25 |
|  | 70 | 0.2 | 0.61 | 0.73 | 0.15 | 322.64 | 19.17 | 3.99 | 3.27 | 972.43 | 1023.67 | 2180.56 |
|  |  | 0.5 | 2.07 | 2.11 | 0.26 | 116.92 | 21.75 | 3.65 | 3.62 | 991.65 | 1029.85 | 2056.59 |
|  |  | 0.8 | 3.56 | 4.59 | 0.30 | 112.96 | 21.39 | 3.91 | 4.21 | 1039.29 | 1032.17 | 1956.21 |
|  | 90 | 0.2 | 0.64 | 0.79 | 0.26 | 170.68 | 24.80 | 4.05 | 3.84 | 1289.43 | 1293.46 | 2711.82 |
|  |  | 0.5 | 2.24 | 2.24 | 0.38 | 116.32 | 30.10 | 3.66 | 4.15 | 1312.18 | 1314.58 | 2541.78 |
|  |  | 0.8 | 4.16 | 5.88 | 0.51 | 92.49 | 28.10 | 3.87 | 4.63 | 1307.34 | 1260.55 | 2441.44 |
|  | 110 | 0.2 | 0.81 | 0.92 | 0.40 | 175.06 | 30.74 | 4.08 | 4.40 | 1545.03 | 1536.41 | 3142.21 |
|  |  | 0.5 | 2.65 | 2.82 | 0.62 | 104.19 | 39.69 | 3.65 | 4.64 | 1593.42 | 1588.40 | 2970.55 |
|  |  | 0.8 | 5.12 | 7.01 | 0.67 | 90.90 | 36.35 | 3.79 | 4.95 | 1597.96 | 1589.77 | 2841.65 |
| OR 2-2.5 | 10 | 0.2 | 0.41 | 0.58 | 0.01 | 23.12 | 2.99 | 2.59 | 1.36 | 178.86 | 175.86 | 373.88 |
|  |  | 0.5 | 0.82 | 1.04 | 0.01 | 33.87 | 8.47 | 3.47 | 2.34 | 192.33 | 186.39 | 344.08 |
|  |  | 0.8 | 1.84 | 1.95 | 0.02 | 20.32 | 12.58 | 3.49 | 2.74 | 198.75 | 191.58 | 299.82 |
|  | 30 | 0.2 | 0.61 | 0.77 | 0.04 | 203.49 | 9.21 | 3.34 | 1.98 | 465.05 | 459.00 | 1005.46 |
|  |  | 0.5 | 2.03 | 1.87 | 0.07 | 69.77 | 9.27 | 3.45 | 2.61 | 444.90 | 454.24 | 937.39 |
|  |  | 0.8 | 5.00 | 5.50 | 0.12 | 60.42 | 12.82 | 4.06 | 3.41 | 480.09 | 477.25 | 859.34 |
|  | 50 | 0.2 | 0.69 | 0.82 | 0.10 | 345.23 | 17.21 | 3.78 | 2.71 | 740.55 | 704.71 | 1479.36 |
|  |  | 0.5 | 3.02 | 3.09 | 0.19 | 76.19 | 15.72 | 3.61 | 3.32 | 757.61 | 757.48 | 1519.41 |
|  |  | 0.8 | 5.49 | 7.20 | 0.25 | 64.63 | 15.36 | 3.81 | 3.85 | 756.66 | 733.77 | 1277.66 |
|  | 70 | 0.2 | 0.60 | 0.68 | 0.14 | 190.89 | 17.79 | 2.97 | 2.61 | 786.02 | 777.08 | 1280.74 |
|  |  | 0.5 | 1.56 | 1.70 | 0.15 | 60.05 | 12.14 | 2.23 | 2.31 | 548.96 | 550.71 | 1050.91 |
|  |  | 0.8 | 3.51 | 4.94 | 0.19 | 28.78 | 10.14 | 2.25 | 2.66 | 548.03 | 551.92 | 965.63 |
|  | 90 | 0.2 | 0.50 | 0.55 | 0.15 | 127.35 | 16.18 | 2.58 | 2.46 | 694.19 | 697.75 | 1409.39 |
|  |  | 0.5 | 1.82 | 2.28 | 0.27 | 41.66 | 16.79 | 2.17 | 2.60 | 691.43 | 692.45 | 1307.32 |
|  |  | 0.8 | 4.16 | 6.25 | 0.29 | 34.26 | 14.28 | 2.27 | 2.90 | 706.69 | 695.48 | 1185.10 |
|  | 110 | 0.2 | 0.60 | 0.61 | 0.26 | 82.48 | 19.15 | 2.47 | 2.72 | 842.77 | 840.28 | 1664.10 |
|  |  | 0.5 | 2.31 | 2.85 | 0.43 | 39.49 | 23.53 | 2.14 | 2.82 | 840.76 | 839.84 | 1525.87 |
|  |  | 0.8 | 4.75 | 6.96 | 0.42 | 26.21 | 17.65 | 2.19 | 3.03 | 840.98 | 840.70 | 1418.54 |
| OR 2.5-3 | 10 | 0.2 | 0.28 | 0.38 | 0.01 | 13.48 | 1.34 | 1.51 | 0.77 | 92.85 | 92.63 | 189.22 |
|  |  | 0.5 | 0.71 | 0.85 | 0.01 | 8.38 | 3.54 | 1.99 | 1.37 | 100.85 | 100.52 | 170.77 |
|  |  | 0.8 | 1.45 | 1.30 | 0.01 | 7.72 | 5.54 | 1.85 | 1.50 | 104.01 | 103.67 | 149.79 |
|  | 30 | 0.2 | 0.39 | 0.48 | 0.02 | 96.83 | 4.19 | 2.00 | 1.14 | 247.08 | 245.59 | 520.49 |
|  |  | 0.5 | 1.85 | 1.75 | 0.04 | 28.92 | 4.33 | 2.16 | 1.69 | 256.30 | 254.18 | 476.39 |
|  |  | 0.8 | 3.29 | 3.40 | 0.06 | 19.28 | 5.74 | 2.23 | 1.95 | 252.41 | 252.40 | 416.91 |
|  | 50 | 0.2 | 0.44 | 0.52 | 0.06 | 158.55 | 7.76 | 2.24 | 1.60 | 403.14 | 397.83 | 819.26 |
|  |  | 0.5 | 1.73 | 1.94 | 0.10 | 28.52 | 7.63 | 2.14 | 1.98 | 415.44 | 413.71 | 782.52 |
|  |  | 0.8 | 3.98 | 5.18 | 0.14 | 19.31 | 7.36 | 2.20 | 2.34 | 412.16 | 427.62 | 706.66 |
|  | 70 | 0.2 | 0.47 | 0.54 | 0.11 | 142.33 | 12.66 | 2.46 | 2.03 | 579.32 | 576.48 | 1188.64 |
|  |  | 0.5 | 3.48 | 3.99 | 0.37 | 56.81 | 18.94 | 2.88 | 3.15 | 890.08 | 888.39 | 1644.10 |
|  |  | 0.8 | 8.04 | 11.15 | 0.36 | 59.62 | 16.64 | 3.12 | 3.65 | 883.90 | 879.40 | 1436.30 |
|  | 90 | 0.2 | 1.02 | 1.09 | 0.35 | 164.93 | 26.69 | 3.37 | 3.28 | 1120.55 | 1114.75 | 2229.67 |
|  |  | 0.5 | 4.06 | 5.19 | 0.61 | 53.85 | 29.52 | 2.95 | 3.46 | 1127.95 | 1124.85 | 2007.56 |
|  |  | 0.8 | 9.86 | 13.85 | 0.59 | 43.05 | 22.88 | 3.00 | 3.86 | 1134.40 | 1114.28 | 1818.57 |
|  | 110 | 0.2 | 1.08 | 1.14 | 0.49 | 152.37 | 33.51 | 3.39 | 3.71 | 1383.01 | 1361.52 | 2642.23 |
|  |  | 0.5 | 5.46 | 7.00 | 0.94 | 56.50 | 37.59 | 2.85 | 3.82 | 1359.51 | 1367.08 | 2404.77 |
|  |  | 0.8 | 9.46 | 12.31 | 0.61 | 30.06 | 24.36 | 2.54 | 3.55 | 1104.28 | 1021.66 | 1630.70 |

MRSL^1^, MRSL when adjusting for all nodes on the open paths; MRSL^2^, MRSL when adjusting for minimum separated set; MRSL^3^, MRSL when adjusting for V\{$X_{p}$,$X_{q}$,$S^{d}$ and U}. MRPC^1^, MRPC algorithm based on the most significant SNP; MRPC^2^, MRPC algorithm based on genetic risk score. HC^1^, HC algorithm incorporating genetic anchors based on the most significant SNP; HC^2^, HC algorithm incorporating genetic anchors based on genetic risk score. cGAUGE^1^, cGAUGE based on IVW; cGAUGE^2^, cGAUGE based on MR Egger; cGAUGE^3^, cGAUGE based on MR PRESSO.

# Table S3. Computing time with network of 5 continuous nodes in simulation study 2 (seconds).

| edge effect | g | prob | MRSL^1^ | MRSL^2^ | MRSL^3^ | MRPC^1^ | MRPC^2^ | HC^1^ | HC^2^ | BIMMER | cGAUGE^1^ | cGAUGE^2^ | cGAUGE^3^ |
| --- | --- | --- | --- | --- | --- | --- | --- | --- | --- | --- | --- | --- | --- |
| 0-0.25 | 5 | 0.2 | 0.07 | 0.05 | 0.00 | 0.37 | 0.07 | 0.12 | 0.08 | 3.31 | 7.76 | 8.62 | 20.07 |
|  |  | 0.5 | 0.14 | 0.15 | 0.00 | 0.64 | 0.12 | 0.23 | 0.15 | 5.94 | 11.67 | 11.16 | 25.69 |
|  |  | 0.8 | 0.19 | 0.26 | 0.00 | 0.72 | 0.13 | 0.31 | 0.18 | 6.14 | 12.27 | 11.96 | 26.36 |
|  | 10 | 0.2 | 0.11 | 0.10 | 0.00 | 0.83 | 0.11 | 0.23 | 0.13 | 5.42 | 21.15 | 19.60 | 64.11 |
|  |  | 0.5 | 0.16 | 0.17 | 0.00 | 0.88 | 0.12 | 0.27 | 0.15 | 5.24 | 22.67 | 22.15 | 68.37 |
|  |  | 0.8 | 0.21 | 0.27 | 0.00 | 0.85 | 0.12 | 0.32 | 0.18 | 5.36 | 21.78 | 22.25 | 66.65 |
|  | 20 | 0.2 | 0.09 | 0.09 | 0.00 | 0.81 | 0.11 | 0.27 | 0.15 | 4.63 | 41.99 | 38.01 | 121.43 |
|  |  | 0.5 | 0.20 | 0.19 | 0.00 | 1.13 | 0.16 | 0.34 | 0.17 | 4.87 | 42.20 | 40.19 | 122.75 |
|  |  | 0.8 | 0.19 | 0.25 | 0.00 | 1.12 | 0.11 | 0.38 | 0.19 | 4.50 | 41.88 | 40.19 | 123.28 |
|  | 30 | 0.2 | 0.10 | 0.09 | 0.00 | 1.01 | 0.10 | 0.23 | 0.12 | 3.92 | 56.20 | 51.83 | 129.59 |
|  |  | 0.5 | 0.15 | 0.16 | 0.00 | 1.06 | 0.11 | 0.29 | 0.15 | 5.68 | 58.93 | 58.42 | 153.99 |
|  |  | 0.8 | 0.20 | 0.26 | 0.01 | 1.03 | 0.12 | 0.35 | 0.19 | 4.52 | 60.21 | 58.93 | 152.31 |
|  | 40 | 0.2 | 0.09 | 0.21 | 0.00 | 0.77 | 0.11 | 0.26 | 0.14 | 3.96 | 70.68 | 67.77 | 181.88 |
|  |  | 0.5 | 0.13 | 0.15 | 0.00 | 0.84 | 0.12 | 0.31 | 0.17 | 4.11 | 80.07 | 78.36 | 190.54 |
|  |  | 0.8 | 0.18 | 0.23 | 0.00 | 1.04 | 0.11 | 0.37 | 0.18 | 4.49 | 78.04 | 78.17 | 184.60 |
|  | 50 | 0.2 | 0.10 | 0.09 | 0.00 | 0.81 | 0.12 | 0.25 | 0.15 | 4.20 | 95.38 | 95.89 | 204.09 |
|  |  | 0.5 | 0.14 | 0.16 | 0.00 | 0.88 | 0.12 | 0.28 | 0.16 | 4.16 | 95.30 | 95.40 | 204.14 |
|  |  | 0.8 | 0.24 | 0.28 | 0.01 | 0.84 | 0.13 | 0.33 | 0.19 | 4.39 | 96.17 | 95.37 | 200.28 |
| 0.25-0.5 | 5 | 0.2 | 0.11 | 0.11 | 0.00 | 0.55 | 0.13 | 0.19 | 0.11 | 5.20 | 10.52 | 10.22 | 17.64 |
|  |  | 0.5 | 0.21 | 0.30 | 0.00 | 0.57 | 0.16 | 0.26 | 0.19 | 4.92 | 10.83 | 10.48 | 18.18 |
|  |  | 0.8 | 0.38 | 0.57 | 0.00 | 0.61 | 0.18 | 0.33 | 0.28 | 4.66 | 11.23 | 10.92 | 17.10 |
|  | 10 | 0.2 | 0.11 | 0.13 | 0.00 | 0.60 | 0.10 | 0.21 | 0.11 | 4.78 | 19.84 | 19.99 | 59.86 |
|  |  | 0.5 | 0.25 | 0.35 | 0.00 | 0.57 | 0.15 | 0.28 | 0.17 | 5.61 | 20.67 | 20.96 | 56.28 |
|  |  | 0.8 | 0.38 | 0.54 | 0.00 | 0.55 | 0.19 | 0.31 | 0.28 | 5.38 | 20.67 | 20.47 | 54.54 |
|  | 20 | 0.2 | 0.12 | 0.13 | 0.00 | 0.71 | 0.13 | 0.22 | 0.11 | 4.46 | 38.81 | 35.17 | 98.47 |
|  |  | 0.5 | 0.22 | 0.37 | 0.00 | 0.65 | 0.12 | 0.24 | 0.15 | 4.14 | 33.82 | 36.63 | 96.05 |
|  |  | 0.8 | 0.35 | 0.58 | 0.00 | 0.77 | 0.16 | 0.30 | 0.27 | 5.38 | 39.01 | 38.78 | 96.83 |
|  | 30 | 0.2 | 0.12 | 0.13 | 0.00 | 0.69 | 0.08 | 0.16 | 0.09 | 4.46 | 58.80 | 57.24 | 117.06 |
|  |  | 0.5 | 0.22 | 0.28 | 0.00 | 0.76 | 0.15 | 0.27 | 0.17 | 4.81 | 57.99 | 57.65 | 110.07 |
|  |  | 0.8 | 0.36 | 0.51 | 0.00 | 0.64 | 0.18 | 0.29 | 0.28 | 6.11 | 57.99 | 57.46 | 105.00 |
|  | 40 | 0.2 | 0.11 | 0.12 | 0.00 | 0.56 | 0.09 | 0.16 | 0.10 | 4.24 | 76.44 | 75.64 | 142.21 |
|  |  | 0.5 | 0.23 | 0.29 | 0.01 | 0.60 | 0.13 | 0.24 | 0.17 | 4.55 | 76.56 | 75.77 | 134.84 |
|  |  | 0.8 | 0.41 | 0.67 | 0.01 | 1.15 | 0.15 | 0.28 | 0.26 | 5.30 | 77.71 | 77.59 | 131.61 |
|  | 50 | 0.2 | 0.14 | 0.15 | 0.01 | 0.75 | 0.10 | 0.16 | 0.11 | 4.53 | 94.94 | 94.60 | 160.73 |
|  |  | 0.5 | 0.26 | 0.33 | 0.01 | 0.77 | 0.14 | 0.24 | 0.18 | 4.94 | 94.77 | 95.21 | 156.77 |
|  |  | 0.8 | 0.44 | 0.57 | 0.01 | 0.83 | 0.18 | 0.29 | 0.28 | 5.84 | 96.73 | 96.00 | 153.13 |
| 0.5-0.75 | 5 | 0.2 | 0.14 | 0.16 | 0.00 | 0.50 | 0.12 | 0.20 | 0.13 | 5.14 | 10.58 | 10.40 | 18.38 |
|  |  | 0.5 | 0.31 | 0.46 | 0.00 | 0.52 | 0.19 | 0.26 | 0.22 | 4.89 | 11.07 | 10.71 | 15.55 |
|  |  | 0.8 | 0.47 | 0.69 | 0.00 | 0.49 | 0.19 | 0.25 | 0.32 | 4.53 | 11.22 | 11.25 | 14.36 |
|  | 10 | 0.2 | 0.14 | 0.16 | 0.00 | 0.56 | 0.09 | 0.17 | 0.10 | 4.23 | 17.89 | 17.81 | 39.85 |
|  |  | 0.5 | 0.28 | 0.45 | 0.00 | 0.51 | 0.13 | 0.21 | 0.17 | 4.33 | 16.18 | 17.12 | 41.77 |
|  |  | 0.8 | 0.50 | 0.76 | 0.00 | 0.60 | 0.21 | 0.28 | 0.34 | 5.90 | 22.90 | 23.12 | 43.92 |
|  | 20 | 0.2 | 0.14 | 0.17 | 0.00 | 0.69 | 0.11 | 0.21 | 0.12 | 4.91 | 40.71 | 39.65 | 95.23 |
|  |  | 0.5 | 0.31 | 0.50 | 0.00 | 0.73 | 0.17 | 0.26 | 0.21 | 5.33 | 41.59 | 41.06 | 90.25 |
|  |  | 0.8 | 0.48 | 0.70 | 0.00 | 0.53 | 0.18 | 0.26 | 0.31 | 5.19 | 41.83 | 40.48 | 80.84 |
|  | 30 | 0.2 | 0.17 | 0.21 | 0.00 | 0.73 | 0.12 | 0.22 | 0.14 | 5.13 | 61.33 | 60.14 | 118.54 |
|  |  | 0.5 | 0.34 | 0.47 | 0.01 | 0.59 | 0.17 | 0.25 | 0.22 | 5.13 | 60.80 | 59.35 | 108.93 |
|  |  | 0.8 | 0.59 | 0.80 | 0.01 | 0.54 | 0.18 | 0.25 | 0.32 | 5.17 | 60.61 | 59.59 | 99.13 |
|  | 40 | 0.2 | 0.14 | 0.16 | 0.00 | 1.89 | 0.10 | 0.17 | 0.11 | 4.19 | 76.58 | 73.12 | 143.46 |
|  |  | 0.5 | 0.40 | 0.51 | 0.01 | 1.37 | 0.18 | 0.26 | 0.23 | 5.45 | 80.72 | 78.96 | 117.79 |
|  |  | 0.8 | 0.82 | 1.13 | 0.01 | 1.47 | 0.21 | 0.27 | 0.35 | 6.17 | 79.68 | 77.39 | 126.45 |
|  | 50 | 0.2 | 0.14 | 0.17 | 0.01 | 0.64 | 0.14 | 0.18 | 0.12 | 5.03 | 97.04 | 89.79 | 147.11 |
|  |  | 0.5 | 0.40 | 0.50 | 0.01 | 0.65 | 0.20 | 0.25 | 0.22 | 5.45 | 97.90 | 97.18 | 159.58 |
|  |  | 0.8 | 0.91 | 1.12 | 0.01 | 0.59 | 0.23 | 0.25 | 0.33 | 5.86 | 100.29 | 97.54 | 153.60 |
| 0.75-1 | 5 | 0.2 | 0.12 | 0.14 | 0.00 | 0.49 | 0.11 | 0.17 | 0.11 | 5.09 | 10.14 | 9.34 | 14.21 |
|  |  | 0.5 | 0.22 | 0.31 | 0.00 | 0.36 | 0.12 | 0.18 | 0.18 | 3.42 | 9.59 | 9.10 | 11.75 |
|  |  | 0.8 | 0.53 | 0.62 | 0.00 | 0.42 | 0.15 | 0.20 | 0.26 | 3.53 | 10.01 | 9.55 | 11.12 |
|  | 10 | 0.2 | 0.14 | 0.17 | 0.00 | 0.50 | 0.12 | 0.20 | 0.12 | 4.85 | 21.09 | 20.62 | 53.80 |
|  |  | 0.5 | 0.28 | 0.38 | 0.00 | 0.53 | 0.20 | 0.24 | 0.22 | 4.82 | 20.96 | 20.94 | 41.33 |
|  |  | 0.8 | 0.59 | 0.89 | 0.00 | 0.47 | 0.19 | 0.21 | 0.30 | 4.38 | 21.45 | 21.20 | 34.69 |
|  | 20 | 0.2 | 0.20 | 0.23 | 0.01 | 0.76 | 0.11 | 0.22 | 0.13 | 6.28 | 45.56 | 42.37 | 78.88 |
|  |  | 0.5 | 0.38 | 0.56 | 0.00 | 0.63 | 0.16 | 0.23 | 0.21 | 5.40 | 41.63 | 40.99 | 70.59 |
|  |  | 0.8 | 0.96 | 1.26 | 0.01 | 0.55 | 0.20 | 0.21 | 0.29 | 4.93 | 41.54 | 39.26 | 58.86 |
|  | 30 | 0.2 | 0.14 | 0.16 | 0.00 | 0.75 | 0.10 | 0.17 | 0.12 | 5.20 | 60.08 | 60.31 | 119.79 |
|  |  | 0.5 | 0.36 | 0.50 | 0.01 | 0.63 | 0.15 | 0.19 | 0.19 | 4.63 | 57.84 | 61.16 | 94.72 |
|  |  | 0.8 | 0.90 | 1.28 | 0.01 | 0.54 | 0.17 | 0.19 | 0.27 | 4.59 | 58.79 | 58.36 | 84.75 |
|  | 40 | 0.2 | 0.18 | 0.34 | 0.01 | 1.50 | 0.12 | 0.18 | 0.13 | 5.23 | 84.65 | 81.94 | 140.78 |
|  |  | 0.5 | 0.52 | 0.72 | 0.01 | 1.31 | 0.17 | 0.23 | 0.22 | 5.27 | 81.44 | 81.45 | 127.30 |
|  |  | 0.8 | 1.10 | 1.32 | 0.01 | 0.47 | 1.27 | 0.23 | 0.29 | 4.84 | 80.08 | 80.63 | 109.58 |
|  | 50 | 0.2 | 0.13 | 0.15 | 0.00 | 0.60 | 0.10 | 0.14 | 0.10 | 4.02 | 86.30 | 91.06 | 141.97 |
|  |  | 0.5 | 0.50 | 0.66 | 0.01 | 0.58 | 0.19 | 0.21 | 0.23 | 5.08 | 90.40 | 94.33 | 146.76 |
|  |  | 0.8 | 1.07 | 1.31 | 0.01 | 0.39 | 0.17 | 0.17 | 0.26 | 4.15 | 94.83 | 94.91 | 125.10 |

MRSL^1^, MRSL when adjusting for all nodes on the open paths; MRSL^2^, MRSL when adjusting for minimum separated set; MRSL^3^, MRSL when adjusting for V\{$X_{p}$,$X_{q}$,$S^{d}$ and U}. MRPC^1^, MRPC algorithm based on the most significant SNP; MRPC^2^, MRPC algorithm based on genetic risk score. HC^1^, HC algorithm incorporating genetic anchors based on the most significant SNP; HC^2^, HC algorithm incorporating genetic anchors based on genetic risk score. cGAUGE^1^, cGAUGE based on IVW; cGAUGE^2^, cGAUGE based on MR Egger; cGAUGE^3^, cGAUGE based on MR PRESSO.

# Table S4. Computing time with network of 5 binary nodes in simulation study 2 (seconds).

| edge effect | g | prob | MRSL^1^ | MRSL^2^ | MRSL^3^ | MRPC^1^ | MRPC^2^ | HC^1^ | HC^2^ | cGAUGE^1^ | cGAUGE^2^ | cGAUGE^3^ |
| --- | --- | --- | --- | --- | --- | --- | --- | --- | --- | --- | --- | --- |
| OR 1-1.5 | 10 | 0.2 | 0.04 | 0.03 | 0.00 | 0.40 | 0.05 | 0.12 | 0.06 | 12.37 | 11.70 | 37.54 |
|  |  | 0.5 | 0.06 | 0.04 | 0.00 | 0.50 | 0.07 | 0.20 | 0.10 | 14.22 | 13.09 | 37.84 |
|  |  | 0.8 | 0.06 | 0.04 | 0.00 | 0.59 | 0.07 | 0.24 | 0.14 | 14.53 | 12.33 | 35.63 |
|  | 30 | 0.2 | 0.05 | 0.04 | 0.00 | 0.69 | 0.08 | 0.26 | 0.15 | 36.75 | 33.27 | 104.10 |
|  |  | 0.5 | 0.05 | 0.04 | 0.00 | 0.47 | 0.07 | 0.24 | 0.13 | 34.90 | 32.54 | 100.97 |
|  |  | 0.8 | 0.06 | 0.06 | 0.00 | 0.46 | 0.06 | 0.22 | 0.15 | 35.63 | 33.05 | 101.78 |
|  | 50 | 0.2 | 0.05 | 0.04 | 0.00 | 0.50 | 0.07 | 0.27 | 0.16 | 59.52 | 54.32 | 162.89 |
|  |  | 0.5 | 0.06 | 0.06 | 0.00 | 0.43 | 0.07 | 0.25 | 0.17 | 55.05 | 53.19 | 163.48 |
|  |  | 0.8 | 0.07 | 0.07 | 0.00 | 0.42 | 0.07 | 0.24 | 0.19 | 54.59 | 52.47 | 158.40 |
|  | 70 | 0.2 | 0.05 | 0.05 | 0.00 | 0.45 | 0.07 | 0.24 | 0.19 | 74.27 | 73.04 | 219.72 |
|  |  | 0.5 | 0.06 | 0.06 | 0.00 | 0.44 | 0.07 | 0.24 | 0.20 | 74.03 | 72.94 | 217.48 |
|  |  | 0.8 | 0.08 | 0.08 | 0.00 | 0.42 | 0.07 | 0.24 | 0.21 | 74.67 | 73.40 | 216.92 |
|  | 90 | 0.2 | 0.06 | 0.05 | 0.00 | 0.43 | 0.07 | 0.24 | 0.22 | 95.72 | 94.40 | 273.26 |
|  |  | 0.5 | 0.07 | 0.08 | 0.01 | 0.45 | 0.07 | 0.24 | 0.21 | 97.24 | 94.69 | 270.18 |
|  |  | 0.8 | 0.12 | 0.13 | 0.01 | 0.67 | 0.12 | 0.32 | 0.30 | 141.07 | 135.85 | 379.21 |
|  | 110 | 0.2 | 0.06 | 0.06 | 0.01 | 0.42 | 0.07 | 0.23 | 0.24 | 115.19 | 117.11 | 344.73 |
|  |  | 0.5 | 0.10 | 0.11 | 0.01 | 0.56 | 0.12 | 0.30 | 0.30 | 132.30 | 115.65 | 325.04 |
|  |  | 0.8 | 0.14 | 0.17 | 0.01 | 0.59 | 0.14 | 0.31 | 0.31 | 151.68 | 147.34 | 419.72 |
| OR 1.5-2 | 10 | 0.2 | 0.08 | 0.07 | 0.00 | 0.61 | 0.06 | 0.18 | 0.09 | 18.33 | 17.59 | 55.72 |
|  |  | 0.5 | 0.10 | 0.09 | 0.00 | 0.70 | 0.08 | 0.28 | 0.15 | 19.38 | 18.43 | 55.69 |
|  |  | 0.8 | 0.10 | 0.11 | 0.00 | 0.65 | 0.10 | 0.35 | 0.21 | 18.89 | 18.08 | 53.46 |
|  | 30 | 0.2 | 0.09 | 0.08 | 0.00 | 0.76 | 0.11 | 0.32 | 0.18 | 54.29 | 51.49 | 161.79 |
|  |  | 0.5 | 0.12 | 0.14 | 0.00 | 0.67 | 0.10 | 0.30 | 0.19 | 49.65 | 48.36 | 145.43 |
|  |  | 0.8 | 0.19 | 0.25 | 0.00 | 0.71 | 0.13 | 0.35 | 0.26 | 54.02 | 52.63 | 152.10 |
|  | 50 | 0.2 | 0.11 | 0.12 | 0.00 | 0.76 | 0.13 | 0.38 | 0.24 | 91.02 | 88.70 | 274.20 |
|  |  | 0.5 | 0.14 | 0.17 | 0.00 | 0.64 | 0.12 | 0.28 | 0.22 | 69.43 | 59.44 | 154.00 |
|  |  | 0.8 | 0.15 | 0.18 | 0.00 | 0.45 | 0.10 | 0.23 | 0.20 | 54.32 | 52.52 | 154.52 |
|  | 70 | 0.2 | 0.07 | 0.07 | 0.00 | 0.47 | 0.08 | 0.24 | 0.19 | 74.69 | 72.84 | 219.48 |
|  |  | 0.5 | 0.11 | 0.13 | 0.01 | 0.49 | 0.10 | 0.24 | 0.21 | 74.36 | 72.92 | 215.04 |
|  |  | 0.8 | 0.16 | 0.20 | 0.01 | 0.46 | 0.13 | 0.25 | 0.23 | 74.68 | 72.83 | 210.15 |
|  | 90 | 0.2 | 0.07 | 0.07 | 0.01 | 0.42 | 0.09 | 0.23 | 0.22 | 95.10 | 93.02 | 270.65 |
|  |  | 0.5 | 0.12 | 0.15 | 0.01 | 0.43 | 0.13 | 0.24 | 0.23 | 94.49 | 93.08 | 266.61 |
|  |  | 0.8 | 0.17 | 0.21 | 0.01 | 0.45 | 0.15 | 0.23 | 0.25 | 95.08 | 93.02 | 261.28 |
|  | 110 | 0.2 | 0.08 | 0.09 | 0.01 | 0.45 | 0.09 | 0.25 | 0.24 | 114.74 | 112.38 | 323.67 |
|  |  | 0.5 | 0.13 | 0.15 | 0.01 | 0.45 | 0.13 | 0.24 | 0.24 | 114.94 | 112.46 | 316.55 |
|  |  | 0.8 | 0.19 | 0.25 | 0.01 | 0.44 | 0.17 | 0.23 | 0.25 | 114.73 | 112.32 | 309.46 |
| OR 2-2.5 | 10 | 0.2 | 0.06 | 0.05 | 0.00 | 0.38 | 0.04 | 0.12 | 0.06 | 12.04 | 11.52 | 35.21 |
|  |  | 0.5 | 0.08 | 0.08 | 0.00 | 0.39 | 0.04 | 0.18 | 0.10 | 12.49 | 11.81 | 33.99 |
|  |  | 0.8 | 0.10 | 0.13 | 0.00 | 0.39 | 0.06 | 0.23 | 0.15 | 12.62 | 12.02 | 32.85 |
|  | 30 | 0.2 | 0.07 | 0.07 | 0.00 | 0.47 | 0.06 | 0.22 | 0.12 | 33.78 | 32.49 | 102.06 |
|  |  | 0.5 | 0.11 | 0.13 | 0.00 | 0.45 | 0.07 | 0.21 | 0.14 | 34.02 | 32.58 | 98.14 |
|  |  | 0.8 | 0.16 | 0.21 | 0.00 | 0.45 | 0.08 | 0.23 | 0.18 | 33.75 | 32.38 | 95.12 |
|  | 50 | 0.2 | 0.07 | 0.07 | 0.00 | 0.47 | 0.08 | 0.26 | 0.16 | 54.55 | 52.58 | 161.98 |
|  |  | 0.5 | 0.12 | 0.15 | 0.00 | 0.46 | 0.11 | 0.24 | 0.18 | 54.43 | 52.49 | 156.28 |
|  |  | 0.8 | 0.17 | 0.22 | 0.00 | 0.46 | 0.13 | 0.24 | 0.22 | 54.17 | 52.57 | 151.85 |
|  | 70 | 0.2 | 0.08 | 0.08 | 0.00 | 0.49 | 0.09 | 0.25 | 0.19 | 74.33 | 72.85 | 218.08 |
|  |  | 0.5 | 0.12 | 0.15 | 0.01 | 0.47 | 0.13 | 0.23 | 0.21 | 74.41 | 72.91 | 211.78 |
|  |  | 0.8 | 0.19 | 0.24 | 0.01 | 0.45 | 0.17 | 0.22 | 0.23 | 74.92 | 72.87 | 205.96 |
|  | 90 | 0.2 | 0.08 | 0.08 | 0.01 | 0.44 | 0.10 | 0.24 | 0.22 | 95.51 | 93.14 | 272.50 |
|  |  | 0.5 | 0.14 | 0.17 | 0.01 | 0.44 | 0.15 | 0.23 | 0.23 | 95.54 | 93.24 | 262.27 |
|  |  | 0.8 | 0.20 | 0.25 | 0.01 | 0.44 | 0.20 | 0.24 | 0.25 | 95.44 | 93.35 | 255.26 |
|  | 110 | 0.2 | 0.08 | 0.09 | 0.01 | 0.46 | 0.10 | 0.25 | 0.23 | 114.87 | 112.47 | 320.31 |
|  |  | 0.5 | 0.14 | 0.17 | 0.01 | 0.44 | 0.16 | 0.22 | 0.24 | 115.83 | 112.48 | 312.84 |
|  |  | 0.8 | 0.21 | 0.26 | 0.01 | 0.45 | 0.21 | 0.23 | 0.27 | 116.24 | 112.44 | 305.82 |
| OR 2.5-3 | 10 | 0.2 | 0.07 | 0.07 | 0.00 | 0.38 | 0.04 | 0.13 | 0.06 | 12.23 | 11.57 | 35.98 |
|  |  | 0.5 | 0.10 | 0.10 | 0.00 | 0.43 | 0.06 | 0.19 | 0.11 | 12.53 | 11.86 | 33.70 |
|  |  | 0.8 | 0.14 | 0.18 | 0.00 | 0.48 | 0.08 | 0.24 | 0.17 | 12.26 | 11.94 | 31.97 |
|  | 30 | 0.2 | 0.07 | 0.08 | 0.00 | 0.47 | 0.07 | 0.21 | 0.12 | 33.79 | 32.52 | 100.45 |
|  |  | 0.5 | 0.12 | 0.16 | 0.00 | 0.46 | 0.08 | 0.21 | 0.14 | 34.03 | 32.68 | 96.01 |
|  |  | 0.8 | 0.17 | 0.22 | 0.00 | 0.45 | 0.11 | 0.24 | 0.18 | 33.91 | 32.53 | 92.97 |
|  | 50 | 0.2 | 0.07 | 0.07 | 0.00 | 0.48 | 0.07 | 0.24 | 0.15 | 54.62 | 52.54 | 160.31 |
|  |  | 0.5 | 0.12 | 0.15 | 0.00 | 0.45 | 0.11 | 0.23 | 0.17 | 54.88 | 52.56 | 153.76 |
|  |  | 0.8 | 0.18 | 0.24 | 0.00 | 0.44 | 0.14 | 0.23 | 0.21 | 54.57 | 52.66 | 147.50 |
|  | 70 | 0.2 | 0.08 | 0.08 | 0.00 | 0.47 | 0.09 | 0.25 | 0.19 | 74.28 | 72.30 | 217.10 |
|  |  | 0.5 | 0.13 | 0.16 | 0.01 | 0.44 | 0.13 | 0.23 | 0.21 | 74.88 | 72.33 | 209.29 |
|  |  | 0.8 | 0.20 | 0.25 | 0.01 | 0.44 | 0.17 | 0.23 | 0.23 | 74.68 | 72.91 | 202.08 |
|  | 90 | 0.2 | 0.08 | 0.09 | 0.01 | 0.45 | 0.10 | 0.24 | 0.22 | 94.39 | 93.08 | 270.03 |
|  |  | 0.5 | 0.14 | 0.17 | 0.01 | 0.46 | 0.15 | 0.23 | 0.22 | 94.69 | 93.23 | 260.03 |
|  |  | 0.8 | 0.21 | 0.26 | 0.01 | 0.43 | 0.20 | 0.21 | 0.25 | 94.74 | 93.22 | 252.15 |
|  | 110 | 0.2 | 0.08 | 0.09 | 0.01 | 0.44 | 0.11 | 0.23 | 0.24 | 114.97 | 112.41 | 319.35 |
|  |  | 0.5 | 0.16 | 0.19 | 0.01 | 0.46 | 0.17 | 0.22 | 0.25 | 115.92 | 112.65 | 309.02 |
|  |  | 0.8 | 0.23 | 0.29 | 0.01 | 0.42 | 0.23 | 0.22 | 0.27 | 115.85 | 112.60 | 298.49 |

MRSL^1^, MRSL when adjusting for all nodes on the open paths; MRSL^2^, MRSL when adjusting for minimum separated set; MRSL^3^, MRSL when adjusting for V\{$X_{p}$,$X_{q}$,$S^{d}$ and U}. MRPC^1^, MRPC algorithm based on the most significant SNP; MRPC^2^, MRPC algorithm based on genetic risk score. HC^1^, HC algorithm incorporating genetic anchors based on the most significant SNP; HC^2^, HC algorithm incorporating genetic anchors based on genetic risk score. cGAUGE^1^, cGAUGE based on IVW; cGAUGE^2^, cGAUGE based on MR Egger; cGAUGE^3^, cGAUGE based on MR PRESSO.

# Table S5. Computing time with network of 15 continuous nodes in simulation study 2 (seconds).

| edge effect | g | prob | MRSL^1^ | MRSL^2^ | MRSL^3^ | HC^1^ | HC^2^ | BIMMER |
| --- | --- | --- | --- | --- | --- | --- | --- | --- |
| 0-0.25 | 5 | 0.2 | 0.17 | 0.83 | 0.91 | 19.11 | 1.22 | 0.70 |
|  |  | 0.5 | 0.10 | 0.90 | 0.96 | 50.75 | 2.33 | 1.94 |
|  |  | 0.8 | 0.12 | 0.88 | 0.95 | 77.52 | 3.11 | 5.75 |
|  | 10 | 0.2 | 0.37 | 0.63 | 0.85 | 17.27 | 2.26 | 3.63 |
|  |  | 0.35 | 0.30 | 0.70 | 0.91 | 31.57 | 2.88 | 6.43 |
|  |  | 0.8 | 0.19 | 0.81 | 0.98 | 75.55 | 3.67 | 7.38 |
|  | 20 | 0.2 | 1.00 | 0.89 | 0.05 | 7.93 | 2.53 | 5.37 |
|  |  | 0.5 | 3.10 | 2.41 | 0.08 | 8.32 | 3.68 | 10.16 |
|  |  | 0.8 | 4.55 | 5.34 | 0.10 | 6.92 | 4.66 | 19.08 |
|  | 30 | 0.2 | 0.98 | 0.87 | 0.09 | 7.50 | 3.01 | 5.30 |
|  |  | 0.5 | 3.03 | 2.57 | 0.14 | 7.90 | 4.82 | 13.01 |
|  |  | 0.8 | 4.70 | 6.38 | 0.19 | 6.37 | 6.69 | 23.32 |
|  | 40 | 0.2 | 1.05 | 0.90 | 0.14 | 7.30 | 3.17 | 5.20 |
|  |  | 0.5 | 5.40 | 2.79 | 0.25 | 7.55 | 5.48 | 14.30 |
|  |  | 0.8 | 4.99 | 7.78 | 0.31 | 5.99 | 7.15 | 24.15 |
|  | 50 | 0.2 | 1.04 | 0.90 | 0.22 | 6.48 | 3.26 | 5.90 |
|  |  | 0.5 | 6.55 | 2.98 | 0.39 | 6.89 | 6.15 | 15.58 |
|  |  | 0.8 | 5.35 | 9.27 | 0.49 | 5.84 | 7.69 | 25.32 |
| 0.25-0.5 | 5 | 0.2 | 0.21 | 0.79 | 0.81 | 17.16 | 1.02 | 4.74 |
|  |  | 0.5 | 0.41 | 0.59 | 0.74 | 39.00 | 1.94 | 13.78 |
|  |  | 0.8 | 0.68 | 0.32 | 0.72 | 58.12 | 2.82 | 24.78 |
|  | 10 | 0.2 | 0.24 | 0.76 | 0.98 | 20.35 | 1.78 | 1.57 |
|  |  | 0.5 | 0.31 | 0.69 | 0.93 | 46.00 | 2.28 | 5.55 |
|  |  | 0.8 | 0.64 | 0.36 | 0.78 | 60.58 | 2.92 | 22.31 |
|  | 20 | 0.2 | 4.97 | 1.73 | 0.07 | 6.05 | 2.05 | 9.20 |
|  |  | 0.5 | 18.64 | 20.25 | 0.10 | 4.72 | 2.73 | 6.07 |
|  |  | 0.8 | 12.03 | 28.61 | 0.04 | 3.23 | 3.46 | 3.67 |
|  | 30 | 0.2 | 3.16 | 1.89 | 0.12 | 5.04 | 2.29 | 13.91 |
|  |  | 0.5 | 7.90 | 39.14 | 0.16 | 4.03 | 3.61 | 7.97 |
|  |  | 0.8 | 13.80 | 21.15 | 0.06 | 2.99 | 4.93 | 3.98 |
|  | 40 | 0.2 | 3.77 | 2.06 | 0.21 | 4.73 | 2.71 | 14.93 |
|  |  | 0.5 | 9.33 | 23.66 | 0.23 | 4.17 | 4.09 | 9.43 |
|  |  | 0.8 | 14.93 | 23.50 | 0.07 | 3.01 | 5.59 | 4.34 |
|  | 50 | 0.2 | 5.25 | 2.66 | 0.34 | 4.27 | 2.83 | 17.84 |
|  |  | 0.5 | 11.03 | 23.07 | 0.30 | 4.07 | 4.64 | 9.92 |
|  |  | 0.8 | 16.71 | 25.29 | 0.08 | 2.93 | 5.96 | 4.44 |
| 0.5-0.75 | 5 | 0.2 | 0.39 | 0.61 | 0.57 | 11.94 | 3.19 | 8.82 |
|  |  | 0.5 | 0.71 | 0.29 | 0.55 | 28.32 | 5.55 | 24.08 |
|  |  | 0.8 | 0.84 | 0.16 | 0.50 | 39.90 | 7.21 | 42.73 |
|  | 10 | 0.2 | 0.30 | 0.70 | 0.91 | 18.03 | 3.65 | 2.62 |
|  |  | 0.5 | 0.65 | 0.35 | 0.67 | 32.77 | 6.41 | 19.54 |
|  |  | 0.8 | 0.84 | 0.16 | 0.52 | 41.05 | 6.80 | 42.16 |
|  | 20 | 0.2 | 9.86 | 2.51 | 0.07 | 4.73 | 4.17 | 8.71 |
|  |  | 0.5 | 10.82 | 28.46 | 0.06 | 3.96 | 8.58 | 3.78 |
|  |  | 0.8 | 14.87 | 26.58 | 0.02 | 2.78 | 8.19 | 3.03 |
|  | 30 | 0.2 | 10.64 | 3.37 | 0.14 | 4.39 | 4.93 | 10.49 |
|  |  | 0.5 | 13.40 | 27.54 | 0.07 | 3.83 | 10.01 | 4.08 |
|  |  | 0.8 | 16.17 | 25.81 | 0.03 | 2.74 | 11.51 | 3.07 |
|  | 40 | 0.2 | 11.13 | 4.41 | 0.23 | 4.27 | 6.00 | 13.16 |
|  |  | 0.5 | 14.77 | 22.06 | 0.10 | 3.88 | 10.79 | 4.23 |
|  |  | 0.8 | 17.57 | 27.53 | 0.03 | 2.74 | 9.66 | 3.23 |
|  | 50 | 0.2 | 12.06 | 6.82 | 0.52 | 4.74 | 6.18 | 19.12 |
|  |  | 0.5 | 20.16 | 23.10 | 0.13 | 3.82 | 9.79 | 4.35 |
|  |  | 0.8 | 26.26 | 39.84 | 0.05 | 3.32 | 11.85 | 4.32 |
| 0.75-1 | 5 | 0.2 | 0.65 | 0.35 | 0.35 | 7.24 | 17.97 | 13.46 |
|  |  | 0.5 | 0.84 | 0.16 | 0.34 | 17.27 | 4.07 | 35.40 |
|  |  | 0.8 | 0.92 | 0.08 | 0.29 | 23.74 | 3.02 | 59.57 |
|  | 10 | 0.2 | 0.47 | 0.53 | 0.72 | 14.65 | 9.73 | 6.52 |
|  |  | 0.5 | 0.75 | 0.25 | 0.50 | 24.52 | 4.98 | 27.99 |
|  |  | 0.8 | 7.61 | 14.05 | 0.19 | 15.24 | 1.93 | 27.84 |
|  | 20 | 0.2 | 398.11 | 6.95 | 0.09 | 4.85 | 7.15 | 6.64 |
|  |  | 0.5 | 14.58 | 77.58 | 0.04 | 3.98 | 3.49 | 3.25 |
|  |  | 0.8 | 15.75 | 25.04 | 0.02 | 3.00 | 1.68 | 2.77 |
|  | 30 | 0.2 | 564.77 | 5.11 | 0.14 | 3.99 | 7.39 | 6.42 |
|  |  | 0.5 | 21.81 | 96.78 | 0.08 | 4.72 | 3.10 | 4.24 |
|  |  | 0.8 | 35.77 | 56.04 | 0.04 | 3.55 | 1.78 | 3.49 |
|  | 40 | 0.2 | 905.81 | 17.45 | 0.45 | 7.20 | 7.60 | 15.01 |
|  |  | 0.5 | 32.68 | 156.18 | 0.13 | 6.58 | 2.43 | 6.57 |
|  |  | 0.8 | 37.13 | 58.64 | 0.04 | 5.08 | 1.39 | 5.33 |
|  | 50 | 0.2 | 1433.09 | 58.58 | 0.70 | 6.46 | 5.13 | 16.48 |
|  |  | 0.5 | 33.12 | 246.11 | 0.16 | 6.32 | 2.94 | 6.70 |
|  |  | 0.8 | 36.91 | 58.31 | 0.05 | 4.71 | 1.54 | 5.88 |

MRSL^1^, MRSL when adjusting for all nodes on the open paths; MRSL^2^, MRSL when adjusting for minimum separated set; MRSL^3^, MRSL when adjusting for V\{$X_{p}$,$X_{q}$,$S^{d}$ and U}. HC^1^, HC algorithm incorporating genetic anchors based on the most significant SNP; HC^2^, HC algorithm incorporating genetic anchors based on genetic risk score. MRPC and cGAUGE are not listed due to their huge time consuming.

# Table S6. Computing time with network of 15 binary nodes in simulation study 2 (seconds).

| edge effect | g | prob | MRSL^1^ | MRSL^2^ | MRSL^3^ | HC^1^ | HC^2^ |
| --- | --- | --- | --- | --- | --- | --- | --- |
| OR 1-1.5 | 10 | 0.2 | 0.63 | 0.93 | 0.04 | 11.18 | 5.88 |
|  |  | 0.5 | 0.79 | 1.11 | 0.04 | 14.91 | 9.63 |
|  |  | 0.8 | 1.01 | 1.28 | 0.04 | 14.90 | 10.34 |
|  | 30 | 0.2 | 0.86 | 1.00 | 0.20 | 14.01 | 7.96 |
|  |  | 0.5 | 1.66 | 1.62 | 0.26 | 16.01 | 10.54 |
|  |  | 0.8 | 2.62 | 2.31 | 0.33 | 16.68 | 11.52 |
|  | 50 | 0.2 | 0.90 | 1.12 | 0.49 | 15.13 | 10.40 |
|  |  | 0.5 | 2.76 | 2.11 | 0.70 | 16.60 | 12.53 |
|  |  | 0.8 | 4.73 | 3.06 | 0.83 | 15.86 | 12.90 |
|  | 70 | 0.2 | 1.55 | 1.53 | 1.19 | 15.70 | 13.02 |
|  |  | 0.5 | 3.36 | 2.78 | 1.49 | 16.61 | 14.90 |
|  |  | 0.8 | 7.69 | 5.83 | 1.95 | 16.13 | 15.44 |
|  | 90 | 0.2 | 1.80 | 1.79 | 2.08 | 15.97 | 15.19 |
|  |  | 0.5 | 5.98 | 3.44 | 2.55 | 15.70 | 16.35 |
|  |  | 0.8 | 15.24 | 7.89 | 3.24 | 16.27 | 17.46 |
|  | 110 | 0.2 | 2.36 | 2.09 | 2.95 | 15.42 | 16.47 |
|  |  | 0.5 | 6.53 | 5.32 | 4.13 | 15.54 | 18.29 |
|  |  | 0.8 | 9.52 | 11.13 | 4.77 | 15.70 | 18.99 |
| OR 1.5-2 | 10 | 0.2 | 1.11 | 1.47 | 0.05 | 11.91 | 6.69 |
|  |  | 0.5 | 2.39 | 2.25 | 0.06 | 12.63 | 8.79 |
|  |  | 0.8 | 4.11 | 3.23 | 0.06 | 12.05 | 9.53 |
|  | 30 | 0.2 | 2.68 | 2.27 | 0.31 | 13.71 | 7.99 |
|  |  | 0.5 | 7.93 | 7.09 | 0.49 | 15.28 | 10.52 |
|  |  | 0.8 | 20.12 | 14.43 | 0.76 | 13.93 | 11.12 |
|  | 50 | 0.2 | 3.45 | 3.01 | 0.86 | 14.52 | 10.06 |
|  |  | 0.5 | 10.89 | 11.15 | 1.45 | 14.20 | 11.93 |
|  |  | 0.8 | 15.16 | 28.04 | 2.06 | 14.09 | 12.58 |
|  | 70 | 0.2 | 4.32 | 3.78 | 1.76 | 14.52 | 12.34 |
|  |  | 0.5 | 22.28 | 17.82 | 3.04 | 13.70 | 13.22 |
|  |  | 0.8 | 22.84 | 48.81 | 4.30 | 12.91 | 13.08 |
|  | 90 | 0.2 | 4.18 | 4.31 | 2.98 | 13.82 | 14.30 |
|  |  | 0.5 | 12.23 | 27.04 | 5.08 | 13.55 | 15.20 |
|  |  | 0.8 | 30.78 | 56.72 | 5.73 | 13.05 | 14.81 |
|  | 110 | 0.2 | 6.05 | 5.99 | 4.47 | 14.28 | 16.05 |
|  |  | 0.5 | 233.05 | 30.45 | 8.11 | 12.56 | 15.21 |
|  |  | 0.8 | 44.24 | 76.69 | 7.86 | 13.35 | 17.02 |
| OR 2-2.5 | 10 | 0.2 | 1.98 | 2.22 | 0.05 | 11.05 | 6.36 |
|  |  | 0.5 | 4.76 | 3.59 | 0.07 | 11.19 | 8.34 |
|  |  | 0.8 | 9.72 | 5.25 | 0.08 | 9.59 | 7.75 |
|  | 30 | 0.2 | 4.40 | 3.55 | 0.36 | 12.11 | 7.38 |
|  |  | 0.5 | 20.22 | 12.27 | 0.65 | 12.57 | 9.57 |
|  |  | 0.8 | 22.76 | 27.92 | 0.96 | 11.38 | 9.53 |
|  | 50 | 0.2 | 4.91 | 4.08 | 1.02 | 13.39 | 9.79 |
|  |  | 0.5 | 12.42 | 21.41 | 1.78 | 12.17 | 10.73 |
|  |  | 0.8 | 28.83 | 48.78 | 2.49 | 11.00 | 10.42 |
|  | 70 | 0.2 | 6.84 | 5.22 | 2.24 | 12.92 | 11.65 |
|  |  | 0.5 | 50.89 | 30.63 | 3.71 | 11.74 | 12.35 |
|  |  | 0.8 | 38.05 | 79.61 | 4.12 | 11.44 | 12.59 |
|  | 90 | 0.2 | 9.08 | 6.42 | 3.37 | 13.60 | 13.70 |
|  |  | 0.5 | 21.87 | 39.90 | 6.80 | 11.90 | 14.10 |
|  |  | 0.8 | 49.61 | 75.93 | 4.97 | 11.50 | 13.99 |
|  | 110 | 0.2 | 8.25 | 9.77 | 5.75 | 13.37 | 15.13 |
|  |  | 0.5 | 27.96 | 47.61 | 9.47 | 11.68 | 15.05 |
|  |  | 0.8 | 62.09 | 207.54 | 5.55 | 10.92 | 14.53 |
| OR 2.5-3 | 10 | 0.2 | 2.60 | 2.61 | 0.06 | 10.12 | 5.97 |
|  |  | 0.5 | 7.17 | 4.72 | 0.08 | 9.71 | 7.70 |
|  |  | 0.8 | 15.07 | 6.77 | 0.10 | 8.01 | 7.18 |
|  | 30 | 0.2 | 5.23 | 4.32 | 0.40 | 12.36 | 7.56 |
|  |  | 0.5 | 18.10 | 15.85 | 0.74 | 11.48 | 9.20 |
|  |  | 0.8 | 32.43 | 38.08 | 1.08 | 9.37 | 8.40 |
|  | 50 | 0.2 | 9.25 | 5.25 | 1.03 | 12.37 | 9.20 |
|  |  | 0.5 | 120.64 | 25.00 | 2.02 | 11.30 | 10.21 |
|  |  | 0.8 | 39.76 | 155.11 | 2.42 | 9.55 | 9.60 |
|  | 70 | 0.2 | 15.97 | 6.87 | 2.27 | 12.59 | 11.12 |
|  |  | 0.5 | 107.27 | 52.24 | 4.29 | 11.32 | 11.93 |
|  |  | 0.8 | 38.05 | 79.61 | 4.12 | 9.94 | 11.50 |
|  | 90 | 0.2 | 18.68 | 8.27 | 4.07 | 12.94 | 12.81 |
|  |  | 0.5 | 27.58 | 46.18 | 6.74 | 10.67 | 12.65 |
|  |  | 0.8 | 57.47 | 206.58 | 3.39 | 9.65 | 12.03 |
|  | 110 | 0.2 | 24.67 | 10.34 | 5.71 | 12.40 | 14.49 |
|  |  | 0.5 | 35.71 | 60.29 | 8.44 | 10.65 | 14.26 |
|  |  | 0.8 | 71.87 | 121.28 | 3.70 | 9.66 | 13.42 |

MRSL^1^, MRSL when adjusting for all nodes on the open paths; MRSL^2^, MRSL when adjusting for minimum separated set; MRSL^3^, MRSL when adjusting for V\{$X_{p}$,$X_{q}$,$S^{d}$ and U}. HC^1^, HC algorithm incorporating genetic anchors based on the most significant SNP; HC^2^, HC algorithm incorporating genetic anchors based on genetic risk score. MRPC and cGAUGE are not listed due to their huge time consuming.

# Table S7. Relative spearman's footrule and Kendall’s tau for estimated topological sorting by DFS algorithm when IVs are invalid (continuous variables).

| Method | Relative spearman's footrule | | | | | Kendall’s tau | | | | |
| --- | --- | --- | --- | --- | --- | --- | --- | --- | --- | --- |
|  | 0% | 10% | 30% | 50% | 80% | 0% | 10% | 30% | 50% | 80% |
| Correlated pleiotropic IVs | | | | | | | | | | |
| MRSL_ivw | 0.034 | 0.052 | 0.072 | 0.053 | 0.057 | 0.928 | 0.890 | 0.849 | 0.890 | 0.880 |
| MRSL_egger | 0.062 | 0.085 | 0.123 | 0.085 | 0.067 | 0.870 | 0.821 | 0.731 | 0.817 | 0.854 |
| MRSL_weighted median | 0.571 | 0.571 | 0.568 | 0.567 | 0.570 | -0.955 | -0.964 | -0.945 | -0.945 | -0.952 |
| MRSL_Mode based | 0.450 | 0.481 | 0.394 | 0.368 | 0.402 | -0.206 | -0.295 | -0.047 | 0.041 | -0.047 |
| MRSL_MR.Mix | 0.110 | 0.179 | 0.171 | 0.108 | 0.138 | 0.757 | 0.588 | 0.591 | 0.757 | 0.693 |
| MRSL_MR.RAPS | 0.571 | 0.571 | 0.571 | 0.571 | 0.571 | -1.000 | -1.000 | -1.000 | -1.000 | -1.000 |
| MRSL_CDcML.DP.S | 0.495 | 0.513 | 0.486 | 0.459 | 0.450 | -0.409 | -0.505 | -0.408 | -0.324 | -0.250 |
| MRSL_CD.Egger | 0.511 | 0.431 | 0.394 | 0.351 | 0.375 | -0.359 | -0.109 | 0.002 | 0.121 | 0.079 |
| Weak IVs | | | | | | | | | | |
| MRSL_ivw | 0.046 | 0.042 | 0.066 | 0.058 | 0.062 | 0.909 | 0.911 | 0.864 | 0.878 | 0.869 |
| MRSL_egger | 0.073 | 0.070 | 0.083 | 0.073 | 0.065 | 0.850 | 0.850 | 0.823 | 0.849 | 0.861 |
| MRSL_weighted median | 0.567 | 0.570 | 0.569 | 0.571 | 0.571 | -0.867 | -0.925 | -0.947 | -0.985 | -1.000 |
| MRSL_Mode based | 0.431 | 0.464 | 0.476 | 0.507 | 0.439 | -0.145 | -0.304 | -0.386 | -0.525 | -0.259 |
| MRSL_MR.Mix | 0.147 | 0.174 | 0.250 | 0.395 | 0.512 | 0.673 | 0.591 | 0.387 | -0.107 | -0.598 |
| MRSL_MR.RAPS | 0.571 | 0.571 | 0.571 | 0.571 | 0.571 | -1.000 | -1.000 | -1.000 | -1.000 | -1.000 |
| MRSL_CDcML.DP.S | 0.467 | 0.483 | 0.508 | 0.533 | 0.571 | -0.428 | -0.439 | -0.567 | -0.670 | -0.981 |
| MRSL_CD.Egger | 0.445 | 0.460 | 0.482 | 0.531 | 0.571 | -0.146 | -0.192 | -0.320 | -0.606 | -1.000 |

# Table S8. Relative spearman's footrule and Kendall’s tau for estimated topological sorting by DFS algorithm when IVs are invalid (binary variables).

| Method | Relative spearman's footrule | | | | | Kendall’s tau | | | | |
| --- | --- | --- | --- | --- | --- | --- | --- | --- | --- | --- |
|  | 0% | 10% | 30% | 50% | 80% | 0% | 10% | 30% | 50% | 80% |
| Correlated pleiotropic IVs | | | | | | | | | | |
| MRSL_ivw | 0.061 | 0.057 | 0.036 | 0.036 | 0.045 | 0.869 | 0.881 | 0.924 | 0.924 | 0.907 |
| MRSL_egger | 0.103 | 0.096 | 0.065 | 0.066 | 0.063 | 0.774 | 0.798 | 0.856 | 0.858 | 0.869 |
| MRSL_weighted median | 0.571 | 0.571 | 0.570 | 0.570 | 0.570 | -0.999 | -1.000 | -0.996 | -0.996 | -0.996 |
| MRSL_Mode based | 0.240 | 0.235 | 0.169 | 0.111 | 0.108 | 0.422 | 0.421 | 0.604 | 0.749 | 0.763 |
| MRSL_MR.Mix | 0.042 | 0.049 | 0.039 | 0.021 | 0.049 | 0.911 | 0.888 | 0.917 | 0.956 | 0.891 |
| MRSL_MR.RAPS | 0.571 | 0.571 | 0.571 | 0.571 | 0.571 | -1.000 | -1.000 | -1.000 | -1.000 | -1.000 |
| MRSL_CDcML.DP.S | 0.551 | 0.541 | 0.536 | 0.531 | 0.504 | -0.854 | -0.830 | -0.821 | -0.784 | -0.656 |
| MRSL_CD.Egger | 0.348 | 0.249 | 0.180 | 0.160 | 0.166 | 0.139 | 0.373 | 0.571 | 0.612 | 0.613 |
| Weak IVs | | | | | | | | | | |
| MRSL_ivw | 0.035 | 0.065 | 0.031 | 0.062 | 0.079 | 0.924 | 0.864 | 0.936 | 0.868 | 0.829 |
| MRSL_egger | 0.070 | 0.090 | 0.058 | 0.079 | 0.083 | 0.846 | 0.807 | 0.879 | 0.832 | 0.822 |
| MRSL_weighted median | 0.571 | 0.571 | 0.571 | 0.571 | 0.571 | -0.999 | -1.000 | -1.000 | -1.000 | -1.000 |
| MRSL_Mode based | 0.191 | 0.204 | 0.228 | 0.249 | 0.225 | 0.544 | 0.521 | 0.458 | 0.399 | 0.449 |
| MRSL_MR.Mix | 0.031 | 0.050 | 0.064 | 0.193 | 0.479 | 0.935 | 0.893 | 0.861 | 0.521 | -0.532 |
| MRSL_MR.RAPS | 0.571 | 0.571 | 0.571 | 0.571 | 0.571 | -1.000 | -1.000 | -1.000 | -1.000 | -1.000 |
| MRSL_CDcML.DP.S | 0.548 | 0.549 | 0.564 | 0.566 | 0.571 | -0.826 | -0.866 | -0.947 | -0.973 | -1.000 |
| MRSL_CD.Egger | 0.322 | 0.449 | 0.554 | 0.571 | 0.571 | 0.203 | -0.292 | -0.887 | -0.997 | -1.000 |

# Table S9. 44 diseases and 26 biomarkers in the applied example 2.

| Description | Variable type | Source | N_non_missing | N_missing | N_controls | N_cases |
| --- | --- | --- | --- | --- | --- | --- |
| Alanine aminotransferase (quantile) | continuous_irnt | biomarkers | 344136 | 17003 | NA | NA |
| Albumin (quantile) | continuous_irnt | biomarkers | 315268 | 45871 | NA | NA |
| Alkaline phosphatase (quantile) | continuous_irnt | biomarkers | 344292 | 16847 | NA | NA |
| Apoliprotein A (quantile) | continuous_irnt | biomarkers | 313387 | 47752 | NA | NA |
| Apoliprotein B (quantile) | continuous_irnt | biomarkers | 342590 | 18549 | NA | NA |
| Aspartate aminotransferase (quantile) | continuous_irnt | biomarkers | 342990 | 18149 | NA | NA |
| C-reactive protein (quantile) | continuous_irnt | biomarkers | 343524 | 17615 | NA | NA |
| Calcium (quantile) | continuous_irnt | biomarkers | 315153 | 45986 | NA | NA |
| Cholesterol (quantile) | continuous_irnt | biomarkers | 344278 | 16861 | NA | NA |
| Creatinine (quantile) | continuous_irnt | biomarkers | 344104 | 17035 | NA | NA |
| Cystatin C (quantile) | continuous_irnt | biomarkers | 344264 | 16875 | NA | NA |
| Gamma glutamyltransferase (quantile) | continuous_irnt | biomarkers | 344104 | 17035 | NA | NA |
| Glucose (quantile) | continuous_irnt | biomarkers | 314916 | 46223 | NA | NA |
| Glycated haemoglobin (quantile) | continuous_irnt | biomarkers | 344182 | 16957 | NA | NA |
| HDL cholesterol (quantile) | continuous_irnt | biomarkers | 315133 | 46006 | NA | NA |
| IGF-1 (quantile) | continuous_irnt | biomarkers | 342439 | 18700 | NA | NA |
| LDL direct (quantile) | continuous_irnt | biomarkers | 343621 | 17518 | NA | NA |
| Phosphate (quantile) | continuous_irnt | biomarkers | 314658 | 46481 | NA | NA |
| SHBG (quantile) | continuous_irnt | biomarkers | 312215 | 48924 | NA | NA |
| Testosterone (quantile) | continuous_irnt | biomarkers | 312102 | 49037 | NA | NA |
| Total bilirubin (quantile) | continuous_irnt | biomarkers | 342829 | 18310 | NA | NA |
| Total protein (quantile) | continuous_irnt | biomarkers | 314921 | 46218 | NA | NA |
| Triglycerides (quantile) | continuous_irnt | biomarkers | 343992 | 17147 | NA | NA |
| Urate (quantile) | continuous_irnt | biomarkers | 343836 | 17303 | NA | NA |
| Urea (quantile) | continuous_irnt | biomarkers | 344052 | 17087 | NA | NA |
| Vitamin D (quantile) | continuous_irnt | biomarkers | 329247 | 31892 | NA | NA |
| B37 Candidiasis | binary | icd10 | 361194 | 0 | 360942 | 252 |
| C16 Malignant neoplasm of stomach | binary | icd10 | 361194 | 0 | 360806 | 388 |
| D04 Carcinoma in situ of skin | binary | icd10 | 361194 | 0 | 360792 | 402 |
| D11 Benign neoplasm of major salivary glands | binary | icd10 | 361194 | 0 | 360818 | 376 |
| F31 Bipolar affective disorder | binary | icd10 | 361194 | 0 | 360823 | 371 |
| F33 Recurrent depressive disorder | binary | icd10 | 361194 | 0 | 360901 | 293 |
| F43 Reaction to severe stress, and adjustment disorders | binary | icd10 | 361194 | 0 | 360967 | 227 |
| G81 Hemiplegia | binary | icd10 | 361194 | 0 | 360903 | 291 |
| H60 Otitis externa | binary | icd10 | 361194 | 0 | 360892 | 302 |
| H81 Disorders of vestibular function | binary | icd10 | 361194 | 0 | 360853 | 341 |
| H92 Otalgia and effusion of ear | binary | icd10 | 361194 | 0 | 360943 | 251 |
| I25 Chronic ischaemic heart disease | binary | icd10 | 361194 | 0 | 348425 | 12769 |
| I48 Atrial fibrillation and flutter | binary | icd10 | 361194 | 0 | 354838 | 6356 |
| J03 Acute tonsillitis | binary | icd10 | 361194 | 0 | 360789 | 405 |
| J31 Chronic rhinitis, nasopharyngitis and pharyngitis | binary | icd10 | 361194 | 0 | 360903 | 291 |
| K07 Dentofacial anomalies [including malocclusion] | binary | icd10 | 361194 | 0 | 360784 | 410 |
| K12 Stomatitis and related lesions | binary | icd10 | 361194 | 0 | 360923 | 271 |
| K40 Inguinal hernia | binary | icd10 | 361194 | 0 | 348047 | 13147 |
| K41 Femoral hernia | binary | icd10 | 361194 | 0 | 360728 | 466 |
| K74 Fibrosis and cirrhosis of liver | binary | icd10 | 361194 | 0 | 360942 | 252 |
| K90 Intestinal malabsorption | binary | icd10 | 361194 | 0 | 360272 | 922 |
| L40 Psoriasis | binary | icd10 | 361194 | 0 | 360720 | 474 |
| L43 Lichen planus | binary | icd10 | 361194 | 0 | 360820 | 374 |
| M22 Disorders of patella | binary | icd10 | 361194 | 0 | 360792 | 402 |
| M72 Fibroblastic disorders | binary | icd10 | 361194 | 0 | 358001 | 3193 |
| M76 Enthesopathies of lower limb, excluding foot | binary | icd10 | 361194 | 0 | 360905 | 289 |
| M81 Osteoporosis without pathological fracture | binary | icd10 | 361194 | 0 | 360457 | 737 |
| N62 Hypertrophy of breast | binary | icd10 | 361194 | 0 | 360645 | 549 |
| R14 Flatulence and related conditions | binary | icd10 | 361194 | 0 | 360863 | 331 |
| R15 Faecal incontinence | binary | icd10 | 361194 | 0 | 360284 | 910 |
| R18 Ascites | binary | icd10 | 361194 | 0 | 360835 | 359 |
| R25 Abnormal involuntary movements | binary | icd10 | 361194 | 0 | 360961 | 233 |
| R26 Abnormalities of gait and mobility | binary | icd10 | 361194 | 0 | 360864 | 330 |
| R30 Pain associated with micturition | binary | icd10 | 361194 | 0 | 360835 | 359 |
| S30 Superficial injury of abdomen, lower back and pelvis | binary | icd10 | 361194 | 0 | 360948 | 246 |
| S64 Injury of nerves at wrist and hand level | binary | icd10 | 361194 | 0 | 360822 | 372 |
| S80 Superficial injury of lower leg | binary | icd10 | 361194 | 0 | 360963 | 231 |
| S81 Open wound of lower leg | binary | icd10 | 361194 | 0 | 360907 | 287 |
| T17 Foreign body in respiratory tract | binary | icd10 | 361194 | 0 | 360957 | 237 |
| Z11 Special screening examination for infectious and parasitic diseases | binary | icd10 | 361194 | 0 | 360943 | 251 |
| Z50 Care involving use of rehabilitation procedures | binary | icd10 | 361194 | 0 | 360976 | 218 |
| Z52 Donors of organs and tissues | binary | icd10 | 361194 | 0 | 360917 | 277 |
| Z80 Family history of malignant neoplasm | binary | icd10 | 361194 | 0 | 360817 | 377 |
| Z85 Personal history of malignant neoplasm | binary | icd10 | 361194 | 0 | 360956 | 238 |

# Table S10. Relationships in the Figure S48

|  | From | To | beta.  Marginal | Marginal | beta.  Conditional | Conditional |
| --- | --- | --- | --- | --- | --- | --- |
| 1 | Alanine aminotransferase | Aspartate aminotransferase | 0.33 | 1 | 0.33 | 1 |
| 2 | Alanine aminotransferase | Gamma glutamyltransferase | 0.83 | 1 | - | 0 |
| 3 | Alanine aminotransferase | K40 Inguinal hernia | -0.01 | 1 | - | 0 |
| 4 | Alanine aminotransferase | R14 Flatulence and related conditions | -0.01 | 1 | - | 0 |
| 5 | Alanine aminotransferase | S81 Open wound of lower leg | -0.01 | 1 | - | 0 |
| 6 | Alanine aminotransferase | SHBG | -0.48 | 1 | - | 0 |
| 7 | Alanine aminotransferase | Total protein | 0.50 | 1 | - | 0 |
| 8 | Albumin | Apoliprotein A | -0.43 | 1 | - | 0 |
| 9 | Albumin | Calcium | 0.50 | 1 | - | 0 |
| 10 | Albumin | HDL cholesterol | -0.55 | 1 | - | 0 |
| 11 | Albumin | IGF-1 | -0.51 | 1 | - | 0 |
| 12 | Albumin | N62 Hypertrophy of breast | 0.01 | 1 | - | 0 |
| 13 | Albumin | R18 Ascites | 0.00 | 1 | - | 0 |
| 14 | Albumin | R26 Abnormalities of gait and mobility | 0.00 | 1 | - | 0 |
| 15 | Albumin | S30 Superficial injury of abdomen, lower back and pelvis | 0.00 | 1 | - | 0 |
| 16 | Albumin | S64 Injury of nerves at wrist and hand level | 0.00 | 1 | - | 0 |
| 17 | Albumin | SHBG | -0.36 | 1 | - | 0 |
| 18 | Albumin | Testosterone | -0.11 | 1 | - | 0 |
| 19 | Albumin | Total protein | 0.68 | 1 | - | 0 |
| 20 | Albumin | Triglycerides | 0.76 | 1 | - | 0 |
| 21 | Albumin | Z85 Personal history of malignant neoplasm | 0.00 | 1 | - | 0 |
| 22 | Alkaline phosphatase | B37 Candidiasis | 0.00 | 1 | - | 0 |
| 23 | Alkaline phosphatase | Calcium | 0.17 | 1 | - | 0 |
| 24 | Alkaline phosphatase | Creatinine | -0.29 | 1 | - | 0 |
| 25 | Alkaline phosphatase | Cystatin C | -0.24 | 1 | - | 0 |
| 26 | Alkaline phosphatase | Gamma glutamyltransferase | 0.35 | 1 | - | 0 |
| 27 | Alkaline phosphatase | K07 Dentofacial anomalies [including malocclusion] | 0.00 | 1 | - | 0 |
| 28 | Alkaline phosphatase | K74 Fibrosis and cirrhosis of liver | 0.00 | 1 | - | 0 |
| 29 | Alkaline phosphatase | R18 Ascites | 0.00 | 1 | - | 0 |
| 30 | Alkaline phosphatase | Total bilirubin | -0.31 | 1 | - | 0 |
| 31 | Alkaline phosphatase | Triglycerides | 0.38 | 1 | - | 0 |
| 32 | Alkaline phosphatase | Z52 Donors of organs and tissues | 0.00 | 1 | - | 0 |
| 33 | Apoliprotein A | Cholesterol | 0.64 | 1 | - | 0 |
| 34 | Apoliprotein A | HDL cholesterol | 0.93 | 1 | 0.93 | 1 |
| 35 | Apoliprotein B | Albumin | 1.16 | 1 | 1.16 | 1 |
| 36 | Apoliprotein B | Calcium | 1.42 | 1 | - | 0 |
| 37 | Apoliprotein B | Cholesterol | 1.04 | 1 | - | 0 |
| 38 | Apoliprotein B | Creatinine | -1.04 | 1 | - | 0 |
| 39 | Apoliprotein B | K07 Dentofacial anomalies [including malocclusion] | 0.01 | 1 | - | 0 |
| 40 | Apoliprotein B | K40 Inguinal hernia | -0.03 | 1 | - | 0 |
| 41 | Apoliprotein B | LDL direct | 0.94 | 1 | - | 0 |
| 42 | Apoliprotein B | R18 Ascites | 0.02 | 1 | - | 0 |
| 43 | Aspartate aminotransferase | Alanine aminotransferase | 0.68 | 1 | 0.68 | 1 |
| 44 | Aspartate aminotransferase | Cholesterol | -0.37 | 1 | - | 0 |
| 45 | Aspartate aminotransferase | Gamma glutamyltransferase | 0.62 | 1 | - | 0 |
| 46 | Aspartate aminotransferase | Glucose | 0.15 | 1 | - | 0 |
| 47 | Aspartate aminotransferase | Phosphate | 0.20 | 1 | - | 0 |
| 48 | B37 Candidiasis | Albumin | -42.75 | 1 | -42.75 | 1 |
| 49 | B37 Candidiasis | K12 Stomatitis and related lesions | -0.27 | 1 | - | 0 |
| 50 | B37 Candidiasis | M76 Enthesopathies of lower limb, excluding foot | -0.66 | 1 | - | 0 |
| 51 | B37 Candidiasis | S64 Injury of nerves at wrist and hand level | -0.68 | 1 | - | 0 |
| 52 | B37 Candidiasis | Z11 Special screening examination for infectious and parasitic diseases | 0.78 | 1 | - | 0 |
| 53 | B37 Candidiasis | Z85 Personal history of malignant neoplasm | -0.61 | 1 | - | 0 |
| 54 | C-reactive protein | Alkaline phosphatase | -0.35 | 1 | -0.35 | 1 |
| 55 | C-reactive protein | Apoliprotein A | -0.12 | 1 | -0.12 | 1 |
| 56 | C-reactive protein | Apoliprotein B | -0.16 | 1 | -0.16 | 1 |
| 57 | C-reactive protein | Cholesterol | -0.21 | 1 | - | 0 |
| 58 | C-reactive protein | Cystatin C | -0.18 | 1 | - | 0 |
| 59 | C-reactive protein | D04 Carcinoma in situ of skin | 0.00 | 1 | - | 0 |
| 60 | C-reactive protein | F43 Reaction to severe stress, and adjustment disorders | 0.00 | 1 | - | 0 |
| 61 | C-reactive protein | Gamma glutamyltransferase | 0.93 | 1 | - | 0 |
| 62 | C-reactive protein | I48 Atrial fibrillation and flutter | 0.00 | 1 | - | 0 |
| 63 | C-reactive protein | IGF-1 | -0.18 | 1 | - | 0 |
| 64 | C-reactive protein | LDL direct | -0.19 | 1 | - | 0 |
| 65 | C-reactive protein | M76 Enthesopathies of lower limb, excluding foot | 0.00 | 1 | - | 0 |
| 66 | C-reactive protein | N62 Hypertrophy of breast | 0.00 | 1 | - | 0 |
| 67 | C-reactive protein | R15 Faecal incontinence | 0.00 | 1 | - | 0 |
| 68 | C-reactive protein | R18 Ascites | 0.00 | 1 | - | 0 |
| 69 | C-reactive protein | R26 Abnormalities of gait and mobility | 0.00 | 1 | - | 0 |
| 70 | C-reactive protein | SHBG | 0.17 | 1 | - | 0 |
| 71 | C-reactive protein | Testosterone | -0.09 | 1 | - | 0 |
| 72 | C-reactive protein | Total bilirubin | 0.10 | 1 | - | 0 |
| 73 | C-reactive protein | Urate | 0.19 | 1 | - | 0 |
| 74 | C-reactive protein | Urea | -0.15 | 1 | - | 0 |
| 75 | C-reactive protein | Vitamin D | -0.08 | 1 | - | 0 |
| 76 | C16 Malignant neoplasm of stomach | Alanine aminotransferase | 30.07 | 1 | 30.07 | 1 |
| 77 | C16 Malignant neoplasm of stomach | Gamma glutamyltransferase | 48.05 | 1 | - | 0 |
| 78 | C16 Malignant neoplasm of stomach | Glucose | 33.16 | 1 | - | 0 |
| 79 | C16 Malignant neoplasm of stomach | Glycated haemoglobin | 21.40 | 1 | - | 0 |
| 80 | C16 Malignant neoplasm of stomach | K40 Inguinal hernia | -1.45 | 1 | - | 0 |
| 81 | Calcium | Albumin | 1.37 | 1 | 1.37 | 1 |
| 82 | Calcium | Alkaline phosphatase | -0.53 | 1 | -0.53 | 1 |
| 83 | Calcium | Apoliprotein B | 0.50 | 1 | 0.50 | 1 |
| 84 | Calcium | C-reactive protein | -0.37 | 1 | -0.37 | 1 |
| 85 | Calcium | Cholesterol | 0.47 | 1 | - | 0 |
| 86 | Calcium | K12 Stomatitis and related lesions | 0.00 | 1 | - | 0 |
| 87 | Calcium | LDL direct | 0.48 | 1 | - | 0 |
| 88 | Calcium | Phosphate | 0.51 | 1 | - | 0 |
| 89 | Calcium | R18 Ascites | 0.00 | 1 | - | 0 |
| 90 | Calcium | S64 Injury of nerves at wrist and hand level | 0.00 | 1 | - | 0 |
| 91 | Calcium | Total protein | 0.87 | 1 | - | 0 |
| 92 | Calcium | Triglycerides | 0.34 | 1 | - | 0 |
| 93 | Calcium | Urate | 0.25 | 1 | - | 0 |
| 94 | Cholesterol | Apoliprotein B | 1.14 | 1 | 1.14 | 1 |
| 95 | Cholesterol | Glucose | -0.63 | 1 | - | 0 |
| 96 | Cholesterol | K41 Femoral hernia | 0.03 | 1 | - | 0 |
| 97 | Cholesterol | LDL direct | 1.08 | 1 | 1.08 | 1 |
| 98 | Cholesterol | N62 Hypertrophy of breast | 0.04 | 1 | - | 0 |
| 99 | Cholesterol | R18 Ascites | 0.02 | 1 | - | 0 |
| 100 | Creatinine | C-reactive protein | 0.25 | 1 | 0.25 | 1 |
| 101 | Creatinine | Cystatin C | 0.59 | 1 | 0.59 | 1 |
| 102 | Creatinine | Total bilirubin | 0.33 | 1 | - | 0 |
| 103 | Creatinine | Urate | 0.49 | 1 | - | 0 |
| 104 | Creatinine | Urea | 0.39 | 1 | - | 0 |
| 105 | Cystatin C | Creatinine | 0.81 | 1 | 0.81 | 1 |
| 106 | Cystatin C | H60 Otitis externa | 0.00 | 1 | - | 0 |
| 107 | Cystatin C | I25 Chronic ischaemic heart disease | -0.01 | 1 | - | 0 |
| 108 | Cystatin C | R25 Abnormal involuntary movements | 0.00 | 1 | - | 0 |
| 109 | Cystatin C | Urea | 0.47 | 1 | - | 0 |
| 110 | D04 Carcinoma in situ of skin | Apoliprotein B | 65.13 | 1 | 65.13 | 1 |
| 111 | D04 Carcinoma in situ of skin | H81 Disorders of vestibular function | -0.77 | 1 | - | 0 |
| 112 | D04 Carcinoma in situ of skin | LDL direct | 54.21 | 1 | - | 0 |
| 113 | D04 Carcinoma in situ of skin | Triglycerides | 73.16 | 1 | - | 0 |
| 114 | F31 Bipolar affective disorder | C16 Malignant neoplasm of stomach | -0.36 | 1 | -0.36 | 1 |
| 115 | F31 Bipolar affective disorder | H92 Otalgia and effusion of ear | 0.30 | 1 | - | 0 |
| 116 | F31 Bipolar affective disorder | Testosterone | 18.12 | 1 | - | 0 |
| 117 | F33 Recurrent depressive disorder | J03 Acute tonsillitis | -0.49 | 1 | - | 0 |
| 118 | F33 Recurrent depressive disorder | K74 Fibrosis and cirrhosis of liver | 0.48 | 1 | - | 0 |
| 119 | F33 Recurrent depressive disorder | R15 Faecal incontinence | 0.88 | 1 | - | 0 |
| 120 | F43 Reaction to severe stress, and adjustment disorders | F33 Recurrent depressive disorder | 0.84 | 1 | 0.84 | 1 |
| 121 | F43 Reaction to severe stress, and adjustment disorders | K74 Fibrosis and cirrhosis of liver | -0.58 | 1 | - | 0 |
| 122 | F43 Reaction to severe stress, and adjustment disorders | R25 Abnormal involuntary movements | 0.45 | 1 | - | 0 |
| 123 | G81 Hemiplegia | Apoliprotein B | 52.03 | 1 | 52.03 | 1 |
| 124 | G81 Hemiplegia | B37 Candidiasis | -2.35 | 1 | -2.35 | 1 |
| 125 | G81 Hemiplegia | D11 Benign neoplasm of major salivary glands | -1.68 | 1 | -1.68 | 1 |
| 126 | G81 Hemiplegia | M72 Fibroblastic disorders | 2.59 | 1 | - | 0 |
| 127 | G81 Hemiplegia | R18 Ascites | 1.27 | 1 | - | 0 |
| 128 | Gamma glutamyltransferase | Alanine aminotransferase | 0.22 | 1 | 0.22 | 1 |
| 129 | Gamma glutamyltransferase | Alkaline phosphatase | -0.17 | 1 | -0.17 | 1 |
| 130 | Gamma glutamyltransferase | Apoliprotein B | -0.15 | 1 | -0.15 | 1 |
| 131 | Gamma glutamyltransferase | Aspartate aminotransferase | 0.14 | 1 | 0.14 | 1 |
| 132 | Gamma glutamyltransferase | C-reactive protein | 0.81 | 1 | 0.81 | 1 |
| 133 | Gamma glutamyltransferase | Cholesterol | -0.18 | 1 | -0.18 | 1 |
| 134 | Gamma glutamyltransferase | Cystatin C | -0.09 | 1 | -0.09 | 1 |
| 135 | Gamma glutamyltransferase | LDL direct | -0.17 | 1 | - | 0 |
| 136 | Gamma glutamyltransferase | M76 Enthesopathies of lower limb, excluding foot | 0.00 | 1 | 0.00 | 1 |
| 137 | Gamma glutamyltransferase | Testosterone | -0.14 | 1 | - | 0 |
| 138 | Gamma glutamyltransferase | Total bilirubin | 0.16 | 1 | - | 0 |
| 139 | Gamma glutamyltransferase | Urate | 0.18 | 1 | - | 0 |
| 140 | Gamma glutamyltransferase | Urea | -0.11 | 1 | - | 0 |
| 141 | Gamma glutamyltransferase | Z11 Special screening examination for infectious and parasitic diseases | 0.00 | 1 | 0.00 | 1 |
| 142 | Glucose | K74 Fibrosis and cirrhosis of liver | -0.02 | 1 | -0.02 | 1 |
| 143 | Glucose | Testosterone | 1.38 | 1 | - | 0 |
| 144 | Glucose | Z52 Donors of organs and tissues | 0.02 | 1 | 0.02 | 1 |
| 145 | Glycated haemoglobin | Albumin | 0.18 | 1 | 0.18 | 1 |
| 146 | Glycated haemoglobin | B37 Candidiasis | 0.00 | 1 | 0.00 | 1 |
| 147 | Glycated haemoglobin | Glucose | 0.49 | 1 | 0.49 | 1 |
| 148 | Glycated haemoglobin | H60 Otitis externa | 0.00 | 1 | 0.00 | 1 |
| 149 | Glycated haemoglobin | I25 Chronic ischaemic heart disease | 0.01 | 1 | 0.01 | 1 |
| 150 | Glycated haemoglobin | IGF-1 | 0.36 | 1 | - | 0 |
| 151 | Glycated haemoglobin | S30 Superficial injury of abdomen, lower back and pelvis | 0.00 | 1 | 0.00 | 1 |
| 152 | Glycated haemoglobin | SHBG | -0.31 | 1 | - | 0 |
| 153 | Glycated haemoglobin | Total protein | 0.20 | 1 | - | 0 |
| 154 | Glycated haemoglobin | Triglycerides | 0.21 | 1 | - | 0 |
| 155 | Glycated haemoglobin | Z50 Care involving use of rehabilitation procedures | 0.00 | 1 | 0.00 | 1 |
| 156 | H81 Disorders of vestibular function | Alanine aminotransferase | -21.35 | 1 | -21.35 | 1 |
| 157 | H81 Disorders of vestibular function | Apoliprotein A | -91.87 | 1 | -91.87 | 1 |
| 158 | H81 Disorders of vestibular function | HDL cholesterol | -112.19 | 1 | - | 0 |
| 159 | H81 Disorders of vestibular function | T17 Foreign body in respiratory tract | -0.22 | 1 | - | 0 |
| 160 | H81 Disorders of vestibular function | Total bilirubin | -35.22 | 1 | - | 0 |
| 161 | H81 Disorders of vestibular function | Triglycerides | 74.51 | 1 | - | 0 |
| 162 | H81 Disorders of vestibular function | Urate | 58.85 | 1 | - | 0 |
| 163 | H92 Otalgia and effusion of ear | J03 Acute tonsillitis | 0.63 | 1 | - | 0 |
| 164 | H92 Otalgia and effusion of ear | M22 Disorders of patella | 0.49 | 1 | - | 0 |
| 165 | HDL cholesterol | Albumin | 0.33 | 1 | 0.33 | 1 |
| 166 | HDL cholesterol | Apoliprotein A | 1.07 | 1 | 1.07 | 1 |
| 167 | HDL cholesterol | Apoliprotein B | -0.48 | 1 | -0.48 | 1 |
| 168 | HDL cholesterol | IGF-1 | 0.43 | 1 | - | 0 |
| 169 | HDL cholesterol | J03 Acute tonsillitis | -0.01 | 1 | -0.01 | 1 |
| 170 | HDL cholesterol | Urate | -0.39 | 1 | - | 0 |
| 171 | I25 Chronic ischaemic heart disease | D11 Benign neoplasm of major salivary glands | -0.16 | 1 | -0.16 | 1 |
| 172 | I25 Chronic ischaemic heart disease | Glucose | 14.89 | 1 | - | 0 |
| 173 | I25 Chronic ischaemic heart disease | Glycated haemoglobin | 12.55 | 1 | - | 0 |
| 174 | I25 Chronic ischaemic heart disease | L43 Lichen planus | -0.23 | 1 | - | 0 |
| 175 | I25 Chronic ischaemic heart disease | SHBG | -7.89 | 1 | - | 0 |
| 176 | I48 Atrial fibrillation and flutter | H81 Disorders of vestibular function | -0.29 | 1 | -0.29 | 1 |
| 177 | I48 Atrial fibrillation and flutter | IGF-1 | -28.53 | 1 | - | 0 |
| 178 | I48 Atrial fibrillation and flutter | K40 Inguinal hernia | 0.35 | 1 | - | 0 |
| 179 | I48 Atrial fibrillation and flutter | M22 Disorders of patella | -0.28 | 1 | - | 0 |
| 180 | IGF-1 | Albumin | -0.28 | 1 | -0.28 | 1 |
| 181 | IGF-1 | C-reactive protein | -0.20 | 1 | -0.20 | 1 |
| 182 | IGF-1 | Cholesterol | 0.34 | 1 | 0.34 | 1 |
| 183 | IGF-1 | D04 Carcinoma in situ of skin | 0.00 | 1 | 0.00 | 1 |
| 184 | IGF-1 | G81 Hemiplegia | 0.00 | 1 | 0.00 | 1 |
| 185 | IGF-1 | Glucose | 0.20 | 1 | 0.20 | 1 |
| 186 | IGF-1 | Glycated haemoglobin | 0.23 | 1 | 0.23 | 1 |
| 187 | IGF-1 | HDL cholesterol | 0.49 | 1 | 0.49 | 1 |
| 188 | IGF-1 | J03 Acute tonsillitis | 0.00 | 1 | 0.00 | 1 |
| 189 | IGF-1 | LDL direct | 0.25 | 1 | - | 0 |
| 190 | IGF-1 | SHBG | 0.43 | 1 | - | 0 |
| 191 | IGF-1 | Total bilirubin | -0.37 | 1 | - | 0 |
| 192 | IGF-1 | Total protein | -0.24 | 1 | - | 0 |
| 193 | IGF-1 | Triglycerides | -0.45 | 1 | - | 0 |
| 194 | J03 Acute tonsillitis | Creatinine | -31.53 | 1 | -31.53 | 1 |
| 195 | J03 Acute tonsillitis | H60 Otitis externa | 0.57 | 1 | 0.57 | 1 |
| 196 | J03 Acute tonsillitis | S80 Superficial injury of lower leg | 0.30 | 1 | - | 0 |
| 197 | J03 Acute tonsillitis | Urea | -36.85 | 1 | - | 0 |
| 198 | J03 Acute tonsillitis | Z85 Personal history of malignant neoplasm | -0.37 | 1 | - | 0 |
| 199 | J31 Chronic rhinitis, nasopharyngitis and pharyngitis | G81 Hemiplegia | 0.59 | 1 | 0.59 | 1 |
| 200 | J31 Chronic rhinitis, nasopharyngitis and pharyngitis | R15 Faecal incontinence | -1.36 | 1 | - | 0 |
| 201 | J31 Chronic rhinitis, nasopharyngitis and pharyngitis | R30 Pain associated with micturition | -0.51 | 1 | - | 0 |
| 202 | J31 Chronic rhinitis, nasopharyngitis and pharyngitis | SHBG | 52.67 | 1 | - | 0 |
| 203 | J31 Chronic rhinitis, nasopharyngitis and pharyngitis | T17 Foreign body in respiratory tract | -0.33 | 1 | - | 0 |
| 204 | J31 Chronic rhinitis, nasopharyngitis and pharyngitis | Total protein | 50.40 | 1 | - | 0 |
| 205 | J31 Chronic rhinitis, nasopharyngitis and pharyngitis | Triglycerides | 38.97 | 1 | - | 0 |
| 206 | J31 Chronic rhinitis, nasopharyngitis and pharyngitis | Z85 Personal history of malignant neoplasm | 0.35 | 1 | - | 0 |
| 207 | K07 Dentofacial anomalies [including malocclusion] | G81 Hemiplegia | -0.61 | 1 | -0.61 | 1 |
| 208 | K07 Dentofacial anomalies [including malocclusion] | M22 Disorders of patella | -1.18 | 1 | - | 0 |
| 209 | K07 Dentofacial anomalies [including malocclusion] | M72 Fibroblastic disorders | -1.74 | 1 | - | 0 |
| 210 | K07 Dentofacial anomalies [including malocclusion] | R30 Pain associated with micturition | -0.31 | 1 | - | 0 |
| 211 | K12 Stomatitis and related lesions | K90 Intestinal malabsorption | -0.74 | 1 | - | 0 |
| 212 | K40 Inguinal hernia | B37 Candidiasis | -0.20 | 1 | -0.20 | 1 |
| 213 | K40 Inguinal hernia | F43 Reaction to severe stress, and adjustment disorders | -0.15 | 1 | -0.15 | 1 |
| 214 | K40 Inguinal hernia | R25 Abnormal involuntary movements | -0.15 | 1 | - | 0 |
| 215 | K40 Inguinal hernia | S30 Superficial injury of abdomen, lower back and pelvis | 0.15 | 1 | - | 0 |
| 216 | K41 Femoral hernia | C16 Malignant neoplasm of stomach | -0.28 | 1 | -0.28 | 1 |
| 217 | K41 Femoral hernia | G81 Hemiplegia | -0.52 | 1 | -0.52 | 1 |
| 218 | K41 Femoral hernia | Glucose | 19.37 | 1 | - | 0 |
| 219 | K41 Femoral hernia | M76 Enthesopathies of lower limb, excluding foot | -0.35 | 1 | - | 0 |
| 220 | K41 Femoral hernia | R25 Abnormal involuntary movements | 0.27 | 1 | - | 0 |
| 221 | K41 Femoral hernia | S81 Open wound of lower leg | 0.60 | 1 | - | 0 |
| 222 | K74 Fibrosis and cirrhosis of liver | F43 Reaction to severe stress, and adjustment disorders | 0.52 | 1 | 0.52 | 1 |
| 223 | K74 Fibrosis and cirrhosis of liver | L43 Lichen planus | -0.67 | 1 | - | 0 |
| 224 | K74 Fibrosis and cirrhosis of liver | R15 Faecal incontinence | 1.09 | 1 | - | 0 |
| 225 | K74 Fibrosis and cirrhosis of liver | S80 Superficial injury of lower leg | -0.64 | 1 | - | 0 |
| 226 | K90 Intestinal malabsorption | K07 Dentofacial anomalies [including malocclusion] | -0.43 | 1 | -0.43 | 1 |
| 227 | L40 Psoriasis | Glucose | 17.06 | 1 | - | 0 |
| 228 | L40 Psoriasis | IGF-1 | -76.45 | 1 | - | 0 |
| 229 | L40 Psoriasis | R14 Flatulence and related conditions | -0.43 | 1 | - | 0 |
| 230 | L43 Lichen planus | Alkaline phosphatase | -41.87 | 1 | -41.87 | 1 |
| 231 | L43 Lichen planus | H92 Otalgia and effusion of ear | 0.45 | 1 | 0.45 | 1 |
| 232 | L43 Lichen planus | K90 Intestinal malabsorption | -0.78 | 1 | -0.78 | 1 |
| 233 | M22 Disorders of patella | G81 Hemiplegia | -0.55 | 1 | -0.55 | 1 |
| 234 | M72 Fibroblastic disorders | H81 Disorders of vestibular function | 0.30 | 1 | 0.30 | 1 |
| 235 | M76 Enthesopathies of lower limb, excluding foot | Glycated haemoglobin | 70.85 | 1 | - | 0 |
| 236 | M76 Enthesopathies of lower limb, excluding foot | H81 Disorders of vestibular function | -0.89 | 1 | -0.89 | 1 |
| 237 | M76 Enthesopathies of lower limb, excluding foot | R15 Faecal incontinence | 1.35 | 1 | - | 0 |
| 238 | M81 Osteoporosis without pathological fracture | S80 Superficial injury of lower leg | -0.44 | 1 | - | 0 |
| 239 | M81 Osteoporosis without pathological fracture | Total bilirubin | -21.40 | 1 | - | 0 |
| 240 | N62 Hypertrophy of breast | D04 Carcinoma in situ of skin | -0.55 | 1 | -0.55 | 1 |
| 241 | N62 Hypertrophy of breast | J31 Chronic rhinitis, nasopharyngitis and pharyngitis | 0.42 | 1 | 0.42 | 1 |
| 242 | N62 Hypertrophy of breast | M76 Enthesopathies of lower limb, excluding foot | -0.43 | 1 | -0.43 | 1 |
| 243 | Phosphate | B37 Candidiasis | -0.01 | 1 | -0.01 | 1 |
| 244 | Phosphate | IGF-1 | -0.40 | 1 | -0.40 | 1 |
| 245 | Phosphate | Total protein | 0.68 | 1 | - | 0 |
| 246 | R14 Flatulence and related conditions | Albumin | -54.44 | 1 | -54.44 | 1 |
| 247 | R14 Flatulence and related conditions | Apoliprotein B | -25.24 | 1 | -25.24 | 1 |
| 248 | R14 Flatulence and related conditions | Calcium | -25.90 | 1 | -25.90 | 1 |
| 249 | R14 Flatulence and related conditions | K12 Stomatitis and related lesions | -0.64 | 1 | -0.64 | 1 |
| 250 | R14 Flatulence and related conditions | K90 Intestinal malabsorption | 1.06 | 1 | 1.06 | 1 |
| 251 | R14 Flatulence and related conditions | L43 Lichen planus | -0.47 | 1 | -0.47 | 1 |
| 252 | R14 Flatulence and related conditions | R25 Abnormal involuntary movements | -0.28 | 1 | - | 0 |
| 253 | R15 Faecal incontinence | Creatinine | 91.59 | 1 | 91.59 | 1 |
| 254 | R15 Faecal incontinence | Urate | -33.39 | 1 | - | 0 |
| 255 | R15 Faecal incontinence | Urea | 31.12 | 1 | - | 0 |
| 256 | R18 Ascites | K41 Femoral hernia | 0.73 | 1 | 0.73 | 1 |
| 257 | R18 Ascites | R14 Flatulence and related conditions | 0.76 | 1 | 0.76 | 1 |
| 258 | R18 Ascites | Triglycerides | -39.52 | 1 | - | 0 |
| 259 | R18 Ascites | Vitamin D | 41.70 | 1 | - | 0 |
| 260 | R25 Abnormal involuntary movements | Albumin | -27.68 | 1 | -27.68 | 1 |
| 261 | R25 Abnormal involuntary movements | Calcium | -20.31 | 1 | -20.31 | 1 |
| 262 | R25 Abnormal involuntary movements | K41 Femoral hernia | 0.65 | 1 | 0.65 | 1 |
| 263 | R25 Abnormal involuntary movements | L43 Lichen planus | 0.64 | 1 | 0.64 | 1 |
| 264 | R25 Abnormal involuntary movements | M81 Osteoporosis without pathological fracture | 1.11 | 1 | 1.11 | 1 |
| 265 | R25 Abnormal involuntary movements | S80 Superficial injury of lower leg | -0.32 | 1 | - | 0 |
| 266 | R26 Abnormalities of gait and mobility | H60 Otitis externa | 0.59 | 1 | 0.59 | 1 |
| 267 | R26 Abnormalities of gait and mobility | IGF-1 | 15.77 | 1 | - | 0 |
| 268 | R26 Abnormalities of gait and mobility | M76 Enthesopathies of lower limb, excluding foot | 0.32 | 1 | 0.32 | 1 |
| 269 | R26 Abnormalities of gait and mobility | Urate | -30.98 | 1 | - | 0 |
| 270 | R30 Pain associated with micturition | Apoliprotein B | -20.46 | 1 | -20.46 | 1 |
| 271 | R30 Pain associated with micturition | F43 Reaction to severe stress, and adjustment disorders | -0.68 | 1 | -0.68 | 1 |
| 272 | R30 Pain associated with micturition | G81 Hemiplegia | -1.05 | 1 | -1.05 | 1 |
| 273 | S30 Superficial injury of abdomen, lower back and pelvis | F43 Reaction to severe stress, and adjustment disorders | 1.17 | 1 | 1.17 | 1 |
| 274 | S30 Superficial injury of abdomen, lower back and pelvis | Glucose | 25.22 | 1 | - | 0 |
| 275 | S30 Superficial injury of abdomen, lower back and pelvis | K07 Dentofacial anomalies [including malocclusion] | 1.19 | 1 | 1.19 | 1 |
| 276 | S64 Injury of nerves at wrist and hand level | K90 Intestinal malabsorption | 1.09 | 1 | 1.09 | 1 |
| 277 | S64 Injury of nerves at wrist and hand level | Z52 Donors of organs and tissues | 0.90 | 1 | - | 0 |
| 278 | S80 Superficial injury of lower leg | Alanine aminotransferase | -59.94 | 1 | -59.94 | 1 |
| 279 | S80 Superficial injury of lower leg | Apoliprotein B | -107.68 | 1 | -107.68 | 1 |
| 280 | S80 Superficial injury of lower leg | Gamma glutamyltransferase | -55.55 | 1 | - | 0 |
| 281 | S80 Superficial injury of lower leg | K12 Stomatitis and related lesions | 1.09 | 1 | 1.09 | 1 |
| 282 | S80 Superficial injury of lower leg | K41 Femoral hernia | 2.21 | 1 | 2.21 | 1 |
| 283 | S80 Superficial injury of lower leg | LDL direct | -100.56 | 1 | - | 0 |
| 284 | S80 Superficial injury of lower leg | Triglycerides | -140.45 | 1 | - | 0 |
| 285 | S80 Superficial injury of lower leg | Z85 Personal history of malignant neoplasm | -1.13 | 1 | - | 0 |
| 286 | S81 Open wound of lower leg | Apoliprotein B | 30.12 | 1 | 30.12 | 1 |
| 287 | S81 Open wound of lower leg | Creatinine | -27.33 | 1 | -27.33 | 1 |
| 288 | S81 Open wound of lower leg | K40 Inguinal hernia | -1.29 | 1 | -1.29 | 1 |
| 289 | S81 Open wound of lower leg | LDL direct | 35.59 | 1 | - | 0 |
| 290 | SHBG | Apoliprotein A | 0.42 | 1 | 0.42 | 1 |
| 291 | SHBG | D04 Carcinoma in situ of skin | 0.00 | 1 | 0.00 | 1 |
| 292 | SHBG | HDL cholesterol | 0.75 | 1 | 0.75 | 1 |
| 293 | SHBG | I48 Atrial fibrillation and flutter | 0.01 | 1 | 0.01 | 1 |
| 294 | SHBG | IGF-1 | 0.34 | 1 | 0.34 | 1 |
| 295 | SHBG | S81 Open wound of lower leg | 0.00 | 1 | 0.00 | 1 |
| 296 | SHBG | Total bilirubin | 0.31 | 1 | - | 0 |
| 297 | SHBG | Triglycerides | -0.72 | 1 | - | 0 |
| 298 | SHBG | Z80 Family history of malignant neoplasm | 0.00 | 1 | 0.00 | 1 |
| 299 | T17 Foreign body in respiratory tract | C16 Malignant neoplasm of stomach | 0.59 | 1 | 0.59 | 1 |
| 300 | T17 Foreign body in respiratory tract | K12 Stomatitis and related lesions | -0.73 | 1 | -0.73 | 1 |
| 301 | T17 Foreign body in respiratory tract | S80 Superficial injury of lower leg | -0.46 | 1 | -0.46 | 1 |
| 302 | Testosterone | F33 Recurrent depressive disorder | 0.01 | 1 | 0.01 | 1 |
| 303 | Testosterone | Gamma glutamyltransferase | 0.64 | 1 | 0.64 | 1 |
| 304 | Testosterone | SHBG | -0.97 | 1 | -0.97 | 1 |
| 305 | Testosterone | Total bilirubin | -0.52 | 1 | - | 0 |
| 306 | Testosterone | Total protein | 0.65 | 1 | - | 0 |
| 307 | Testosterone | Urate | 0.90 | 1 | - | 0 |
| 308 | Testosterone | Vitamin D | -0.49 | 1 | - | 0 |
| 309 | Total bilirubin | Alanine aminotransferase | 0.34 | 1 | 0.34 | 1 |
| 310 | Total bilirubin | Apoliprotein B | -0.34 | 1 | -0.34 | 1 |
| 311 | Total bilirubin | Cholesterol | -0.41 | 1 | -0.41 | 1 |
| 312 | Total bilirubin | Cystatin C | 0.77 | 1 | 0.77 | 1 |
| 313 | Total bilirubin | Glycated haemoglobin | -0.52 | 1 | -0.52 | 1 |
| 314 | Total bilirubin | J03 Acute tonsillitis | 0.00 | 1 | 0.00 | 1 |
| 315 | Total bilirubin | LDL direct | -0.38 | 1 | -0.38 | 1 |
| 316 | Total bilirubin | Phosphate | 0.31 | 1 | 0.31 | 1 |
| 317 | Total bilirubin | Urate | 0.37 | 1 | - | 0 |
| 318 | Total protein | Alanine aminotransferase | 0.33 | 1 | 0.33 | 1 |
| 319 | Total protein | Albumin | 0.56 | 1 | 0.56 | 1 |
| 320 | Total protein | Calcium | 0.57 | 1 | 0.57 | 1 |
| 321 | Total protein | Cystatin C | 0.68 | 1 | 0.68 | 1 |
| 322 | Total protein | H81 Disorders of vestibular function | 0.01 | 1 | 0.01 | 1 |
| 323 | Total protein | I25 Chronic ischaemic heart disease | 0.04 | 1 | 0.04 | 1 |
| 324 | Total protein | J03 Acute tonsillitis | 0.01 | 1 | 0.01 | 1 |
| 325 | Total protein | SHBG | -0.69 | 1 | -0.69 | 1 |
| 326 | Total protein | Testosterone | -0.31 | 1 | -0.31 | 1 |
| 327 | Total protein | Total bilirubin | 0.33 | 1 | 0.33 | 1 |
| 328 | Total protein | Triglycerides | 0.50 | 1 | - | 0 |
| 329 | Total protein | Urate | 0.82 | 1 | - | 0 |
| 330 | Total protein | Vitamin D | -0.27 | 1 | - | 0 |
| 331 | Triglycerides | Apoliprotein B | -0.63 | 1 | -0.63 | 1 |
| 332 | Triglycerides | HDL cholesterol | -0.52 | 1 | -0.52 | 1 |
| 333 | Triglycerides | I48 Atrial fibrillation and flutter | -0.05 | 1 | -0.05 | 1 |
| 334 | Triglycerides | K41 Femoral hernia | 0.01 | 1 | 0.01 | 1 |
| 335 | Triglycerides | LDL direct | -0.64 | 1 | -0.64 | 1 |
| 336 | Triglycerides | Urate | 0.73 | 1 | - | 0 |
| 337 | Urate | Alkaline phosphatase | -0.77 | 1 | -0.77 | 1 |
| 338 | Urate | C-reactive protein | 0.49 | 1 | 0.49 | 1 |
| 339 | Urate | C16 Malignant neoplasm of stomach | 0.00 | 1 | 0.00 | 1 |
| 340 | Urate | Cystatin C | -0.38 | 1 | -0.38 | 1 |
| 341 | Urate | D04 Carcinoma in situ of skin | 0.00 | 1 | 0.00 | 1 |
| 342 | Urate | Gamma glutamyltransferase | 0.82 | 1 | 0.82 | 1 |
| 343 | Urate | Glycated haemoglobin | 0.21 | 1 | 0.21 | 1 |
| 344 | Urate | J31 Chronic rhinitis, nasopharyngitis and pharyngitis | 0.00 | 1 | 0.00 | 1 |
| 345 | Urate | K12 Stomatitis and related lesions | 0.00 | 1 | 0.00 | 1 |
| 346 | Urate | K74 Fibrosis and cirrhosis of liver | 0.00 | 1 | 0.00 | 1 |
| 347 | Urate | M81 Osteoporosis without pathological fracture | 0.01 | 1 | 0.01 | 1 |
| 348 | Urate | N62 Hypertrophy of breast | 0.00 | 1 | 0.00 | 1 |
| 349 | Urate | Phosphate | 0.63 | 1 | 0.63 | 1 |
| 350 | Urate | S30 Superficial injury of abdomen, lower back and pelvis | 0.00 | 1 | 0.00 | 1 |
| 351 | Urate | Total bilirubin | 0.29 | 1 | 0.29 | 1 |
| 352 | Urate | Z50 Care involving use of rehabilitation procedures | 0.00 | 1 | 0.00 | 1 |
| 353 | Urate | Z85 Personal history of malignant neoplasm | 0.00 | 1 | 0.00 | 1 |
| 354 | Urea | L43 Lichen planus | -0.01 | 1 | -0.01 | 1 |
| 355 | Urea | R25 Abnormal involuntary movements | -0.01 | 1 | -0.01 | 1 |
| 356 | Urea | S64 Injury of nerves at wrist and hand level | -0.01 | 1 | -0.01 | 1 |
| 357 | Urea | Total bilirubin | -0.55 | 1 | -0.55 | 1 |
| 358 | Vitamin D | Apoliprotein A | -1.12 | 1 | -1.12 | 1 |
| 359 | Vitamin D | Aspartate aminotransferase | -0.63 | 1 | -0.63 | 1 |
| 360 | Vitamin D | B37 Candidiasis | -0.01 | 1 | -0.01 | 1 |
| 361 | Vitamin D | Cholesterol | -1.49 | 1 | -1.49 | 1 |
| 362 | Vitamin D | G81 Hemiplegia | -0.01 | 1 | -0.01 | 1 |
| 363 | Vitamin D | M22 Disorders of patella | -0.01 | 1 | -0.01 | 1 |
| 364 | Vitamin D | R18 Ascites | -0.01 | 1 | -0.01 | 1 |
| 365 | Vitamin D | R26 Abnormalities of gait and mobility | 0.01 | 1 | 0.01 | 1 |
| 366 | Vitamin D | R30 Pain associated with micturition | 0.01 | 1 | 0.01 | 1 |
| 367 | Vitamin D | Triglycerides | -2.62 | 1 | -2.62 | 1 |
| 368 | Z11 Special screening examination for infectious and parasitic diseases | J03 Acute tonsillitis | 0.60 | 1 | 0.60 | 1 |
| 369 | Z11 Special screening examination for infectious and parasitic diseases | R25 Abnormal involuntary movements | 0.36 | 1 | 0.36 | 1 |
| 370 | Z11 Special screening examination for infectious and parasitic diseases | S80 Superficial injury of lower leg | -0.62 | 1 | -0.62 | 1 |
| 371 | Z50 Care involving use of rehabilitation procedures | Cystatin C | 52.86 | 1 | 52.86 | 1 |
| 372 | Z50 Care involving use of rehabilitation procedures | D11 Benign neoplasm of major salivary glands | 0.79 | 1 | 0.79 | 1 |
| 373 | Z50 Care involving use of rehabilitation procedures | S81 Open wound of lower leg | 0.75 | 1 | 0.75 | 1 |
| 374 | Z52 Donors of organs and tissues | D11 Benign neoplasm of major salivary glands | -0.70 | 1 | -0.70 | 1 |
| 375 | Z52 Donors of organs and tissues | Glucose | -21.63 | 1 | - | 0 |
| 376 | Z52 Donors of organs and tissues | Glycated haemoglobin | -24.40 | 1 | - | 0 |
| 377 | Z52 Donors of organs and tissues | IGF-1 | -21.19 | 1 | - | 0 |
| 378 | Z52 Donors of organs and tissues | M81 Osteoporosis without pathological fracture | -0.74 | 1 | -0.74 | 1 |
| 379 | Z52 Donors of organs and tissues | Urea | -14.78 | 1 | - | 0 |
| 380 | Z80 Family history of malignant neoplasm | Creatinine | -64.64 | 1 | -64.64 | 1 |
| 381 | Z80 Family history of malignant neoplasm | H81 Disorders of vestibular function | 0.51 | 1 | 0.51 | 1 |
| 382 | Z80 Family history of malignant neoplasm | J03 Acute tonsillitis | -1.08 | 1 | -1.08 | 1 |
| 383 | Z80 Family history of malignant neoplasm | R15 Faecal incontinence | -1.70 | 1 | -1.70 | 1 |
| 384 | Z80 Family history of malignant neoplasm | Urate | -51.29 | 1 | - | 0 |
| 385 | Z85 Personal history of malignant neoplasm | D04 Carcinoma in situ of skin | -0.58 | 1 | -0.58 | 1 |
| 386 | Z85 Personal history of malignant neoplasm | D11 Benign neoplasm of major salivary glands | 0.74 | 1 | 0.74 | 1 |
| 387 | Z85 Personal history of malignant neoplasm | I48 Atrial fibrillation and flutter | -2.51 | 1 | -2.51 | 1 |
| 388 | Z85 Personal history of malignant neoplasm | K90 Intestinal malabsorption | 0.53 | 1 | 0.53 | 1 |
